# Dataset of biomass characteristics and net output power from downdraft biomass gasifier integrated power production unit

**DOI:** 10.1016/j.dib.2020.106390

**Published:** 2020-10-09

**Authors:** Sahar Safarian, Seyed Mohammad Ebrahimi Saryazdi, Runar Unnthorsson, Christiaan Richter

**Affiliations:** a Faculty of Industrial Engineering, Mechanical Engineering and Computer Science, University of Iceland, Hjardarhagi 6, 107 Reykjavik, Iceland; b Department of Energy Systems Engineering, Sharif University of Technology, Tehran, Iran

**Keywords:** Biomass gasification, Net output power, Elemental analysis, Proximate analysis, Downdraft

## Abstract

This dataset includes 1032 runs from a biomass downdraft gasifier integrated with power production unit that is fed by 86 different types of biomasses from different groups (e.g. wood and woody biomasses, herbaceous and agricultural biomasses, animal biomasses, mixed biomasses and contaminated biomasses) and under various operating conditions. The dataset covers elemental and proximate analysis of various biomasses, operation conditions and the net output power from the biomass gasification-power production (BG-PP) in each case/run. This article has been submitted via another Elsevier journal as a co-submission, titled “Artificial neural network integrated with thermodynamic equilibrium modeling of downdraft biomass gasification-power production plant” [1]. In fact, this dataset has been used to train and test the developed Artificial neural network modeling of a downdraft BG-PP in our original research paper [1].

## Specifications Table

**Table:** 

Subject	Energy (Renewable Energy, Biomass Gasification and Power production)
Specific subject area	Data obtained from a downdraft biomass gasification-power production plant derived by various biomasses and different operating conditions
Type of data	Table
How data were acquired	Firstly, we developed a simulation model based on thermodynamic equilibrium for biomass gasification integrated power production unit by using ASPEN Plus. Then we extracted elemental and proximate analysis of 86 various biomasses from research work of Vassilev and et al., [2] (doi.org/10.1016/j.fuel.2009.10.022). These characteristics under various gasifier temperature and mass flow of air to fuel ratio (AFR) were entered to the simulation model as inputs. Finally, the net output power from the system was considered as system output.
Data format	Raw
Parameters for data collection	1032 case that each case has 12 data. Each case has different elemental analysis compositions (C, O, H, N and S), proximate analysis compositions (moisture, ash, volatile material and fixed carbon), operating parameters (gasifier temperature and air to fuel ratio) and net output power
Description of data collection	Firstly, a simulation model based on thermodynamic equilibrium for biomass gasification integrated power production unit was developed by using ASPEN Plus software. Then we extracted elemental and proximate analysis of 86 various biomasses from research work of Vassilev and et al., [2] (doi.org/10.1016/j.fuel.2009.10.022). These characteristics under various gasifier temperature and mass flow of air to fuel ratio (AFR) were entered to the simulation model as inputs. Finally, the net output power from the system was considered as system output.
Data source location	Institution: University of Iceland City/Town/Region: Reykjavik Country: Iceland Latitude and longitude (and GPS coordinates, if possible) for collected samples/data: 64.1466° N, 21.9426° W
Data accessibility	With the article and in a public repository with Mendeley Data Repository name: Dataset of biomass characteristics and net output power from downdraft biomass gasifier integrated power production unit Data identification number: Mendeley Data, V1 Direct URL to data: https://doi.org/10.17632/k78rj37kkg.2 and direct link to data: https://data.mendeley.com/datasets/k78rj37kkg/2
Related research article	S. Safarian, S.M. Ebrahimi Saryazdi, R. Unnthorsson, Ch. Richter Artificial neural network integrated with thermodynamic equilibrium modeling of downdraft biomass gasification-power production plant Energy Journal. (https://doi.org/10.1016/j.energy.2020.118800).

## Value of the Data

• This data is useful for training and testing of Artificial Neural Network (ANN) modeling of biomass downdraft gasification integrated with power production unit.
• This dataset has this potential to be used as a practical application in screening proper biomass feedstocks for energy extraction based on gasification technology integrated power unit.
• This data can be used by researchers of biofuels, anybody working on power generation by using biomass gasification and developers of Artificial Neural Network (ANN) modeling in field of biomass gasification.
• This dataset can be used for validation/verification of further simulation/numerical models and also experimental pilots of biomass gasification systems.

## 1 Data Description

This dataset is only one table, including 12 columns and 1032 rows. In fact, it concludes 1032 runs from a biomass downdraft gasifier integrated with power production unit (BG-PP) that is fed by 86 different types of biomasses from different groups. Columns are divided to elemental analysis compositions of biomasses (C, O, H, N and S), proximate analysis compositions of biomasses (moisture, ash, volatile material and fixed carbon) and operating parameters (gasifier temperature and air to fuel ratio). Rows are also dfferent types of biomasses.

## 2 Experimental Design, Materials and Methods

The developed simulation model for the downdraft biomass gasification integrated with power production (BG-PP) is based on the steady state stoichiometric equilibrium. This model has been developed by using ASPEN Plus software that is for chemical process simulation. This simulation model and its flow chart were shown in the our original research paper [1]. The equation of state of Penge Robinson with Boston-Mathias alpha function (PR-BM) was selected as the global property method for this model to calculate the physical properties of the conventional components in the gasification process. This method is suitable for the nonpolar or mildly polar mixtures such as hydrocarbons and light gasses. Because biomass and ash were defined as nonconventional components, only the density and enthalpy need to be calculated during the simulation. HCOALGEN and DCOALIGT models were applied for enthalpy and density of biomass and ash. Different empirical correlations for combustion heat, heat of formation and heat capacity are included in the HCOALGEN model. The density method DCOALIGT is also based on equations from IGT (Institute of Gas Technology). MCINCPSD stream comprising three substreams of MIXED, CIPSD and NCPSD class, was also considered to define the biomass structure and ash streams which are not available in Aspen Plus component database. This option is for the situation when there are both conventional and nonconventional solids, but no particle size distribution [3,4].

The biomass and ash streams need to be created by their components attributes that comprises the ultimate analysis, proximate analysis, and sulfur analysis. In this work, we considered 86 different types of biomasses from different groups (e.g. wood and woody biomasses, herbaceous and agricultural biomasses, animal biomasses, mixed biomasses and contaminated biomasses) [2], as feedstock for gasifier.

Drying is the first process in gasification that occurs at 150 °C to achieve the moisture reduction to 5 wt.% of the original sample. Usually, biomasses contain 5–60% moisture content that should be reduced in lower than 5% before entering to the gasifier. This step was carried out by using the RSTOIC that is a stoichiometric reactor in the Aspen Plus. This particular module is used to perform chemical reactions of known stoichiometry [5]. After drying, In the pyrolysis step, the biomass is heated to 500 °C with limited oxygen. Under these conditions the volatile components in the biomass are vaporized. The volatile materials (VM) is a mixture of hydrogen, carbon monoxide, carbon dioxide, methane, tar (heavier hydrocarbon) gasses, and water vapor. Tar (a black, viscous, and potentially corrosive liquid at standard temperature and pressure that is predominantly composed of heavy organic and inorganic molecules) and char (a solid residue mainly containing carbon) are also produced during pyrolysis [3]. Then RGibbs is used to simulate the biomass gasification. The reactor calculates the syngas composition by minimizing the Gibbs free energy and assumes complete chemical equilibrium. The decomposed feed and air enter to the RGibbs reactor where partial oxidation and gasification reactions occur. Another RGibbs reactor was also simulated for combustion section with pressure of 11 atm and with minimum air mixing. Principally, this process is also based on minimization of Gibbs free energy and its calculation is based on phase equilibrium and chemical equilibrium. The combustion chamber is followed by a gas turbine with pressure ratio of 0.5 and isentropic efficiency of 90% [6,7]. The thermal content of the gas, obtained as the combustion heat is recovered to preheat the entering air to the combustion chamber and also to meet the heat required in dryer. The recovered heat can be also consumed to convert water to high pressure steam though a HEATER and then the generated steam drives a steam turbine and produces additional power [8,9]. However, this part has not been consider in this work.

Since this model could be run, it needs characteristics of biomass feedstock and operating conditions through the system. Hence, as it mentioned above, 86 different biomasses with different characteristics from various groups under different operating conditions (gasifier temperature and mass flow of air to fuel ratio (AFR)) were entered to be gasified in a downdraft gasifier to produce power. It is important to say that all analysis is directed based on a functional unit of 1 ton for each input feedstock under atmospheric pressure and different operating conditions. Finally, the results from 1032 run/case including 11 inputs (moisture content (M), volatile materials (VM), fixed carbon (FC) and ash (A), carbon (C), oxygen (O), hydrogen (H), nitrogen (N), sulfur (S), gasifier temperature (T) and air to fuel ratio (AFR)) and 1 output (net output power) were organized our dataset. Table 1 lists the characteristics of the inputs and net output power ($W_{net}=W_{gasturbine}-W_{compressor}$) obtained from the simulation model results.

## Declaration of Competing Interest

The authors declare that they have no known competing financial interests or personal relationships which have, or could be perceived to have, influenced the work reported in this article.

## Appendix Supplementary materials

Image, application 1
