# Supplementary material for: Dataset of biomass characteristics and net output power from downdraft biomass gasifier integrated power production unit
Source: Data Brief. 2020 Oct 9;33:106390. doi: 10.1016/j.dib.2020.106390 (PMC7575841; doi:10.1016/j.dib.2020.106390)
Supplement: Supplementary file 1 [file mmc1.docx]

Table 1: Characteristics of various biomasses and net output power from biomass downdraft gasifier integrated with power production unit

| \|  \|  \| Proximate analysis (wt%) \| \| \| \| Elemental analysis (wt%- dry basis) \| \| \| \| \| Temperature \| Air to fuel mass flow ratio \| Net output power \| \| --- \| --- \| --- \| --- \| --- \| --- \| --- \| --- \| --- \| --- \| --- \| --- \| --- \| --- \| \| Biomass type \| \| M \| VM \| FC \| A \| C \| O \| H \| N \| S \| T (˚C) \| AFR (kg/kg) \| Wnet (kW) \| \| 1 \| Alder-fir sawdust \| 52,6 \| 76,6 \| 19,2 \| 4,2 \| 50,9656 \| 38,5116 \| 5,8438 \| 0,479 \| 0 \| 600 \| 1,8 \| 50,65 \| \|  \|  \| 52,6 \| 76,6 \| 19,2 \| 4,2 \| 50,9656 \| 38,5116 \| 5,8438 \| 0,479 \| 0 \| 600 \| 2 \| 47,49 \| \|  \|  \| 52,6 \| 76,6 \| 19,2 \| 4,2 \| 50,9656 \| 38,5116 \| 5,8438 \| 0,479 \| 0 \| 600 \| 2,3 \| 44,02 \| \|  \|  \| 52,6 \| 76,6 \| 19,2 \| 4,2 \| 50,9656 \| 38,5116 \| 5,8438 \| 0,479 \| 0 \| 900 \| 1,8 \| 103,09 \| \|  \|  \| 52,6 \| 76,6 \| 19,2 \| 4,2 \| 50,9656 \| 38,5116 \| 5,8438 \| 0,479 \| 0 \| 900 \| 2 \| 98,03 \| \|  \|  \| 52,6 \| 76,6 \| 19,2 \| 4,2 \| 50,9656 \| 38,5116 \| 5,8438 \| 0,479 \| 0 \| 900 \| 2,3 \| 92,03 \| \|  \|  \| 52,6 \| 76,6 \| 19,2 \| 4,2 \| 50,9656 \| 38,5116 \| 5,8438 \| 0,479 \| 0 \| 1200 \| 1,8 \| 120,70 \| \|  \|  \| 52,6 \| 76,6 \| 19,2 \| 4,2 \| 50,9656 \| 38,5116 \| 5,8438 \| 0,479 \| 0 \| 1200 \| 2 \| 117,97 \| \|  \|  \| 52,6 \| 76,6 \| 19,2 \| 4,2 \| 50,9656 \| 38,5116 \| 5,8438 \| 0,479 \| 0 \| 1200 \| 2,3 \| 114,25 \| \|  \|  \| 52,6 \| 76,6 \| 19,2 \| 4,2 \| 50,9656 \| 38,5116 \| 5,8438 \| 0,479 \| 0 \| 1500 \| 1,8 \| 137,59 \| \|  \|  \| 52,6 \| 76,6 \| 19,2 \| 4,2 \| 50,9656 \| 38,5116 \| 5,8438 \| 0,479 \| 0 \| 1500 \| 2 \| 137,10 \| \|  \|  \| 52,6 \| 76,6 \| 19,2 \| 4,2 \| 50,9656 \| 38,5116 \| 5,8438 \| 0,479 \| 0 \| 1500 \| 2,3 \| 135,64 \| \| 2 \| Balsam bark \| 8,4 \| 77,4 \| 20 \| 2,6 \| 52,596 \| 38,473 \| 6,0388 \| 0,1948 \| 0,0974 \| 600 \| 1,8 \| 119,08 \| \|  \|  \| 8,4 \| 77,4 \| 20 \| 2,6 \| 52,596 \| 38,473 \| 6,0388 \| 0,1948 \| 0,0974 \| 600 \| 2 \| 113,58 \| \|  \|  \| 8,4 \| 77,4 \| 20 \| 2,6 \| 52,596 \| 38,473 \| 6,0388 \| 0,1948 \| 0,0974 \| 600 \| 2,3 \| 107,33 \| \|  \|  \| 8,4 \| 77,4 \| 20 \| 2,6 \| 52,596 \| 38,473 \| 6,0388 \| 0,1948 \| 0,0974 \| 900 \| 1,8 \| 223,45 \| \|  \|  \| 8,4 \| 77,4 \| 20 \| 2,6 \| 52,596 \| 38,473 \| 6,0388 \| 0,1948 \| 0,0974 \| 900 \| 2 \| 215,42 \| \|  \|  \| 8,4 \| 77,4 \| 20 \| 2,6 \| 52,596 \| 38,473 \| 6,0388 \| 0,1948 \| 0,0974 \| 900 \| 2,3 \| 205,20 \| \|  \|  \| 8,4 \| 77,4 \| 20 \| 2,6 \| 52,596 \| 38,473 \| 6,0388 \| 0,1948 \| 0,0974 \| 1200 \| 1,8 \| 256,16 \| \|  \|  \| 8,4 \| 77,4 \| 20 \| 2,6 \| 52,596 \| 38,473 \| 6,0388 \| 0,1948 \| 0,0974 \| 1200 \| 2 \| 252,70 \| \|  \|  \| 8,4 \| 77,4 \| 20 \| 2,6 \| 52,596 \| 38,473 \| 6,0388 \| 0,1948 \| 0,0974 \| 1200 \| 2,3 \| 247,07 \| \|  \|  \| 8,4 \| 77,4 \| 20 \| 2,6 \| 52,596 \| 38,473 \| 6,0388 \| 0,1948 \| 0,0974 \| 1500 \| 1,8 \| 287,61 \| \|  \|  \| 8,4 \| 77,4 \| 20 \| 2,6 \| 52,596 \| 38,473 \| 6,0388 \| 0,1948 \| 0,0974 \| 1500 \| 2 \| 288,36 \| \|  \|  \| 8,4 \| 77,4 \| 20 \| 2,6 \| 52,596 \| 38,473 \| 6,0388 \| 0,1948 \| 0,0974 \| 1500 \| 2,3 \| 287,15 \| \| 3 \| Beech bark \| 8,4 \| 73,7 \| 18,5 \| 7,8 \| 47,3908 \| 38,5396 \| 5,532 \| 0,6454 \| 0,0922 \| 600 \| 1,8 \| 54,58 \| \|  \|  \| 8,4 \| 73,7 \| 18,5 \| 7,8 \| 47,3908 \| 38,5396 \| 5,532 \| 0,6454 \| 0,0922 \| 600 \| 2 \| 47,61 \| \|  \|  \| 8,4 \| 73,7 \| 18,5 \| 7,8 \| 47,3908 \| 38,5396 \| 5,532 \| 0,6454 \| 0,0922 \| 600 \| 2,3 \| 40,19 \| \|  \|  \| 8,4 \| 73,7 \| 18,5 \| 7,8 \| 47,3908 \| 38,5396 \| 5,532 \| 0,6454 \| 0,0922 \| 900 \| 1,8 \| 146,41 \| \|  \|  \| 8,4 \| 73,7 \| 18,5 \| 7,8 \| 47,3908 \| 38,5396 \| 5,532 \| 0,6454 \| 0,0922 \| 900 \| 2 \| 133,97 \| \|  \|  \| 8,4 \| 73,7 \| 18,5 \| 7,8 \| 47,3908 \| 38,5396 \| 5,532 \| 0,6454 \| 0,0922 \| 900 \| 2,3 \| 120,41 \| \|  \|  \| 8,4 \| 73,7 \| 18,5 \| 7,8 \| 47,3908 \| 38,5396 \| 5,532 \| 0,6454 \| 0,0922 \| 1200 \| 1,8 \| 182,75 \| \|  \|  \| 8,4 \| 73,7 \| 18,5 \| 7,8 \| 47,3908 \| 38,5396 \| 5,532 \| 0,6454 \| 0,0922 \| 1200 \| 2 \| 174,45 \| \|  \|  \| 8,4 \| 73,7 \| 18,5 \| 7,8 \| 47,3908 \| 38,5396 \| 5,532 \| 0,6454 \| 0,0922 \| 1200 \| 2,3 \| 164,80 \| \|  \|  \| 8,4 \| 73,7 \| 18,5 \| 7,8 \| 47,3908 \| 38,5396 \| 5,532 \| 0,6454 \| 0,0922 \| 1500 \| 1,8 \| 217,87 \| \|  \|  \| 8,4 \| 73,7 \| 18,5 \| 7,8 \| 47,3908 \| 38,5396 \| 5,532 \| 0,6454 \| 0,0922 \| 1500 \| 2 \| 213,74 \| \|  \|  \| 8,4 \| 73,7 \| 18,5 \| 7,8 \| 47,3908 \| 38,5396 \| 5,532 \| 0,6454 \| 0,0922 \| 1500 \| 2,3 \| 208,13 \| \| 4 \| Birch bark \| 8,4 \| 78,5 \| 19,4 \| 2,1 \| 55,803 \| 34,9503 \| 6,5593 \| 0,4895 \| 0,0979 \| 600 \| 1,8 \| 171,56 \| \|  \|  \| 8,4 \| 78,5 \| 19,4 \| 2,1 \| 55,803 \| 34,9503 \| 6,5593 \| 0,4895 \| 0,0979 \| 600 \| 2 \| 168,83 \| \|  \|  \| 8,4 \| 78,5 \| 19,4 \| 2,1 \| 55,803 \| 34,9503 \| 6,5593 \| 0,4895 \| 0,0979 \| 600 \| 2,3 \| 164,47 \| \|  \|  \| 8,4 \| 78,5 \| 19,4 \| 2,1 \| 55,803 \| 34,9503 \| 6,5593 \| 0,4895 \| 0,0979 \| 900 \| 1,8 \| 280,13 \| \|  \|  \| 8,4 \| 78,5 \| 19,4 \| 2,1 \| 55,803 \| 34,9503 \| 6,5593 \| 0,4895 \| 0,0979 \| 900 \| 2 \| 277,74 \| \|  \|  \| 8,4 \| 78,5 \| 19,4 \| 2,1 \| 55,803 \| 34,9503 \| 6,5593 \| 0,4895 \| 0,0979 \| 900 \| 2,3 \| 272,59 \| \|  \|  \| 8,4 \| 78,5 \| 19,4 \| 2,1 \| 55,803 \| 34,9503 \| 6,5593 \| 0,4895 \| 0,0979 \| 1200 \| 1,8 \| 309,38 \| \|  \|  \| 8,4 \| 78,5 \| 19,4 \| 2,1 \| 55,803 \| 34,9503 \| 6,5593 \| 0,4895 \| 0,0979 \| 1200 \| 2 \| 310,91 \| \|  \|  \| 8,4 \| 78,5 \| 19,4 \| 2,1 \| 55,803 \| 34,9503 \| 6,5593 \| 0,4895 \| 0,0979 \| 1200 \| 2,3 \| 310,21 \| \|  \|  \| 8,4 \| 78,5 \| 19,4 \| 2,1 \| 55,803 \| 34,9503 \| 6,5593 \| 0,4895 \| 0,0979 \| 1500 \| 1,8 \| 338,03 \| \|  \|  \| 8,4 \| 78,5 \| 19,4 \| 2,1 \| 55,803 \| 34,9503 \| 6,5593 \| 0,4895 \| 0,0979 \| 1500 \| 2 \| 343,00 \| \|  \|  \| 8,4 \| 78,5 \| 19,4 \| 2,1 \| 55,803 \| 34,9503 \| 6,5593 \| 0,4895 \| 0,0979 \| 1500 \| 2,3 \| 346,22 \| \| 5 \| Christmas trees \| 37,8 \| 74,2 \| 20,7 \| 5,1 \| 51,7205 \| 36,7263 \| 5,5991 \| 0,4745 \| 0,3796 \| 600 \| 1,8 \| 69,43 \| \|  \|  \| 37,8 \| 74,2 \| 20,7 \| 5,1 \| 51,7205 \| 36,7263 \| 5,5991 \| 0,4745 \| 0,3796 \| 600 \| 2 \| 65,41 \| \|  \|  \| 37,8 \| 74,2 \| 20,7 \| 5,1 \| 51,7205 \| 36,7263 \| 5,5991 \| 0,4745 \| 0,3796 \| 600 \| 2,3 \| 60,94 \| \|  \|  \| 37,8 \| 74,2 \| 20,7 \| 5,1 \| 51,7205 \| 36,7263 \| 5,5991 \| 0,4745 \| 0,3796 \| 900 \| 1,8 \| 140,06 \| \|  \|  \| 37,8 \| 74,2 \| 20,7 \| 5,1 \| 51,7205 \| 36,7263 \| 5,5991 \| 0,4745 \| 0,3796 \| 900 \| 2 \| 133,86 \| \|  \|  \| 37,8 \| 74,2 \| 20,7 \| 5,1 \| 51,7205 \| 36,7263 \| 5,5991 \| 0,4745 \| 0,3796 \| 900 \| 2,3 \| 126,32 \| \|  \|  \| 37,8 \| 74,2 \| 20,7 \| 5,1 \| 51,7205 \| 36,7263 \| 5,5991 \| 0,4745 \| 0,3796 \| 1200 \| 1,8 \| 162,39 \| \|  \|  \| 37,8 \| 74,2 \| 20,7 \| 5,1 \| 51,7205 \| 36,7263 \| 5,5991 \| 0,4745 \| 0,3796 \| 1200 \| 2 \| 159,29 \| \|  \|  \| 37,8 \| 74,2 \| 20,7 \| 5,1 \| 51,7205 \| 36,7263 \| 5,5991 \| 0,4745 \| 0,3796 \| 1200 \| 2,3 \| 154,78 \| \|  \|  \| 37,8 \| 74,2 \| 20,7 \| 5,1 \| 51,7205 \| 36,7263 \| 5,5991 \| 0,4745 \| 0,3796 \| 1500 \| 1,8 \| 183,88 \| \|  \|  \| 37,8 \| 74,2 \| 20,7 \| 5,1 \| 51,7205 \| 36,7263 \| 5,5991 \| 0,4745 \| 0,3796 \| 1500 \| 2 \| 183,68 \| \|  \|  \| 37,8 \| 74,2 \| 20,7 \| 5,1 \| 51,7205 \| 36,7263 \| 5,5991 \| 0,4745 \| 0,3796 \| 1500 \| 2,3 \| 182,17 \| \| 6 \| Elm bark \| 8,4 \| 73,1 \| 18,8 \| 8,1 \| 46,7771 \| 39,0575 \| 5,3302 \| 0,6433 \| 0,0919 \| 600 \| 1,8 \| 40,09 \| \|  \|  \| 8,4 \| 73,1 \| 18,8 \| 8,1 \| 46,7771 \| 39,0575 \| 5,3302 \| 0,6433 \| 0,0919 \| 600 \| 2 \| 32,89 \| \|  \|  \| 8,4 \| 73,1 \| 18,8 \| 8,1 \| 46,7771 \| 39,0575 \| 5,3302 \| 0,6433 \| 0,0919 \| 600 \| 2,3 \| 25,28 \| \|  \|  \| 8,4 \| 73,1 \| 18,8 \| 8,1 \| 46,7771 \| 39,0575 \| 5,3302 \| 0,6433 \| 0,0919 \| 900 \| 1,8 \| 129,03 \| \|  \|  \| 8,4 \| 73,1 \| 18,8 \| 8,1 \| 46,7771 \| 39,0575 \| 5,3302 \| 0,6433 \| 0,0919 \| 900 \| 2 \| 115,99 \| \|  \|  \| 8,4 \| 73,1 \| 18,8 \| 8,1 \| 46,7771 \| 39,0575 \| 5,3302 \| 0,6433 \| 0,0919 \| 900 \| 2,3 \| 101,96 \| \|  \|  \| 8,4 \| 73,1 \| 18,8 \| 8,1 \| 46,7771 \| 39,0575 \| 5,3302 \| 0,6433 \| 0,0919 \| 1200 \| 1,8 \| 165,85 \| \|  \|  \| 8,4 \| 73,1 \| 18,8 \| 8,1 \| 46,7771 \| 39,0575 \| 5,3302 \| 0,6433 \| 0,0919 \| 1200 \| 2 \| 156,80 \| \|  \|  \| 8,4 \| 73,1 \| 18,8 \| 8,1 \| 46,7771 \| 39,0575 \| 5,3302 \| 0,6433 \| 0,0919 \| 1200 \| 2,3 \| 146,57 \| \|  \|  \| 8,4 \| 73,1 \| 18,8 \| 8,1 \| 46,7771 \| 39,0575 \| 5,3302 \| 0,6433 \| 0,0919 \| 1500 \| 1,8 \| 201,55 \| \|  \|  \| 8,4 \| 73,1 \| 18,8 \| 8,1 \| 46,7771 \| 39,0575 \| 5,3302 \| 0,6433 \| 0,0919 \| 1500 \| 2 \| 196,57 \| \|  \|  \| 8,4 \| 73,1 \| 18,8 \| 8,1 \| 46,7771 \| 39,0575 \| 5,3302 \| 0,6433 \| 0,0919 \| 1500 \| 2,3 \| 190,27 \| \| 7 \| Eucalyptus bark \| 12 \| 78 \| 17,2 \| 4,8 \| 46,3624 \| 43,1256 \| 5,4264 \| 0,2856 \| 0 \| 600 \| 1,8 \| 25,77 \| \|  \|  \| 12 \| 78 \| 17,2 \| 4,8 \| 46,3624 \| 43,1256 \| 5,4264 \| 0,2856 \| 0 \| 600 \| 2 \| 18,67 \| \|  \|  \| 12 \| 78 \| 17,2 \| 4,8 \| 46,3624 \| 43,1256 \| 5,4264 \| 0,2856 \| 0 \| 600 \| 2,3 \| 11,19 \| \|  \|  \| 12 \| 78 \| 17,2 \| 4,8 \| 46,3624 \| 43,1256 \| 5,4264 \| 0,2856 \| 0 \| 900 \| 1,8 \| 105,64 \| \|  \|  \| 12 \| 78 \| 17,2 \| 4,8 \| 46,3624 \| 43,1256 \| 5,4264 \| 0,2856 \| 0 \| 900 \| 2 \| 92,54 \| \|  \|  \| 12 \| 78 \| 17,2 \| 4,8 \| 46,3624 \| 43,1256 \| 5,4264 \| 0,2856 \| 0 \| 900 \| 2,3 \| 78,64 \| \|  \|  \| 12 \| 78 \| 17,2 \| 4,8 \| 46,3624 \| 43,1256 \| 5,4264 \| 0,2856 \| 0 \| 1200 \| 1,8 \| 142,32 \| \|  \|  \| 12 \| 78 \| 17,2 \| 4,8 \| 46,3624 \| 43,1256 \| 5,4264 \| 0,2856 \| 0 \| 1200 \| 2 \| 132,90 \| \|  \|  \| 12 \| 78 \| 17,2 \| 4,8 \| 46,3624 \| 43,1256 \| 5,4264 \| 0,2856 \| 0 \| 1200 \| 2,3 \| 122,51 \| \|  \|  \| 12 \| 78 \| 17,2 \| 4,8 \| 46,3624 \| 43,1256 \| 5,4264 \| 0,2856 \| 0 \| 1500 \| 1,8 \| 177,99 \| \|  \|  \| 12 \| 78 \| 17,2 \| 4,8 \| 46,3624 \| 43,1256 \| 5,4264 \| 0,2856 \| 0 \| 1500 \| 2 \| 172,34 \| \|  \|  \| 12 \| 78 \| 17,2 \| 4,8 \| 46,3624 \| 43,1256 \| 5,4264 \| 0,2856 \| 0 \| 1500 \| 2,3 \| 165,59 \| \| 8 \| Fir mill residue \| 62,9 \| 82 \| 17,5 \| 0,5 \| 51,143 \| 42,2875 \| 5,97 \| 0,0995 \| 0 \| 600 \| 1,8 \| 37,54 \| \|  \|  \| 62,9 \| 82 \| 17,5 \| 0,5 \| 51,143 \| 42,2875 \| 5,97 \| 0,0995 \| 0 \| 600 \| 2 \| 35,01 \| \|  \|  \| 62,9 \| 82 \| 17,5 \| 0,5 \| 51,143 \| 42,2875 \| 5,97 \| 0,0995 \| 0 \| 600 \| 2,3 \| 32,25 \| \|  \|  \| 62,9 \| 82 \| 17,5 \| 0,5 \| 51,143 \| 42,2875 \| 5,97 \| 0,0995 \| 0 \| 900 \| 1,8 \| 77,52 \| \|  \|  \| 62,9 \| 82 \| 17,5 \| 0,5 \| 51,143 \| 42,2875 \| 5,97 \| 0,0995 \| 0 \| 900 \| 2 \| 73,30 \| \|  \|  \| 62,9 \| 82 \| 17,5 \| 0,5 \| 51,143 \| 42,2875 \| 5,97 \| 0,0995 \| 0 \| 900 \| 2,3 \| 68,41 \| \|  \|  \| 62,9 \| 82 \| 17,5 \| 0,5 \| 51,143 \| 42,2875 \| 5,97 \| 0,0995 \| 0 \| 1200 \| 1,8 \| 91,79 \| \|  \|  \| 62,9 \| 82 \| 17,5 \| 0,5 \| 51,143 \| 42,2875 \| 5,97 \| 0,0995 \| 0 \| 1200 \| 2 \| 89,39 \| \|  \|  \| 62,9 \| 82 \| 17,5 \| 0,5 \| 51,143 \| 42,2875 \| 5,97 \| 0,0995 \| 0 \| 1200 \| 2,3 \| 86,24 \| \|  \|  \| 62,9 \| 82 \| 17,5 \| 0,5 \| 51,143 \| 42,2875 \| 5,97 \| 0,0995 \| 0 \| 1500 \| 1,8 \| 105,46 \| \|  \|  \| 62,9 \| 82 \| 17,5 \| 0,5 \| 51,143 \| 42,2875 \| 5,97 \| 0,0995 \| 0 \| 1500 \| 2 \| 104,82 \| \|  \|  \| 62,9 \| 82 \| 17,5 \| 0,5 \| 51,143 \| 42,2875 \| 5,97 \| 0,0995 \| 0 \| 1500 \| 2,3 \| 103,44 \| \| 9 \| Forest residue \| 56,8 \| 79,9 \| 16,9 \| 3,2 \| 51,0136 \| 39,7848 \| 5,2272 \| 0,6776 \| 0,0968 \| 600 \| 1,8 \| 34,66 \| \|  \|  \| 56,8 \| 79,9 \| 16,9 \| 3,2 \| 51,0136 \| 39,7848 \| 5,2272 \| 0,6776 \| 0,0968 \| 600 \| 2 \| 31,56 \| \|  \|  \| 56,8 \| 79,9 \| 16,9 \| 3,2 \| 51,0136 \| 39,7848 \| 5,2272 \| 0,6776 \| 0,0968 \| 600 \| 2,3 \| 28,23 \| \|  \|  \| 56,8 \| 79,9 \| 16,9 \| 3,2 \| 51,0136 \| 39,7848 \| 5,2272 \| 0,6776 \| 0,0968 \| 900 \| 1,8 \| 81,75 \| \|  \|  \| 56,8 \| 79,9 \| 16,9 \| 3,2 \| 51,0136 \| 39,7848 \| 5,2272 \| 0,6776 \| 0,0968 \| 900 \| 2 \| 76,47 \| \|  \|  \| 56,8 \| 79,9 \| 16,9 \| 3,2 \| 51,0136 \| 39,7848 \| 5,2272 \| 0,6776 \| 0,0968 \| 900 \| 2,3 \| 70,50 \| \|  \|  \| 56,8 \| 79,9 \| 16,9 \| 3,2 \| 51,0136 \| 39,7848 \| 5,2272 \| 0,6776 \| 0,0968 \| 1200 \| 1,8 \| 98,16 \| \|  \|  \| 56,8 \| 79,9 \| 16,9 \| 3,2 \| 51,0136 \| 39,7848 \| 5,2272 \| 0,6776 \| 0,0968 \| 1200 \| 2 \| 94,95 \| \|  \|  \| 56,8 \| 79,9 \| 16,9 \| 3,2 \| 51,0136 \| 39,7848 \| 5,2272 \| 0,6776 \| 0,0968 \| 1200 \| 2,3 \| 90,94 \| \|  \|  \| 56,8 \| 79,9 \| 16,9 \| 3,2 \| 51,0136 \| 39,7848 \| 5,2272 \| 0,6776 \| 0,0968 \| 1500 \| 1,8 \| 113,95 \| \|  \|  \| 56,8 \| 79,9 \| 16,9 \| 3,2 \| 51,0136 \| 39,7848 \| 5,2272 \| 0,6776 \| 0,0968 \| 1500 \| 2 \| 112,77 \| \|  \|  \| 56,8 \| 79,9 \| 16,9 \| 3,2 \| 51,0136 \| 39,7848 \| 5,2272 \| 0,6776 \| 0,0968 \| 1500 \| 2,3 \| 110,78 \| \| 10 \| Hemlock bark \| 8,4 \| 72 \| 25,5 \| 2,5 \| 53,625 \| 37,83 \| 5,7525 \| 0,195 \| 0,0975 \| 600 \| 1,8 \| 120,13 \| \|  \|  \| 8,4 \| 72 \| 25,5 \| 2,5 \| 53,625 \| 37,83 \| 5,7525 \| 0,195 \| 0,0975 \| 600 \| 2 \| 114,75 \| \|  \|  \| 8,4 \| 72 \| 25,5 \| 2,5 \| 53,625 \| 37,83 \| 5,7525 \| 0,195 \| 0,0975 \| 600 \| 2,3 \| 108,58 \| \|  \|  \| 8,4 \| 72 \| 25,5 \| 2,5 \| 53,625 \| 37,83 \| 5,7525 \| 0,195 \| 0,0975 \| 900 \| 1,8 \| 226,79 \| \|  \|  \| 8,4 \| 72 \| 25,5 \| 2,5 \| 53,625 \| 37,83 \| 5,7525 \| 0,195 \| 0,0975 \| 900 \| 2 \| 219,22 \| \|  \|  \| 8,4 \| 72 \| 25,5 \| 2,5 \| 53,625 \| 37,83 \| 5,7525 \| 0,195 \| 0,0975 \| 900 \| 2,3 \| 209,36 \| \|  \|  \| 8,4 \| 72 \| 25,5 \| 2,5 \| 53,625 \| 37,83 \| 5,7525 \| 0,195 \| 0,0975 \| 1200 \| 1,8 \| 258,71 \| \|  \|  \| 8,4 \| 72 \| 25,5 \| 2,5 \| 53,625 \| 37,83 \| 5,7525 \| 0,195 \| 0,0975 \| 1200 \| 2 \| 255,69 \| \|  \|  \| 8,4 \| 72 \| 25,5 \| 2,5 \| 53,625 \| 37,83 \| 5,7525 \| 0,195 \| 0,0975 \| 1200 \| 2,3 \| 250,45 \| \|  \|  \| 8,4 \| 72 \| 25,5 \| 2,5 \| 53,625 \| 37,83 \| 5,7525 \| 0,195 \| 0,0975 \| 1500 \| 1,8 \| 289,47 \| \|  \|  \| 8,4 \| 72 \| 25,5 \| 2,5 \| 53,625 \| 37,83 \| 5,7525 \| 0,195 \| 0,0975 \| 1500 \| 2 \| 290,61 \| \|  \|  \| 8,4 \| 72 \| 25,5 \| 2,5 \| 53,625 \| 37,83 \| 5,7525 \| 0,195 \| 0,0975 \| 1500 \| 2,3 \| 289,78 \| \| 11 \| Land clearing wood \| 49,2 \| 69,7 \| 13,8 \| 16,5 \| 42,3345 \| 35,738 \| 5,01 \| 0,334 \| 0,0835 \| 600 \| 1,8 \| 0,00 \| \|  \|  \| 49,2 \| 69,7 \| 13,8 \| 16,5 \| 42,3345 \| 35,738 \| 5,01 \| 0,334 \| 0,0835 \| 600 \| 2 \| 0,00 \| \|  \|  \| 49,2 \| 69,7 \| 13,8 \| 16,5 \| 42,3345 \| 35,738 \| 5,01 \| 0,334 \| 0,0835 \| 600 \| 2,3 \| 0,00 \| \|  \|  \| 49,2 \| 69,7 \| 13,8 \| 16,5 \| 42,3345 \| 35,738 \| 5,01 \| 0,334 \| 0,0835 \| 900 \| 1,8 \| 41,06 \| \|  \|  \| 49,2 \| 69,7 \| 13,8 \| 16,5 \| 42,3345 \| 35,738 \| 5,01 \| 0,334 \| 0,0835 \| 900 \| 2 \| 33,06 \| \|  \|  \| 49,2 \| 69,7 \| 13,8 \| 16,5 \| 42,3345 \| 35,738 \| 5,01 \| 0,334 \| 0,0835 \| 900 \| 2,3 \| 24,66 \| \|  \|  \| 49,2 \| 69,7 \| 13,8 \| 16,5 \| 42,3345 \| 35,738 \| 5,01 \| 0,334 \| 0,0835 \| 1200 \| 1,8 \| 61,74 \| \|  \|  \| 49,2 \| 69,7 \| 13,8 \| 16,5 \| 42,3345 \| 35,738 \| 5,01 \| 0,334 \| 0,0835 \| 1200 \| 2 \| 55,76 \| \|  \|  \| 49,2 \| 69,7 \| 13,8 \| 16,5 \| 42,3345 \| 35,738 \| 5,01 \| 0,334 \| 0,0835 \| 1200 \| 2,3 \| 49,33 \| \|  \|  \| 49,2 \| 69,7 \| 13,8 \| 16,5 \| 42,3345 \| 35,738 \| 5,01 \| 0,334 \| 0,0835 \| 1500 \| 1,8 \| 82,02 \| \|  \|  \| 49,2 \| 69,7 \| 13,8 \| 16,5 \| 42,3345 \| 35,738 \| 5,01 \| 0,334 \| 0,0835 \| 1500 \| 2 \| 78,10 \| \|  \|  \| 49,2 \| 69,7 \| 13,8 \| 16,5 \| 42,3345 \| 35,738 \| 5,01 \| 0,334 \| 0,0835 \| 1500 \| 2,3 \| 73,68 \| \| 12 \| Maple bark \| 8,4 \| 76,6 \| 19,4 \| 4 \| 49,92 \| 39,648 \| 5,952 \| 0,384 \| 0,096 \| 600 \| 1,8 \| 89,11 \| \|  \|  \| 8,4 \| 76,6 \| 19,4 \| 4 \| 49,92 \| 39,648 \| 5,952 \| 0,384 \| 0,096 \| 600 \| 2 \| 82,77 \| \|  \|  \| 8,4 \| 76,6 \| 19,4 \| 4 \| 49,92 \| 39,648 \| 5,952 \| 0,384 \| 0,096 \| 600 \| 2,3 \| 75,89 \| \|  \|  \| 8,4 \| 76,6 \| 19,4 \| 4 \| 49,92 \| 39,648 \| 5,952 \| 0,384 \| 0,096 \| 900 \| 1,8 \| 187,16 \| \|  \|  \| 8,4 \| 76,6 \| 19,4 \| 4 \| 49,92 \| 39,648 \| 5,952 \| 0,384 \| 0,096 \| 900 \| 2 \| 176,57 \| \|  \|  \| 8,4 \| 76,6 \| 19,4 \| 4 \| 49,92 \| 39,648 \| 5,952 \| 0,384 \| 0,096 \| 900 \| 2,3 \| 164,38 \| \|  \|  \| 8,4 \| 76,6 \| 19,4 \| 4 \| 49,92 \| 39,648 \| 5,952 \| 0,384 \| 0,096 \| 1200 \| 1,8 \| 222,26 \| \|  \|  \| 8,4 \| 76,6 \| 19,4 \| 4 \| 49,92 \| 39,648 \| 5,952 \| 0,384 \| 0,096 \| 1200 \| 2 \| 216,11 \| \|  \|  \| 8,4 \| 76,6 \| 19,4 \| 4 \| 49,92 \| 39,648 \| 5,952 \| 0,384 \| 0,096 \| 1200 \| 2,3 \| 208,18 \| \|  \|  \| 8,4 \| 76,6 \| 19,4 \| 4 \| 49,92 \| 39,648 \| 5,952 \| 0,384 \| 0,096 \| 1500 \| 1,8 \| 255,94 \| \|  \|  \| 8,4 \| 76,6 \| 19,4 \| 4 \| 49,92 \| 39,648 \| 5,952 \| 0,384 \| 0,096 \| 1500 \| 2 \| 254,11 \| \|  \|  \| 8,4 \| 76,6 \| 19,4 \| 4 \| 49,92 \| 39,648 \| 5,952 \| 0,384 \| 0,096 \| 1500 \| 2,3 \| 250,50 \| \| 13 \| Oak sawdust \| 11,5 \| 86,3 \| 13,4 \| 0,3 \| 49,9497 \| 43,7683 \| 5,8823 \| 0,0997 \| 0 \| 600 \| 1,8 \| 71,88 \| \|  \|  \| 11,5 \| 86,3 \| 13,4 \| 0,3 \| 49,9497 \| 43,7683 \| 5,8823 \| 0,0997 \| 0 \| 600 \| 2 \| 65,49 \| \|  \|  \| 11,5 \| 86,3 \| 13,4 \| 0,3 \| 49,9497 \| 43,7683 \| 5,8823 \| 0,0997 \| 0 \| 600 \| 2,3 \| 58,63 \| \|  \|  \| 11,5 \| 86,3 \| 13,4 \| 0,3 \| 49,9497 \| 43,7683 \| 5,8823 \| 0,0997 \| 0 \| 900 \| 1,8 \| 162,87 \| \|  \|  \| 11,5 \| 86,3 \| 13,4 \| 0,3 \| 49,9497 \| 43,7683 \| 5,8823 \| 0,0997 \| 0 \| 900 \| 2 \| 151,67 \| \|  \|  \| 11,5 \| 86,3 \| 13,4 \| 0,3 \| 49,9497 \| 43,7683 \| 5,8823 \| 0,0997 \| 0 \| 900 \| 2,3 \| 139,19 \| \|  \|  \| 11,5 \| 86,3 \| 13,4 \| 0,3 \| 49,9497 \| 43,7683 \| 5,8823 \| 0,0997 \| 0 \| 1200 \| 1,8 \| 198,27 \| \|  \|  \| 11,5 \| 86,3 \| 13,4 \| 0,3 \| 49,9497 \| 43,7683 \| 5,8823 \| 0,0997 \| 0 \| 1200 \| 2 \| 191,21 \| \|  \|  \| 11,5 \| 86,3 \| 13,4 \| 0,3 \| 49,9497 \| 43,7683 \| 5,8823 \| 0,0997 \| 0 \| 1200 \| 2,3 \| 182,65 \| \|  \|  \| 11,5 \| 86,3 \| 13,4 \| 0,3 \| 49,9497 \| 43,7683 \| 5,8823 \| 0,0997 \| 0 \| 1500 \| 1,8 \| 232,26 \| \|  \|  \| 11,5 \| 86,3 \| 13,4 \| 0,3 \| 49,9497 \| 43,7683 \| 5,8823 \| 0,0997 \| 0 \| 1500 \| 2 \| 229,33 \| \|  \|  \| 11,5 \| 86,3 \| 13,4 \| 0,3 \| 49,9497 \| 43,7683 \| 5,8823 \| 0,0997 \| 0 \| 1500 \| 2,3 \| 224,82 \| \| 14 \| Oak wood \| 6,5 \| 78,1 \| 21,4 \| 0,5 \| 50,347 \| 42,6855 \| 6,0695 \| 0,2985 \| 0,0995 \| 600 \| 1,8 \| 89,96 \| \|  \|  \| 6,5 \| 78,1 \| 21,4 \| 0,5 \| 50,347 \| 42,6855 \| 6,0695 \| 0,2985 \| 0,0995 \| 600 \| 2 \| 83,46 \| \|  \|  \| 6,5 \| 78,1 \| 21,4 \| 0,5 \| 50,347 \| 42,6855 \| 6,0695 \| 0,2985 \| 0,0995 \| 600 \| 2,3 \| 76,43 \| \|  \|  \| 6,5 \| 78,1 \| 21,4 \| 0,5 \| 50,347 \| 42,6855 \| 6,0695 \| 0,2985 \| 0,0995 \| 900 \| 1,8 \| 188,44 \| \|  \|  \| 6,5 \| 78,1 \| 21,4 \| 0,5 \| 50,347 \| 42,6855 \| 6,0695 \| 0,2985 \| 0,0995 \| 900 \| 2 \| 177,40 \| \|  \|  \| 6,5 \| 78,1 \| 21,4 \| 0,5 \| 50,347 \| 42,6855 \| 6,0695 \| 0,2985 \| 0,0995 \| 900 \| 2,3 \| 164,80 \| \|  \|  \| 6,5 \| 78,1 \| 21,4 \| 0,5 \| 50,347 \| 42,6855 \| 6,0695 \| 0,2985 \| 0,0995 \| 1200 \| 1,8 \| 225,11 \| \|  \|  \| 6,5 \| 78,1 \| 21,4 \| 0,5 \| 50,347 \| 42,6855 \| 6,0695 \| 0,2985 \| 0,0995 \| 1200 \| 2 \| 218,56 \| \|  \|  \| 6,5 \| 78,1 \| 21,4 \| 0,5 \| 50,347 \| 42,6855 \| 6,0695 \| 0,2985 \| 0,0995 \| 1200 \| 2,3 \| 210,26 \| \|  \|  \| 6,5 \| 78,1 \| 21,4 \| 0,5 \| 50,347 \| 42,6855 \| 6,0695 \| 0,2985 \| 0,0995 \| 1500 \| 1,8 \| 260,24 \| \|  \|  \| 6,5 \| 78,1 \| 21,4 \| 0,5 \| 50,347 \| 42,6855 \| 6,0695 \| 0,2985 \| 0,0995 \| 1500 \| 2 \| 258,11 \| \|  \|  \| 6,5 \| 78,1 \| 21,4 \| 0,5 \| 50,347 \| 42,6855 \| 6,0695 \| 0,2985 \| 0,0995 \| 1500 \| 2,3 \| 254,20 \| \| 15 \| Olive wood \| 6,6 \| 79,6 \| 17,2 \| 3,2 \| 47,432 \| 43,4632 \| 5,2272 \| 0,6776 \| 0 \| 600 \| 1,8 \| 29,49 \| \|  \|  \| 6,6 \| 79,6 \| 17,2 \| 3,2 \| 47,432 \| 43,4632 \| 5,2272 \| 0,6776 \| 0 \| 600 \| 2 \| 22,01 \| \|  \|  \| 6,6 \| 79,6 \| 17,2 \| 3,2 \| 47,432 \| 43,4632 \| 5,2272 \| 0,6776 \| 0 \| 600 \| 2,3 \| 14,11 \| \|  \|  \| 6,6 \| 79,6 \| 17,2 \| 3,2 \| 47,432 \| 43,4632 \| 5,2272 \| 0,6776 \| 0 \| 900 \| 1,8 \| 116,73 \| \|  \|  \| 6,6 \| 79,6 \| 17,2 \| 3,2 \| 47,432 \| 43,4632 \| 5,2272 \| 0,6776 \| 0 \| 900 \| 2 \| 102,93 \| \|  \|  \| 6,6 \| 79,6 \| 17,2 \| 3,2 \| 47,432 \| 43,4632 \| 5,2272 \| 0,6776 \| 0 \| 900 \| 2,3 \| 88,26 \| \|  \|  \| 6,6 \| 79,6 \| 17,2 \| 3,2 \| 47,432 \| 43,4632 \| 5,2272 \| 0,6776 \| 0 \| 1200 \| 1,8 \| 155,36 \| \|  \|  \| 6,6 \| 79,6 \| 17,2 \| 3,2 \| 47,432 \| 43,4632 \| 5,2272 \| 0,6776 \| 0 \| 1200 \| 2 \| 145,51 \| \|  \|  \| 6,6 \| 79,6 \| 17,2 \| 3,2 \| 47,432 \| 43,4632 \| 5,2272 \| 0,6776 \| 0 \| 1200 \| 2,3 \| 134,60 \| \|  \|  \| 6,6 \| 79,6 \| 17,2 \| 3,2 \| 47,432 \| 43,4632 \| 5,2272 \| 0,6776 \| 0 \| 1500 \| 1,8 \| 192,90 \| \|  \|  \| 6,6 \| 79,6 \| 17,2 \| 3,2 \| 47,432 \| 43,4632 \| 5,2272 \| 0,6776 \| 0 \| 1500 \| 2 \| 187,10 \| \|  \|  \| 6,6 \| 79,6 \| 17,2 \| 3,2 \| 47,432 \| 43,4632 \| 5,2272 \| 0,6776 \| 0 \| 1500 \| 2,3 \| 180,07 \| \| 16 \| Pine bark \| 4,7 \| 73,7 \| 24,4 \| 1,9 \| 52,7778 \| 39,1419 \| 5,7879 \| 0,2943 \| 0,0981 \| 600 \| 1,8 \| 106,86 \| \|  \|  \| 4,7 \| 73,7 \| 24,4 \| 1,9 \| 52,7778 \| 39,1419 \| 5,7879 \| 0,2943 \| 0,0981 \| 600 \| 2 \| 100,67 \| \|  \|  \| 4,7 \| 73,7 \| 24,4 \| 1,9 \| 52,7778 \| 39,1419 \| 5,7879 \| 0,2943 \| 0,0981 \| 600 \| 2,3 \| 93,82 \| \|  \|  \| 4,7 \| 73,7 \| 24,4 \| 1,9 \| 52,7778 \| 39,1419 \| 5,7879 \| 0,2943 \| 0,0981 \| 900 \| 1,8 \| 214,50 \| \|  \|  \| 4,7 \| 73,7 \| 24,4 \| 1,9 \| 52,7778 \| 39,1419 \| 5,7879 \| 0,2943 \| 0,0981 \| 900 \| 2 \| 204,90 \| \|  \|  \| 4,7 \| 73,7 \| 24,4 \| 1,9 \| 52,7778 \| 39,1419 \| 5,7879 \| 0,2943 \| 0,0981 \| 900 \| 2,3 \| 193,25 \| \|  \|  \| 4,7 \| 73,7 \| 24,4 \| 1,9 \| 52,7778 \| 39,1419 \| 5,7879 \| 0,2943 \| 0,0981 \| 1200 \| 1,8 \| 249,19 \| \|  \|  \| 4,7 \| 73,7 \| 24,4 \| 1,9 \| 52,7778 \| 39,1419 \| 5,7879 \| 0,2943 \| 0,0981 \| 1200 \| 2 \| 244,32 \| \|  \|  \| 4,7 \| 73,7 \| 24,4 \| 1,9 \| 52,7778 \| 39,1419 \| 5,7879 \| 0,2943 \| 0,0981 \| 1200 \| 2,3 \| 237,32 \| \|  \|  \| 4,7 \| 73,7 \| 24,4 \| 1,9 \| 52,7778 \| 39,1419 \| 5,7879 \| 0,2943 \| 0,0981 \| 1500 \| 1,8 \| 282,51 \| \|  \|  \| 4,7 \| 73,7 \| 24,4 \| 1,9 \| 52,7778 \| 39,1419 \| 5,7879 \| 0,2943 \| 0,0981 \| 1500 \| 2 \| 282,10 \| \|  \|  \| 4,7 \| 73,7 \| 24,4 \| 1,9 \| 52,7778 \| 39,1419 \| 5,7879 \| 0,2943 \| 0,0981 \| 1500 \| 2,3 \| 279,70 \| \| 17 \| Pine chips \| 7,6 \| 72,4 \| 21,6 \| 6 \| 49,632 \| 38,07 \| 5,734 \| 0,47 \| 0,094 \| 600 \| 1,8 \| 84,31 \| \|  \|  \| 7,6 \| 72,4 \| 21,6 \| 6 \| 49,632 \| 38,07 \| 5,734 \| 0,47 \| 0,094 \| 600 \| 2 \| 77,82 \| \|  \|  \| 7,6 \| 72,4 \| 21,6 \| 6 \| 49,632 \| 38,07 \| 5,734 \| 0,47 \| 0,094 \| 600 \| 2,3 \| 70,80 \| \|  \|  \| 7,6 \| 72,4 \| 21,6 \| 6 \| 49,632 \| 38,07 \| 5,734 \| 0,47 \| 0,094 \| 900 \| 1,8 \| 183,73 \| \|  \|  \| 7,6 \| 72,4 \| 21,6 \| 6 \| 49,632 \| 38,07 \| 5,734 \| 0,47 \| 0,094 \| 900 \| 2 \| 172,86 \| \|  \|  \| 7,6 \| 72,4 \| 21,6 \| 6 \| 49,632 \| 38,07 \| 5,734 \| 0,47 \| 0,094 \| 900 \| 2,3 \| 160,41 \| \|  \|  \| 7,6 \| 72,4 \| 21,6 \| 6 \| 49,632 \| 38,07 \| 5,734 \| 0,47 \| 0,094 \| 1200 \| 1,8 \| 218,82 \| \|  \|  \| 7,6 \| 72,4 \| 21,6 \| 6 \| 49,632 \| 38,07 \| 5,734 \| 0,47 \| 0,094 \| 1200 \| 2 \| 212,40 \| \|  \|  \| 7,6 \| 72,4 \| 21,6 \| 6 \| 49,632 \| 38,07 \| 5,734 \| 0,47 \| 0,094 \| 1200 \| 2,3 \| 204,22 \| \|  \|  \| 7,6 \| 72,4 \| 21,6 \| 6 \| 49,632 \| 38,07 \| 5,734 \| 0,47 \| 0,094 \| 1500 \| 1,8 \| 252,55 \| \|  \|  \| 7,6 \| 72,4 \| 21,6 \| 6 \| 49,632 \| 38,07 \| 5,734 \| 0,47 \| 0,094 \| 1500 \| 2 \| 250,48 \| \|  \|  \| 7,6 \| 72,4 \| 21,6 \| 6 \| 49,632 \| 38,07 \| 5,734 \| 0,47 \| 0,094 \| 1500 \| 2,3 \| 246,62 \| \| 18 \| Pine pruning \| 47,4 \| 82,2 \| 15,1 \| 2,7 \| 50,4987 \| 40,1849 \| 6,1299 \| 0,4865 \| 0 \| 600 \| 1,8 \| 56,72 \| \|  \|  \| 47,4 \| 82,2 \| 15,1 \| 2,7 \| 50,4987 \| 40,1849 \| 6,1299 \| 0,4865 \| 0 \| 600 \| 2 \| 53,20 \| \|  \|  \| 47,4 \| 82,2 \| 15,1 \| 2,7 \| 50,4987 \| 40,1849 \| 6,1299 \| 0,4865 \| 0 \| 600 \| 2,3 \| 49,34 \| \|  \|  \| 47,4 \| 82,2 \| 15,1 \| 2,7 \| 50,4987 \| 40,1849 \| 6,1299 \| 0,4865 \| 0 \| 900 \| 1,8 \| 113,65 \| \|  \|  \| 47,4 \| 82,2 \| 15,1 \| 2,7 \| 50,4987 \| 40,1849 \| 6,1299 \| 0,4865 \| 0 \| 900 \| 2 \| 107,91 \| \|  \|  \| 47,4 \| 82,2 \| 15,1 \| 2,7 \| 50,4987 \| 40,1849 \| 6,1299 \| 0,4865 \| 0 \| 900 \| 2,3 \| 101,17 \| \|  \|  \| 47,4 \| 82,2 \| 15,1 \| 2,7 \| 50,4987 \| 40,1849 \| 6,1299 \| 0,4865 \| 0 \| 1200 \| 1,8 \| 133,64 \| \|  \|  \| 47,4 \| 82,2 \| 15,1 \| 2,7 \| 50,4987 \| 40,1849 \| 6,1299 \| 0,4865 \| 0 \| 1200 \| 2 \| 130,48 \| \|  \|  \| 47,4 \| 82,2 \| 15,1 \| 2,7 \| 50,4987 \| 40,1849 \| 6,1299 \| 0,4865 \| 0 \| 1200 \| 2,3 \| 126,24 \| \|  \|  \| 47,4 \| 82,2 \| 15,1 \| 2,7 \| 50,4987 \| 40,1849 \| 6,1299 \| 0,4865 \| 0 \| 1500 \| 1,8 \| 152,80 \| \|  \|  \| 47,4 \| 82,2 \| 15,1 \| 2,7 \| 50,4987 \| 40,1849 \| 6,1299 \| 0,4865 \| 0 \| 1500 \| 2 \| 152,13 \| \|  \|  \| 47,4 \| 82,2 \| 15,1 \| 2,7 \| 50,4987 \| 40,1849 \| 6,1299 \| 0,4865 \| 0 \| 1500 \| 2,3 \| 150,39 \| \| 19 \| Pine sawdust \| 15,3 \| 83,1 \| 16,8 \| 0,1 \| 50,949 \| 42,8571 \| 5,994 \| 0,0999 \| 0 \| 600 \| 1,8 \| 83,30 \| \|  \|  \| 15,3 \| 83,1 \| 16,8 \| 0,1 \| 50,949 \| 42,8571 \| 5,994 \| 0,0999 \| 0 \| 600 \| 2 \| 77,46 \| \|  \|  \| 15,3 \| 83,1 \| 16,8 \| 0,1 \| 50,949 \| 42,8571 \| 5,994 \| 0,0999 \| 0 \| 600 \| 2,3 \| 71,12 \| \|  \|  \| 15,3 \| 83,1 \| 16,8 \| 0,1 \| 50,949 \| 42,8571 \| 5,994 \| 0,0999 \| 0 \| 900 \| 1,8 \| 173,68 \| \|  \|  \| 15,3 \| 83,1 \| 16,8 \| 0,1 \| 50,949 \| 42,8571 \| 5,994 \| 0,0999 \| 0 \| 900 \| 2 \| 163,84 \| \|  \|  \| 15,3 \| 83,1 \| 16,8 \| 0,1 \| 50,949 \| 42,8571 \| 5,994 \| 0,0999 \| 0 \| 900 \| 2,3 \| 152,54 \| \|  \|  \| 15,3 \| 83,1 \| 16,8 \| 0,1 \| 50,949 \| 42,8571 \| 5,994 \| 0,0999 \| 0 \| 1200 \| 1,8 \| 206,60 \| \|  \|  \| 15,3 \| 83,1 \| 16,8 \| 0,1 \| 50,949 \| 42,8571 \| 5,994 \| 0,0999 \| 0 \| 1200 \| 2 \| 200,88 \| \|  \|  \| 15,3 \| 83,1 \| 16,8 \| 0,1 \| 50,949 \| 42,8571 \| 5,994 \| 0,0999 \| 0 \| 1200 \| 2,3 \| 193,52 \| \|  \|  \| 15,3 \| 83,1 \| 16,8 \| 0,1 \| 50,949 \| 42,8571 \| 5,994 \| 0,0999 \| 0 \| 1500 \| 1,8 \| 238,14 \| \|  \|  \| 15,3 \| 83,1 \| 16,8 \| 0,1 \| 50,949 \| 42,8571 \| 5,994 \| 0,0999 \| 0 \| 1500 \| 2 \| 236,43 \| \|  \|  \| 15,3 \| 83,1 \| 16,8 \| 0,1 \| 50,949 \| 42,8571 \| 5,994 \| 0,0999 \| 0 \| 1500 \| 2,3 \| 233,07 \| \| 20 \| Poplar \| 6,8 \| 85,6 \| 12,3 \| 2,1 \| 50,5164 \| 40,8243 \| 5,9719 \| 0,5874 \| 0 \| 600 \| 1,8 \| 93,24 \| \|  \|  \| 6,8 \| 85,6 \| 12,3 \| 2,1 \| 50,5164 \| 40,8243 \| 5,9719 \| 0,5874 \| 0 \| 600 \| 2 \| 86,85 \| \|  \|  \| 6,8 \| 85,6 \| 12,3 \| 2,1 \| 50,5164 \| 40,8243 \| 5,9719 \| 0,5874 \| 0 \| 600 \| 2,3 \| 79,89 \| \|  \|  \| 6,8 \| 85,6 \| 12,3 \| 2,1 \| 50,5164 \| 40,8243 \| 5,9719 \| 0,5874 \| 0 \| 900 \| 1,8 \| 193,48 \| \|  \|  \| 6,8 \| 85,6 \| 12,3 \| 2,1 \| 50,5164 \| 40,8243 \| 5,9719 \| 0,5874 \| 0 \| 900 \| 2 \| 182,83 \| \|  \|  \| 6,8 \| 85,6 \| 12,3 \| 2,1 \| 50,5164 \| 40,8243 \| 5,9719 \| 0,5874 \| 0 \| 900 \| 2,3 \| 170,53 \| \|  \|  \| 6,8 \| 85,6 \| 12,3 \| 2,1 \| 50,5164 \| 40,8243 \| 5,9719 \| 0,5874 \| 0 \| 1200 \| 1,8 \| 229,26 \| \|  \|  \| 6,8 \| 85,6 \| 12,3 \| 2,1 \| 50,5164 \| 40,8243 \| 5,9719 \| 0,5874 \| 0 \| 1200 \| 2 \| 223,15 \| \|  \|  \| 6,8 \| 85,6 \| 12,3 \| 2,1 \| 50,5164 \| 40,8243 \| 5,9719 \| 0,5874 \| 0 \| 1200 \| 2,3 \| 215,21 \| \|  \|  \| 6,8 \| 85,6 \| 12,3 \| 2,1 \| 50,5164 \| 40,8243 \| 5,9719 \| 0,5874 \| 0 \| 1500 \| 1,8 \| 263,57 \| \|  \|  \| 6,8 \| 85,6 \| 12,3 \| 2,1 \| 50,5164 \| 40,8243 \| 5,9719 \| 0,5874 \| 0 \| 1500 \| 2 \| 261,87 \| \|  \|  \| 6,8 \| 85,6 \| 12,3 \| 2,1 \| 50,5164 \| 40,8243 \| 5,9719 \| 0,5874 \| 0 \| 1500 \| 2,3 \| 258,34 \| \| 21 \| Poplar bark \| 8,4 \| 80,3 \| 17,5 \| 2,2 \| 52,4208 \| 38,4354 \| 6,5526 \| 0,2934 \| 0,0978 \| 600 \| 1,8 \| 134,99 \| \|  \|  \| 8,4 \| 80,3 \| 17,5 \| 2,2 \| 52,4208 \| 38,4354 \| 6,5526 \| 0,2934 \| 0,0978 \| 600 \| 2 \| 129,93 \| \|  \|  \| 8,4 \| 80,3 \| 17,5 \| 2,2 \| 52,4208 \| 38,4354 \| 6,5526 \| 0,2934 \| 0,0978 \| 600 \| 2,3 \| 124,00 \| \|  \|  \| 8,4 \| 80,3 \| 17,5 \| 2,2 \| 52,4208 \| 38,4354 \| 6,5526 \| 0,2934 \| 0,0978 \| 900 \| 1,8 \| 238,65 \| \|  \|  \| 8,4 \| 80,3 \| 17,5 \| 2,2 \| 52,4208 \| 38,4354 \| 6,5526 \| 0,2934 \| 0,0978 \| 900 \| 2 \| 231,67 \| \|  \|  \| 8,4 \| 80,3 \| 17,5 \| 2,2 \| 52,4208 \| 38,4354 \| 6,5526 \| 0,2934 \| 0,0978 \| 900 \| 2,3 \| 222,33 \| \|  \|  \| 8,4 \| 80,3 \| 17,5 \| 2,2 \| 52,4208 \| 38,4354 \| 6,5526 \| 0,2934 \| 0,0978 \| 1200 \| 1,8 \| 271,14 \| \|  \|  \| 8,4 \| 80,3 \| 17,5 \| 2,2 \| 52,4208 \| 38,4354 \| 6,5526 \| 0,2934 \| 0,0978 \| 1200 \| 2 \| 268,69 \| \|  \|  \| 8,4 \| 80,3 \| 17,5 \| 2,2 \| 52,4208 \| 38,4354 \| 6,5526 \| 0,2934 \| 0,0978 \| 1200 \| 2,3 \| 264,00 \| \|  \|  \| 8,4 \| 80,3 \| 17,5 \| 2,2 \| 52,4208 \| 38,4354 \| 6,5526 \| 0,2934 \| 0,0978 \| 1500 \| 1,8 \| 302,41 \| \|  \|  \| 8,4 \| 80,3 \| 17,5 \| 2,2 \| 52,4208 \| 38,4354 \| 6,5526 \| 0,2934 \| 0,0978 \| 1500 \| 2 \| 304,07 \| \|  \|  \| 8,4 \| 80,3 \| 17,5 \| 2,2 \| 52,4208 \| 38,4354 \| 6,5526 \| 0,2934 \| 0,0978 \| 1500 \| 2,3 \| 303,78 \| \| 22 \| Sawdust \| 34,9 \| 84,6 \| 14,3 \| 1,1 \| 49,2522 \| 43,2193 \| 5,934 \| 0,4945 \| 0 \| 600 \| 1,8 \| 50,94 \| \|  \|  \| 34,9 \| 84,6 \| 14,3 \| 1,1 \| 49,2522 \| 43,2193 \| 5,934 \| 0,4945 \| 0 \| 600 \| 2 \| 46,19 \| \|  \|  \| 34,9 \| 84,6 \| 14,3 \| 1,1 \| 49,2522 \| 43,2193 \| 5,934 \| 0,4945 \| 0 \| 600 \| 2,3 \| 41,11 \| \|  \|  \| 34,9 \| 84,6 \| 14,3 \| 1,1 \| 49,2522 \| 43,2193 \| 5,934 \| 0,4945 \| 0 \| 900 \| 1,8 \| 117,02 \| \|  \|  \| 34,9 \| 84,6 \| 14,3 \| 1,1 \| 49,2522 \| 43,2193 \| 5,934 \| 0,4945 \| 0 \| 900 \| 2 \| 108,64 \| \|  \|  \| 34,9 \| 84,6 \| 14,3 \| 1,1 \| 49,2522 \| 43,2193 \| 5,934 \| 0,4945 \| 0 \| 900 \| 2,3 \| 99,36 \| \|  \|  \| 34,9 \| 84,6 \| 14,3 \| 1,1 \| 49,2522 \| 43,2193 \| 5,934 \| 0,4945 \| 0 \| 1200 \| 1,8 \| 143,23 \| \|  \|  \| 34,9 \| 84,6 \| 14,3 \| 1,1 \| 49,2522 \| 43,2193 \| 5,934 \| 0,4945 \| 0 \| 1200 \| 2 \| 137,86 \| \|  \|  \| 34,9 \| 84,6 \| 14,3 \| 1,1 \| 49,2522 \| 43,2193 \| 5,934 \| 0,4945 \| 0 \| 1200 \| 2,3 \| 131,45 \| \|  \|  \| 34,9 \| 84,6 \| 14,3 \| 1,1 \| 49,2522 \| 43,2193 \| 5,934 \| 0,4945 \| 0 \| 1500 \| 1,8 \| 168,41 \| \|  \|  \| 34,9 \| 84,6 \| 14,3 \| 1,1 \| 49,2522 \| 43,2193 \| 5,934 \| 0,4945 \| 0 \| 1500 \| 2 \| 166,07 \| \|  \|  \| 34,9 \| 84,6 \| 14,3 \| 1,1 \| 49,2522 \| 43,2193 \| 5,934 \| 0,4945 \| 0 \| 1500 \| 2,3 \| 162,61 \| \| 23 \| Spruce bark \| 8,4 \| 73,4 \| 23,4 \| 3,2 \| 51,8848 \| 38,72 \| 6,0016 \| 0,0968 \| 0,0968 \| 600 \| 1,8 \| 110,85 \| \|  \|  \| 8,4 \| 73,4 \| 23,4 \| 3,2 \| 51,8848 \| 38,72 \| 6,0016 \| 0,0968 \| 0,0968 \| 600 \| 2 \| 105,09 \| \|  \|  \| 8,4 \| 73,4 \| 23,4 \| 3,2 \| 51,8848 \| 38,72 \| 6,0016 \| 0,0968 \| 0,0968 \| 600 \| 2,3 \| 98,65 \| \|  \|  \| 8,4 \| 73,4 \| 23,4 \| 3,2 \| 51,8848 \| 38,72 \| 6,0016 \| 0,0968 \| 0,0968 \| 900 \| 1,8 \| 213,80 \| \|  \|  \| 8,4 \| 73,4 \| 23,4 \| 3,2 \| 51,8848 \| 38,72 \| 6,0016 \| 0,0968 \| 0,0968 \| 900 \| 2 \| 205,01 \| \|  \|  \| 8,4 \| 73,4 \| 23,4 \| 3,2 \| 51,8848 \| 38,72 \| 6,0016 \| 0,0968 \| 0,0968 \| 900 \| 2,3 \| 194,20 \| \|  \|  \| 8,4 \| 73,4 \| 23,4 \| 3,2 \| 51,8848 \| 38,72 \| 6,0016 \| 0,0968 \| 0,0968 \| 1200 \| 1,8 \| 247,12 \| \|  \|  \| 8,4 \| 73,4 \| 23,4 \| 3,2 \| 51,8848 \| 38,72 \| 6,0016 \| 0,0968 \| 0,0968 \| 1200 \| 2 \| 242,90 \| \|  \|  \| 8,4 \| 73,4 \| 23,4 \| 3,2 \| 51,8848 \| 38,72 \| 6,0016 \| 0,0968 \| 0,0968 \| 1200 \| 2,3 \| 236,60 \| \|  \|  \| 8,4 \| 73,4 \| 23,4 \| 3,2 \| 51,8848 \| 38,72 \| 6,0016 \| 0,0968 \| 0,0968 \| 1500 \| 1,8 \| 279,12 \| \|  \|  \| 8,4 \| 73,4 \| 23,4 \| 3,2 \| 51,8848 \| 38,72 \| 6,0016 \| 0,0968 \| 0,0968 \| 1500 \| 2 \| 279,17 \| \|  \|  \| 8,4 \| 73,4 \| 23,4 \| 3,2 \| 51,8848 \| 38,72 \| 6,0016 \| 0,0968 \| 0,0968 \| 1500 \| 2,3 \| 277,29 \| \| 24 \| Spruce wood \| 6,7 \| 81,2 \| 18,3 \| 0,5 \| 52,0385 \| 40,994 \| 6,0695 \| 0,2985 \| 0,0995 \| 600 \| 1,8 \| 110,19 \| \|  \|  \| 6,7 \| 81,2 \| 18,3 \| 0,5 \| 52,0385 \| 40,994 \| 6,0695 \| 0,2985 \| 0,0995 \| 600 \| 2 \| 104,21 \| \|  \|  \| 6,7 \| 81,2 \| 18,3 \| 0,5 \| 52,0385 \| 40,994 \| 6,0695 \| 0,2985 \| 0,0995 \| 600 \| 2,3 \| 97,56 \| \|  \|  \| 6,7 \| 81,2 \| 18,3 \| 0,5 \| 52,0385 \| 40,994 \| 6,0695 \| 0,2985 \| 0,0995 \| 900 \| 1,8 \| 213,71 \| \|  \|  \| 6,7 \| 81,2 \| 18,3 \| 0,5 \| 52,0385 \| 40,994 \| 6,0695 \| 0,2985 \| 0,0995 \| 900 \| 2 \| 204,34 \| \|  \|  \| 6,7 \| 81,2 \| 18,3 \| 0,5 \| 52,0385 \| 40,994 \| 6,0695 \| 0,2985 \| 0,0995 \| 900 \| 2,3 \| 192,98 \| \|  \|  \| 6,7 \| 81,2 \| 18,3 \| 0,5 \| 52,0385 \| 40,994 \| 6,0695 \| 0,2985 \| 0,0995 \| 1200 \| 1,8 \| 248,44 \| \|  \|  \| 6,7 \| 81,2 \| 18,3 \| 0,5 \| 52,0385 \| 40,994 \| 6,0695 \| 0,2985 \| 0,0995 \| 1200 \| 2 \| 243,71 \| \|  \|  \| 6,7 \| 81,2 \| 18,3 \| 0,5 \| 52,0385 \| 40,994 \| 6,0695 \| 0,2985 \| 0,0995 \| 1200 \| 2,3 \| 236,92 \| \|  \|  \| 6,7 \| 81,2 \| 18,3 \| 0,5 \| 52,0385 \| 40,994 \| 6,0695 \| 0,2985 \| 0,0995 \| 1500 \| 1,8 \| 281,73 \| \|  \|  \| 6,7 \| 81,2 \| 18,3 \| 0,5 \| 52,0385 \| 40,994 \| 6,0695 \| 0,2985 \| 0,0995 \| 1500 \| 2 \| 281,39 \| \|  \|  \| 6,7 \| 81,2 \| 18,3 \| 0,5 \| 52,0385 \| 40,994 \| 6,0695 \| 0,2985 \| 0,0995 \| 1500 \| 2,3 \| 279,11 \| \| 25 \| Tamarack bark \| 8,4 \| 69,5 \| 26,3 \| 4,2 \| 54,606 \| 30,656 \| 9,7716 \| 0,6706 \| 0,0958 \| 600 \| 1,8 \| 242,95 \| \|  \|  \| 8,4 \| 69,5 \| 26,3 \| 4,2 \| 54,606 \| 30,656 \| 9,7716 \| 0,6706 \| 0,0958 \| 600 \| 2 \| 254,60 \| \|  \|  \| 8,4 \| 69,5 \| 26,3 \| 4,2 \| 54,606 \| 30,656 \| 9,7716 \| 0,6706 \| 0,0958 \| 600 \| 2,3 \| 261,77 \| \|  \|  \| 8,4 \| 69,5 \| 26,3 \| 4,2 \| 54,606 \| 30,656 \| 9,7716 \| 0,6706 \| 0,0958 \| 900 \| 1,8 \| 357,40 \| \|  \|  \| 8,4 \| 69,5 \| 26,3 \| 4,2 \| 54,606 \| 30,656 \| 9,7716 \| 0,6706 \| 0,0958 \| 900 \| 2 \| 362,75 \| \|  \|  \| 8,4 \| 69,5 \| 26,3 \| 4,2 \| 54,606 \| 30,656 \| 9,7716 \| 0,6706 \| 0,0958 \| 900 \| 2,3 \| 366,10 \| \|  \|  \| 8,4 \| 69,5 \| 26,3 \| 4,2 \| 54,606 \| 30,656 \| 9,7716 \| 0,6706 \| 0,0958 \| 1200 \| 1,8 \| 388,38 \| \|  \|  \| 8,4 \| 69,5 \| 26,3 \| 4,2 \| 54,606 \| 30,656 \| 9,7716 \| 0,6706 \| 0,0958 \| 1200 \| 2 \| 395,76 \| \|  \|  \| 8,4 \| 69,5 \| 26,3 \| 4,2 \| 54,606 \| 30,656 \| 9,7716 \| 0,6706 \| 0,0958 \| 1200 \| 2,3 \| 401,78 \| \|  \|  \| 8,4 \| 69,5 \| 26,3 \| 4,2 \| 54,606 \| 30,656 \| 9,7716 \| 0,6706 \| 0,0958 \| 1500 \| 1,8 \| 418,75 \| \|  \|  \| 8,4 \| 69,5 \| 26,3 \| 4,2 \| 54,606 \| 30,656 \| 9,7716 \| 0,6706 \| 0,0958 \| 1500 \| 2 \| 428,30 \| \|  \|  \| 8,4 \| 69,5 \| 26,3 \| 4,2 \| 54,606 \| 30,656 \| 9,7716 \| 0,6706 \| 0,0958 \| 1500 \| 2,3 \| 436,79 \| \| 26 \| Willow \| 10,1 \| 82,5 \| 15,9 \| 1,6 \| 49,0032 \| 42,7056 \| 6,0024 \| 0,5904 \| 0,0984 \| 600 \| 1,8 \| 72,28 \| \|  \|  \| 10,1 \| 82,5 \| 15,9 \| 1,6 \| 49,0032 \| 42,7056 \| 6,0024 \| 0,5904 \| 0,0984 \| 600 \| 2 \| 65,75 \| \|  \|  \| 10,1 \| 82,5 \| 15,9 \| 1,6 \| 49,0032 \| 42,7056 \| 6,0024 \| 0,5904 \| 0,0984 \| 600 \| 2,3 \| 58,74 \| \|  \|  \| 10,1 \| 82,5 \| 15,9 \| 1,6 \| 49,0032 \| 42,7056 \| 6,0024 \| 0,5904 \| 0,0984 \| 900 \| 1,8 \| 163,49 \| \|  \|  \| 10,1 \| 82,5 \| 15,9 \| 1,6 \| 49,0032 \| 42,7056 \| 6,0024 \| 0,5904 \| 0,0984 \| 900 \| 2 \| 151,99 \| \|  \|  \| 10,1 \| 82,5 \| 15,9 \| 1,6 \| 49,0032 \| 42,7056 \| 6,0024 \| 0,5904 \| 0,0984 \| 900 \| 2,3 \| 139,24 \| \|  \|  \| 10,1 \| 82,5 \| 15,9 \| 1,6 \| 49,0032 \| 42,7056 \| 6,0024 \| 0,5904 \| 0,0984 \| 1200 \| 1,8 \| 199,61 \| \|  \|  \| 10,1 \| 82,5 \| 15,9 \| 1,6 \| 49,0032 \| 42,7056 \| 6,0024 \| 0,5904 \| 0,0984 \| 1200 \| 2 \| 192,29 \| \|  \|  \| 10,1 \| 82,5 \| 15,9 \| 1,6 \| 49,0032 \| 42,7056 \| 6,0024 \| 0,5904 \| 0,0984 \| 1200 \| 2,3 \| 183,50 \| \|  \|  \| 10,1 \| 82,5 \| 15,9 \| 1,6 \| 49,0032 \| 42,7056 \| 6,0024 \| 0,5904 \| 0,0984 \| 1500 \| 1,8 \| 234,32 \| \|  \|  \| 10,1 \| 82,5 \| 15,9 \| 1,6 \| 49,0032 \| 42,7056 \| 6,0024 \| 0,5904 \| 0,0984 \| 1500 \| 2 \| 231,19 \| \|  \|  \| 10,1 \| 82,5 \| 15,9 \| 1,6 \| 49,0032 \| 42,7056 \| 6,0024 \| 0,5904 \| 0,0984 \| 1500 \| 2,3 \| 226,49 \| \| 27 \| Wood \| 7,8 \| 84,1 \| 15,7 \| 0,2 \| 49,5008 \| 44,0118 \| 6,0878 \| 0,0998 \| 0,0998 \| 600 \| 1,8 \| 77,58 \| \|  \|  \| 7,8 \| 84,1 \| 15,7 \| 0,2 \| 49,5008 \| 44,0118 \| 6,0878 \| 0,0998 \| 0,0998 \| 600 \| 2 \| 70,94 \| \|  \|  \| 7,8 \| 84,1 \| 15,7 \| 0,2 \| 49,5008 \| 44,0118 \| 6,0878 \| 0,0998 \| 0,0998 \| 600 \| 2,3 \| 63,81 \| \|  \|  \| 7,8 \| 84,1 \| 15,7 \| 0,2 \| 49,5008 \| 44,0118 \| 6,0878 \| 0,0998 \| 0,0998 \| 900 \| 1,8 \| 171,27 \| \|  \|  \| 7,8 \| 84,1 \| 15,7 \| 0,2 \| 49,5008 \| 44,0118 \| 6,0878 \| 0,0998 \| 0,0998 \| 900 \| 2 \| 159,61 \| \|  \|  \| 7,8 \| 84,1 \| 15,7 \| 0,2 \| 49,5008 \| 44,0118 \| 6,0878 \| 0,0998 \| 0,0998 \| 900 \| 2,3 \| 146,63 \| \|  \|  \| 7,8 \| 84,1 \| 15,7 \| 0,2 \| 49,5008 \| 44,0118 \| 6,0878 \| 0,0998 \| 0,0998 \| 1200 \| 1,8 \| 208,40 \| \|  \|  \| 7,8 \| 84,1 \| 15,7 \| 0,2 \| 49,5008 \| 44,0118 \| 6,0878 \| 0,0998 \| 0,0998 \| 1200 \| 2 \| 201,06 \| \|  \|  \| 7,8 \| 84,1 \| 15,7 \| 0,2 \| 49,5008 \| 44,0118 \| 6,0878 \| 0,0998 \| 0,0998 \| 1200 \| 2,3 \| 192,18 \| \|  \|  \| 7,8 \| 84,1 \| 15,7 \| 0,2 \| 49,5008 \| 44,0118 \| 6,0878 \| 0,0998 \| 0,0998 \| 1500 \| 1,8 \| 244,04 \| \|  \|  \| 7,8 \| 84,1 \| 15,7 \| 0,2 \| 49,5008 \| 44,0118 \| 6,0878 \| 0,0998 \| 0,0998 \| 1500 \| 2 \| 241,02 \| \|  \|  \| 7,8 \| 84,1 \| 15,7 \| 0,2 \| 49,5008 \| 44,0118 \| 6,0878 \| 0,0998 \| 0,0998 \| 1500 \| 2,3 \| 236,36 \| \| 28 \| Wood residue \| 26,4 \| 78 \| 16,6 \| 5,4 \| 48,6244 \| 39,6374 \| 5,7706 \| 0,473 \| 0,0946 \| 600 \| 1,8 \| 57,11 \| \|  \|  \| 26,4 \| 78 \| 16,6 \| 5,4 \| 48,6244 \| 39,6374 \| 5,7706 \| 0,473 \| 0,0946 \| 600 \| 2 \| 51,73 \| \|  \|  \| 26,4 \| 78 \| 16,6 \| 5,4 \| 48,6244 \| 39,6374 \| 5,7706 \| 0,473 \| 0,0946 \| 600 \| 2,3 \| 45,97 \| \|  \|  \| 26,4 \| 78 \| 16,6 \| 5,4 \| 48,6244 \| 39,6374 \| 5,7706 \| 0,473 \| 0,0946 \| 900 \| 1,8 \| 133,10 \| \|  \|  \| 26,4 \| 78 \| 16,6 \| 5,4 \| 48,6244 \| 39,6374 \| 5,7706 \| 0,473 \| 0,0946 \| 900 \| 2 \| 123,73 \| \|  \|  \| 26,4 \| 78 \| 16,6 \| 5,4 \| 48,6244 \| 39,6374 \| 5,7706 \| 0,473 \| 0,0946 \| 900 \| 2,3 \| 113,30 \| \|  \|  \| 26,4 \| 78 \| 16,6 \| 5,4 \| 48,6244 \| 39,6374 \| 5,7706 \| 0,473 \| 0,0946 \| 1200 \| 1,8 \| 162,00 \| \|  \|  \| 26,4 \| 78 \| 16,6 \| 5,4 \| 48,6244 \| 39,6374 \| 5,7706 \| 0,473 \| 0,0946 \| 1200 \| 2 \| 156,07 \| \|  \|  \| 26,4 \| 78 \| 16,6 \| 5,4 \| 48,6244 \| 39,6374 \| 5,7706 \| 0,473 \| 0,0946 \| 1200 \| 2,3 \| 148,90 \| \|  \|  \| 26,4 \| 78 \| 16,6 \| 5,4 \| 48,6244 \| 39,6374 \| 5,7706 \| 0,473 \| 0,0946 \| 1500 \| 1,8 \| 189,80 \| \|  \|  \| 26,4 \| 78 \| 16,6 \| 5,4 \| 48,6244 \| 39,6374 \| 5,7706 \| 0,473 \| 0,0946 \| 1500 \| 2 \| 187,30 \| \|  \|  \| 26,4 \| 78 \| 16,6 \| 5,4 \| 48,6244 \| 39,6374 \| 5,7706 \| 0,473 \| 0,0946 \| 1500 \| 2,3 \| 183,49 \| \| 29 \| Arundo grass \| 42 \| 80,2 \| 16,4 \| 3,4 \| 47,0442 \| 42,987 \| 5,8926 \| 0,5796 \| 0,0966 \| 600 \| 1,8 \| 32,16 \| \|  \|  \| 42 \| 80,2 \| 16,4 \| 3,4 \| 47,0442 \| 42,987 \| 5,8926 \| 0,5796 \| 0,0966 \| 600 \| 2 \| 27,69 \| \|  \|  \| 42 \| 80,2 \| 16,4 \| 3,4 \| 47,0442 \| 42,987 \| 5,8926 \| 0,5796 \| 0,0966 \| 600 \| 2,3 \| 22,95 \| \|  \|  \| 42 \| 80,2 \| 16,4 \| 3,4 \| 47,0442 \| 42,987 \| 5,8926 \| 0,5796 \| 0,0966 \| 900 \| 1,8 \| 87,10 \| \|  \|  \| 42 \| 80,2 \| 16,4 \| 3,4 \| 47,0442 \| 42,987 \| 5,8926 \| 0,5796 \| 0,0966 \| 900 \| 2 \| 78,97 \| \|  \|  \| 42 \| 80,2 \| 16,4 \| 3,4 \| 47,0442 \| 42,987 \| 5,8926 \| 0,5796 \| 0,0966 \| 900 \| 2,3 \| 70,21 \| \|  \|  \| 42 \| 80,2 \| 16,4 \| 3,4 \| 47,0442 \| 42,987 \| 5,8926 \| 0,5796 \| 0,0966 \| 1200 \| 1,8 \| 111,12 \| \|  \|  \| 42 \| 80,2 \| 16,4 \| 3,4 \| 47,0442 \| 42,987 \| 5,8926 \| 0,5796 \| 0,0966 \| 1200 \| 2 \| 105,53 \| \|  \|  \| 42 \| 80,2 \| 16,4 \| 3,4 \| 47,0442 \| 42,987 \| 5,8926 \| 0,5796 \| 0,0966 \| 1200 \| 2,3 \| 99,18 \| \|  \|  \| 42 \| 80,2 \| 16,4 \| 3,4 \| 47,0442 \| 42,987 \| 5,8926 \| 0,5796 \| 0,0966 \| 1500 \| 1,8 \| 134,34 \| \|  \|  \| 42 \| 80,2 \| 16,4 \| 3,4 \| 47,0442 \| 42,987 \| 5,8926 \| 0,5796 \| 0,0966 \| 1500 \| 2 \| 131,34 \| \|  \|  \| 42 \| 80,2 \| 16,4 \| 3,4 \| 47,0442 \| 42,987 \| 5,8926 \| 0,5796 \| 0,0966 \| 1500 \| 2,3 \| 127,50 \| \| 30 \| Bamboo whole \| 13 \| 81,6 \| 17,5 \| 0,9 \| 51,532 \| 42,1175 \| 5,0541 \| 0,3964 \| 0 \| 600 \| 1,8 \| 61,54 \| \|  \|  \| 13 \| 81,6 \| 17,5 \| 0,9 \| 51,532 \| 42,1175 \| 5,0541 \| 0,3964 \| 0 \| 600 \| 2 \| 55,17 \| \|  \|  \| 13 \| 81,6 \| 17,5 \| 0,9 \| 51,532 \| 42,1175 \| 5,0541 \| 0,3964 \| 0 \| 600 \| 2,3 \| 48,34 \| \|  \|  \| 13 \| 81,6 \| 17,5 \| 0,9 \| 51,532 \| 42,1175 \| 5,0541 \| 0,3964 \| 0 \| 900 \| 1,8 \| 155,24 \| \|  \|  \| 13 \| 81,6 \| 17,5 \| 0,9 \| 51,532 \| 42,1175 \| 5,0541 \| 0,3964 \| 0 \| 900 \| 2 \| 144,14 \| \|  \|  \| 13 \| 81,6 \| 17,5 \| 0,9 \| 51,532 \| 42,1175 \| 5,0541 \| 0,3964 \| 0 \| 900 \| 2,3 \| 131,75 \| \|  \|  \| 13 \| 81,6 \| 17,5 \| 0,9 \| 51,532 \| 42,1175 \| 5,0541 \| 0,3964 \| 0 \| 1200 \| 1,8 \| 188,89 \| \|  \|  \| 13 \| 81,6 \| 17,5 \| 0,9 \| 51,532 \| 42,1175 \| 5,0541 \| 0,3964 \| 0 \| 1200 \| 2 \| 181,88 \| \|  \|  \| 13 \| 81,6 \| 17,5 \| 0,9 \| 51,532 \| 42,1175 \| 5,0541 \| 0,3964 \| 0 \| 1200 \| 2,3 \| 173,37 \| \|  \|  \| 13 \| 81,6 \| 17,5 \| 0,9 \| 51,532 \| 42,1175 \| 5,0541 \| 0,3964 \| 0 \| 1500 \| 1,8 \| 221,28 \| \|  \|  \| 13 \| 81,6 \| 17,5 \| 0,9 \| 51,532 \| 42,1175 \| 5,0541 \| 0,3964 \| 0 \| 1500 \| 2 \| 218,34 \| \|  \|  \| 13 \| 81,6 \| 17,5 \| 0,9 \| 51,532 \| 42,1175 \| 5,0541 \| 0,3964 \| 0 \| 1500 \| 2,3 \| 213,81 \| \| 31 \| Bana grass \| 4,5 \| 73,6 \| 16,6 \| 9,8 \| 45,1902 \| 38,6958 \| 5,412 \| 0,8118 \| 0,0902 \| 600 \| 1,8 \| 22,19 \| \|  \|  \| 4,5 \| 73,6 \| 16,6 \| 9,8 \| 45,1902 \| 38,6958 \| 5,412 \| 0,8118 \| 0,0902 \| 600 \| 2 \| 14,37 \| \|  \|  \| 4,5 \| 73,6 \| 16,6 \| 9,8 \| 45,1902 \| 38,6958 \| 5,412 \| 0,8118 \| 0,0902 \| 600 \| 2,3 \| 6,15 \| \|  \|  \| 4,5 \| 73,6 \| 16,6 \| 9,8 \| 45,1902 \| 38,6958 \| 5,412 \| 0,8118 \| 0,0902 \| 900 \| 1,8 \| 108,65 \| \|  \|  \| 4,5 \| 73,6 \| 16,6 \| 9,8 \| 45,1902 \| 38,6958 \| 5,412 \| 0,8118 \| 0,0902 \| 900 \| 2 \| 94,33 \| \|  \|  \| 4,5 \| 73,6 \| 16,6 \| 9,8 \| 45,1902 \| 38,6958 \| 5,412 \| 0,8118 \| 0,0902 \| 900 \| 2,3 \| 79,16 \| \|  \|  \| 4,5 \| 73,6 \| 16,6 \| 9,8 \| 45,1902 \| 38,6958 \| 5,412 \| 0,8118 \| 0,0902 \| 1200 \| 1,8 \| 147,67 \| \|  \|  \| 4,5 \| 73,6 \| 16,6 \| 9,8 \| 45,1902 \| 38,6958 \| 5,412 \| 0,8118 \| 0,0902 \| 1200 \| 2 \| 137,31 \| \|  \|  \| 4,5 \| 73,6 \| 16,6 \| 9,8 \| 45,1902 \| 38,6958 \| 5,412 \| 0,8118 \| 0,0902 \| 1200 \| 2,3 \| 125,94 \| \|  \|  \| 4,5 \| 73,6 \| 16,6 \| 9,8 \| 45,1902 \| 38,6958 \| 5,412 \| 0,8118 \| 0,0902 \| 1500 \| 1,8 \| 185,71 \| \|  \|  \| 4,5 \| 73,6 \| 16,6 \| 9,8 \| 45,1902 \| 38,6958 \| 5,412 \| 0,8118 \| 0,0902 \| 1500 \| 2 \| 179,41 \| \|  \|  \| 4,5 \| 73,6 \| 16,6 \| 9,8 \| 45,1902 \| 38,6958 \| 5,412 \| 0,8118 \| 0,0902 \| 1500 \| 2,3 \| 171,96 \| \| 32 \| Buffalo gourd grass \| 10 \| 81,6 \| 13,7 \| 4,7 \| 43,9333 \| 42,4085 \| 6,1945 \| 2,4778 \| 0,2859 \| 600 \| 1,8 \| 35,14 \| \|  \|  \| 10 \| 81,6 \| 13,7 \| 4,7 \| 43,9333 \| 42,4085 \| 6,1945 \| 2,4778 \| 0,2859 \| 600 \| 2 \| 27,90 \| \|  \|  \| 10 \| 81,6 \| 13,7 \| 4,7 \| 43,9333 \| 42,4085 \| 6,1945 \| 2,4778 \| 0,2859 \| 600 \| 2,3 \| 20,26 \| \|  \|  \| 10 \| 81,6 \| 13,7 \| 4,7 \| 43,9333 \| 42,4085 \| 6,1945 \| 2,4778 \| 0,2859 \| 900 \| 1,8 \| 112,46 \| \|  \|  \| 10 \| 81,6 \| 13,7 \| 4,7 \| 43,9333 \| 42,4085 \| 6,1945 \| 2,4778 \| 0,2859 \| 900 \| 2 \| 99,13 \| \|  \|  \| 10 \| 81,6 \| 13,7 \| 4,7 \| 43,9333 \| 42,4085 \| 6,1945 \| 2,4778 \| 0,2859 \| 900 \| 2,3 \| 85,01 \| \|  \|  \| 10 \| 81,6 \| 13,7 \| 4,7 \| 43,9333 \| 42,4085 \| 6,1945 \| 2,4778 \| 0,2859 \| 1200 \| 1,8 \| 151,02 \| \|  \|  \| 10 \| 81,6 \| 13,7 \| 4,7 \| 43,9333 \| 42,4085 \| 6,1945 \| 2,4778 \| 0,2859 \| 1200 \| 2 \| 141,42 \| \|  \|  \| 10 \| 81,6 \| 13,7 \| 4,7 \| 43,9333 \| 42,4085 \| 6,1945 \| 2,4778 \| 0,2859 \| 1200 \| 2,3 \| 130,88 \| \|  \|  \| 10 \| 81,6 \| 13,7 \| 4,7 \| 43,9333 \| 42,4085 \| 6,1945 \| 2,4778 \| 0,2859 \| 1500 \| 1,8 \| 188,52 \| \|  \|  \| 10 \| 81,6 \| 13,7 \| 4,7 \| 43,9333 \| 42,4085 \| 6,1945 \| 2,4778 \| 0,2859 \| 1500 \| 2 \| 182,78 \| \|  \|  \| 10 \| 81,6 \| 13,7 \| 4,7 \| 43,9333 \| 42,4085 \| 6,1945 \| 2,4778 \| 0,2859 \| 1500 \| 2,3 \| 175,97 \| \| 33 \| Kenaf grass \| 7,5 \| 79,4 \| 17 \| 3,6 \| 46,6576 \| 42,898 \| 5,784 \| 0,964 \| 0,0964 \| 600 \| 1,8 \| 44,21 \| \|  \|  \| 7,5 \| 79,4 \| 17 \| 3,6 \| 46,6576 \| 42,898 \| 5,784 \| 0,964 \| 0,0964 \| 600 \| 2 \| 36,97 \| \|  \|  \| 7,5 \| 79,4 \| 17 \| 3,6 \| 46,6576 \| 42,898 \| 5,784 \| 0,964 \| 0,0964 \| 600 \| 2,3 \| 29,32 \| \|  \|  \| 7,5 \| 79,4 \| 17 \| 3,6 \| 46,6576 \| 42,898 \| 5,784 \| 0,964 \| 0,0964 \| 900 \| 1,8 \| 130,46 \| \|  \|  \| 7,5 \| 79,4 \| 17 \| 3,6 \| 46,6576 \| 42,898 \| 5,784 \| 0,964 \| 0,0964 \| 900 \| 2 \| 117,24 \| \|  \|  \| 7,5 \| 79,4 \| 17 \| 3,6 \| 46,6576 \| 42,898 \| 5,784 \| 0,964 \| 0,0964 \| 900 \| 2,3 \| 103,07 \| \|  \|  \| 7,5 \| 79,4 \| 17 \| 3,6 \| 46,6576 \| 42,898 \| 5,784 \| 0,964 \| 0,0964 \| 1200 \| 1,8 \| 168,96 \| \|  \|  \| 7,5 \| 79,4 \| 17 \| 3,6 \| 46,6576 \| 42,898 \| 5,784 \| 0,964 \| 0,0964 \| 1200 \| 2 \| 159,72 \| \|  \|  \| 7,5 \| 79,4 \| 17 \| 3,6 \| 46,6576 \| 42,898 \| 5,784 \| 0,964 \| 0,0964 \| 1200 \| 2,3 \| 149,34 \| \|  \|  \| 7,5 \| 79,4 \| 17 \| 3,6 \| 46,6576 \| 42,898 \| 5,784 \| 0,964 \| 0,0964 \| 1500 \| 1,8 \| 206,24 \| \|  \|  \| 7,5 \| 79,4 \| 17 \| 3,6 \| 46,6576 \| 42,898 \| 5,784 \| 0,964 \| 0,0964 \| 1500 \| 2 \| 201,09 \| \|  \|  \| 7,5 \| 79,4 \| 17 \| 3,6 \| 46,6576 \| 42,898 \| 5,784 \| 0,964 \| 0,0964 \| 1500 \| 2,3 \| 194,64 \| \| 34 \| Miscanthus grass \| 11,4 \| 81,2 \| 15,8 \| 3 \| 47,724 \| 42,874 \| 5,82 \| 0,388 \| 0,194 \| 600 \| 1,8 \| 53,23 \| \|  \|  \| 11,4 \| 81,2 \| 15,8 \| 3 \| 47,724 \| 42,874 \| 5,82 \| 0,388 \| 0,194 \| 600 \| 2 \| 46,48 \| \|  \|  \| 11,4 \| 81,2 \| 15,8 \| 3 \| 47,724 \| 42,874 \| 5,82 \| 0,388 \| 0,194 \| 600 \| 2,3 \| 39,31 \| \|  \|  \| 11,4 \| 81,2 \| 15,8 \| 3 \| 47,724 \| 42,874 \| 5,82 \| 0,388 \| 0,194 \| 900 \| 1,8 \| 139,02 \| \|  \|  \| 11,4 \| 81,2 \| 15,8 \| 3 \| 47,724 \| 42,874 \| 5,82 \| 0,388 \| 0,194 \| 900 \| 2 \| 126,83 \| \|  \|  \| 11,4 \| 81,2 \| 15,8 \| 3 \| 47,724 \| 42,874 \| 5,82 \| 0,388 \| 0,194 \| 900 \| 2,3 \| 113,61 \| \|  \|  \| 11,4 \| 81,2 \| 15,8 \| 3 \| 47,724 \| 42,874 \| 5,82 \| 0,388 \| 0,194 \| 1200 \| 1,8 \| 175,34 \| \|  \|  \| 11,4 \| 81,2 \| 15,8 \| 3 \| 47,724 \| 42,874 \| 5,82 \| 0,388 \| 0,194 \| 1200 \| 2 \| 167,08 \| \|  \|  \| 11,4 \| 81,2 \| 15,8 \| 3 \| 47,724 \| 42,874 \| 5,82 \| 0,388 \| 0,194 \| 1200 \| 2,3 \| 157,59 \| \|  \|  \| 11,4 \| 81,2 \| 15,8 \| 3 \| 47,724 \| 42,874 \| 5,82 \| 0,388 \| 0,194 \| 1500 \| 1,8 \| 210,39 \| \|  \|  \| 11,4 \| 81,2 \| 15,8 \| 3 \| 47,724 \| 42,874 \| 5,82 \| 0,388 \| 0,194 \| 1500 \| 2 \| 206,15 \| \|  \|  \| 11,4 \| 81,2 \| 15,8 \| 3 \| 47,724 \| 42,874 \| 5,82 \| 0,388 \| 0,194 \| 1500 \| 2,3 \| 200,55 \| \| 35 \| Reed canary grass \| 7,7 \| 73,4 \| 17,7 \| 8,9 \| 45,0034 \| 38,8997 \| 5,7393 \| 1,3665 \| 0,0911 \| 600 \| 1,8 \| 39,34 \| \|  \|  \| 7,7 \| 73,4 \| 17,7 \| 8,9 \| 45,0034 \| 38,8997 \| 5,7393 \| 1,3665 \| 0,0911 \| 600 \| 2 \| 32,02 \| \|  \|  \| 7,7 \| 73,4 \| 17,7 \| 8,9 \| 45,0034 \| 38,8997 \| 5,7393 \| 1,3665 \| 0,0911 \| 600 \| 2,3 \| 24,29 \| \|  \|  \| 7,7 \| 73,4 \| 17,7 \| 8,9 \| 45,0034 \| 38,8997 \| 5,7393 \| 1,3665 \| 0,0911 \| 900 \| 1,8 \| 124,84 \| \|  \|  \| 7,7 \| 73,4 \| 17,7 \| 8,9 \| 45,0034 \| 38,8997 \| 5,7393 \| 1,3665 \| 0,0911 \| 900 \| 2 \| 111,51 \| \|  \|  \| 7,7 \| 73,4 \| 17,7 \| 8,9 \| 45,0034 \| 38,8997 \| 5,7393 \| 1,3665 \| 0,0911 \| 900 \| 2,3 \| 97,28 \| \|  \|  \| 7,7 \| 73,4 \| 17,7 \| 8,9 \| 45,0034 \| 38,8997 \| 5,7393 \| 1,3665 \| 0,0911 \| 1200 \| 1,8 \| 162,75 \| \|  \|  \| 7,7 \| 73,4 \| 17,7 \| 8,9 \| 45,0034 \| 38,8997 \| 5,7393 \| 1,3665 \| 0,0911 \| 1200 \| 2 \| 153,37 \| \|  \|  \| 7,7 \| 73,4 \| 17,7 \| 8,9 \| 45,0034 \| 38,8997 \| 5,7393 \| 1,3665 \| 0,0911 \| 1200 \| 2,3 \| 142,89 \| \|  \|  \| 7,7 \| 73,4 \| 17,7 \| 8,9 \| 45,0034 \| 38,8997 \| 5,7393 \| 1,3665 \| 0,0911 \| 1500 \| 1,8 \| 199,54 \| \|  \|  \| 7,7 \| 73,4 \| 17,7 \| 8,9 \| 45,0034 \| 38,8997 \| 5,7393 \| 1,3665 \| 0,0911 \| 1500 \| 2 \| 194,20 \| \|  \|  \| 7,7 \| 73,4 \| 17,7 \| 8,9 \| 45,0034 \| 38,8997 \| 5,7393 \| 1,3665 \| 0,0911 \| 1500 \| 2,3 \| 187,61 \| \| 36 \| Sorghastrum grass \| 11,3 \| 81,6 \| 14,2 \| 4,2 \| 47,3252 \| 42,152 \| 6,0354 \| 0,2874 \| 0 \| 600 \| 1,8 \| 58,68 \| \|  \|  \| 11,3 \| 81,6 \| 14,2 \| 4,2 \| 47,3252 \| 42,152 \| 6,0354 \| 0,2874 \| 0 \| 600 \| 2 \| 51,99 \| \|  \|  \| 11,3 \| 81,6 \| 14,2 \| 4,2 \| 47,3252 \| 42,152 \| 6,0354 \| 0,2874 \| 0 \| 600 \| 2,3 \| 44,86 \| \|  \|  \| 11,3 \| 81,6 \| 14,2 \| 4,2 \| 47,3252 \| 42,152 \| 6,0354 \| 0,2874 \| 0 \| 900 \| 1,8 \| 144,78 \| \|  \|  \| 11,3 \| 81,6 \| 14,2 \| 4,2 \| 47,3252 \| 42,152 \| 6,0354 \| 0,2874 \| 0 \| 900 \| 2 \| 132,75 \| \|  \|  \| 11,3 \| 81,6 \| 14,2 \| 4,2 \| 47,3252 \| 42,152 \| 6,0354 \| 0,2874 \| 0 \| 900 \| 2,3 \| 119,66 \| \|  \|  \| 11,3 \| 81,6 \| 14,2 \| 4,2 \| 47,3252 \| 42,152 \| 6,0354 \| 0,2874 \| 0 \| 1200 \| 1,8 \| 181,07 \| \|  \|  \| 11,3 \| 81,6 \| 14,2 \| 4,2 \| 47,3252 \| 42,152 \| 6,0354 \| 0,2874 \| 0 \| 1200 \| 2 \| 173,02 \| \|  \|  \| 11,3 \| 81,6 \| 14,2 \| 4,2 \| 47,3252 \| 42,152 \| 6,0354 \| 0,2874 \| 0 \| 1200 \| 2,3 \| 163,70 \| \|  \|  \| 11,3 \| 81,6 \| 14,2 \| 4,2 \| 47,3252 \| 42,152 \| 6,0354 \| 0,2874 \| 0 \| 1500 \| 1,8 \| 216,05 \| \|  \|  \| 11,3 \| 81,6 \| 14,2 \| 4,2 \| 47,3252 \| 42,152 \| 6,0354 \| 0,2874 \| 0 \| 1500 \| 2 \| 212,05 \| \|  \|  \| 11,3 \| 81,6 \| 14,2 \| 4,2 \| 47,3252 \| 42,152 \| 6,0354 \| 0,2874 \| 0 \| 1500 \| 2,3 \| 206,64 \| \| 37 \| Sweet sorghum grass \| 7 \| 77,2 \| 18,1 \| 4,7 \| 47,3641 \| 41,6461 \| 5,8133 \| 0,3812 \| 0,0953 \| 600 \| 1,8 \| 55,71 \| \|  \|  \| 7 \| 77,2 \| 18,1 \| 4,7 \| 47,3641 \| 41,6461 \| 5,8133 \| 0,3812 \| 0,0953 \| 600 \| 2 \| 48,61 \| \|  \|  \| 7 \| 77,2 \| 18,1 \| 4,7 \| 47,3641 \| 41,6461 \| 5,8133 \| 0,3812 \| 0,0953 \| 600 \| 2,3 \| 41,08 \| \|  \|  \| 7 \| 77,2 \| 18,1 \| 4,7 \| 47,3641 \| 41,6461 \| 5,8133 \| 0,3812 \| 0,0953 \| 900 \| 1,8 \| 146,06 \| \|  \|  \| 7 \| 77,2 \| 18,1 \| 4,7 \| 47,3641 \| 41,6461 \| 5,8133 \| 0,3812 \| 0,0953 \| 900 \| 2 \| 133,28 \| \|  \|  \| 7 \| 77,2 \| 18,1 \| 4,7 \| 47,3641 \| 41,6461 \| 5,8133 \| 0,3812 \| 0,0953 \| 900 \| 2,3 \| 119,42 \| \|  \|  \| 7 \| 77,2 \| 18,1 \| 4,7 \| 47,3641 \| 41,6461 \| 5,8133 \| 0,3812 \| 0,0953 \| 1200 \| 1,8 \| 183,97 \| \|  \|  \| 7 \| 77,2 \| 18,1 \| 4,7 \| 47,3641 \| 41,6461 \| 5,8133 \| 0,3812 \| 0,0953 \| 1200 \| 2 \| 175,32 \| \|  \|  \| 7 \| 77,2 \| 18,1 \| 4,7 \| 47,3641 \| 41,6461 \| 5,8133 \| 0,3812 \| 0,0953 \| 1200 \| 2,3 \| 165,38 \| \|  \|  \| 7 \| 77,2 \| 18,1 \| 4,7 \| 47,3641 \| 41,6461 \| 5,8133 \| 0,3812 \| 0,0953 \| 1500 \| 1,8 \| 220,56 \| \|  \|  \| 7 \| 77,2 \| 18,1 \| 4,7 \| 47,3641 \| 41,6461 \| 5,8133 \| 0,3812 \| 0,0953 \| 1500 \| 2 \| 216,13 \| \|  \|  \| 7 \| 77,2 \| 18,1 \| 4,7 \| 47,3641 \| 41,6461 \| 5,8133 \| 0,3812 \| 0,0953 \| 1500 \| 2,3 \| 210,26 \| \| 38 \| Switchgrass \| 11,9 \| 80,4 \| 14,5 \| 5,1 \| 47,1653 \| 41,1866 \| 5,7889 \| 0,6643 \| 0,0949 \| 600 \| 1,8 \| 51,67 \| \|  \|  \| 11,9 \| 80,4 \| 14,5 \| 5,1 \| 47,1653 \| 41,1866 \| 5,7889 \| 0,6643 \| 0,0949 \| 600 \| 2 \| 44,91 \| \|  \|  \| 11,9 \| 80,4 \| 14,5 \| 5,1 \| 47,1653 \| 41,1866 \| 5,7889 \| 0,6643 \| 0,0949 \| 600 \| 2,3 \| 37,75 \| \|  \|  \| 11,9 \| 80,4 \| 14,5 \| 5,1 \| 47,1653 \| 41,1866 \| 5,7889 \| 0,6643 \| 0,0949 \| 900 \| 1,8 \| 137,16 \| \|  \|  \| 11,9 \| 80,4 \| 14,5 \| 5,1 \| 47,1653 \| 41,1866 \| 5,7889 \| 0,6643 \| 0,0949 \| 900 \| 2 \| 125,00 \| \|  \|  \| 11,9 \| 80,4 \| 14,5 \| 5,1 \| 47,1653 \| 41,1866 \| 5,7889 \| 0,6643 \| 0,0949 \| 900 \| 2,3 \| 111,82 \| \|  \|  \| 11,9 \| 80,4 \| 14,5 \| 5,1 \| 47,1653 \| 41,1866 \| 5,7889 \| 0,6643 \| 0,0949 \| 1200 \| 1,8 \| 173,04 \| \|  \|  \| 11,9 \| 80,4 \| 14,5 \| 5,1 \| 47,1653 \| 41,1866 \| 5,7889 \| 0,6643 \| 0,0949 \| 1200 \| 2 \| 164,79 \| \|  \|  \| 11,9 \| 80,4 \| 14,5 \| 5,1 \| 47,1653 \| 41,1866 \| 5,7889 \| 0,6643 \| 0,0949 \| 1200 \| 2,3 \| 155,32 \| \|  \|  \| 11,9 \| 80,4 \| 14,5 \| 5,1 \| 47,1653 \| 41,1866 \| 5,7889 \| 0,6643 \| 0,0949 \| 1500 \| 1,8 \| 207,69 \| \|  \|  \| 11,9 \| 80,4 \| 14,5 \| 5,1 \| 47,1653 \| 41,1866 \| 5,7889 \| 0,6643 \| 0,0949 \| 1500 \| 2 \| 203,43 \| \|  \|  \| 11,9 \| 80,4 \| 14,5 \| 5,1 \| 47,1653 \| 41,1866 \| 5,7889 \| 0,6643 \| 0,0949 \| 1500 \| 2,3 \| 197,81 \| \| 39 \| Alfalfa straw \| 9,3 \| 78,9 \| 15,8 \| 5,3 \| 47,2553 \| 38,6376 \| 5,9661 \| 2,6516 \| 0,1894 \| 600 \| 1,8 \| 247,36 \| \|  \|  \| 9,3 \| 78,9 \| 15,8 \| 5,3 \| 47,2553 \| 38,6376 \| 5,9661 \| 2,6516 \| 0,1894 \| 600 \| 2 \| 240,29 \| \|  \|  \| 9,3 \| 78,9 \| 15,8 \| 5,3 \| 47,2553 \| 38,6376 \| 5,9661 \| 2,6516 \| 0,1894 \| 600 \| 2,3 \| 232,81 \| \|  \|  \| 9,3 \| 78,9 \| 15,8 \| 5,3 \| 47,2553 \| 38,6376 \| 5,9661 \| 2,6516 \| 0,1894 \| 900 \| 1,8 \| 340,20 \| \|  \|  \| 9,3 \| 78,9 \| 15,8 \| 5,3 \| 47,2553 \| 38,6376 \| 5,9661 \| 2,6516 \| 0,1894 \| 900 \| 2 \| 327,66 \| \|  \|  \| 9,3 \| 78,9 \| 15,8 \| 5,3 \| 47,2553 \| 38,6376 \| 5,9661 \| 2,6516 \| 0,1894 \| 900 \| 2,3 \| 314,07 \| \|  \|  \| 9,3 \| 78,9 \| 15,8 \| 5,3 \| 47,2553 \| 38,6376 \| 5,9661 \| 2,6516 \| 0,1894 \| 1200 \| 1,8 \| 377,26 \| \|  \|  \| 9,3 \| 78,9 \| 15,8 \| 5,3 \| 47,2553 \| 38,6376 \| 5,9661 \| 2,6516 \| 0,1894 \| 1200 \| 2 \| 368,77 \| \|  \|  \| 9,3 \| 78,9 \| 15,8 \| 5,3 \| 47,2553 \| 38,6376 \| 5,9661 \| 2,6516 \| 0,1894 \| 1200 \| 2,3 \| 359,03 \| \|  \|  \| 9,3 \| 78,9 \| 15,8 \| 5,3 \| 47,2553 \| 38,6376 \| 5,9661 \| 2,6516 \| 0,1894 \| 1500 \| 1,8 \| 413,08 \| \|  \|  \| 9,3 \| 78,9 \| 15,8 \| 5,3 \| 47,2553 \| 38,6376 \| 5,9661 \| 2,6516 \| 0,1894 \| 1500 \| 2 \| 408,74 \| \|  \|  \| 9,3 \| 78,9 \| 15,8 \| 5,3 \| 47,2553 \| 38,6376 \| 5,9661 \| 2,6516 \| 0,1894 \| 1500 \| 2,3 \| 402,98 \| \| 40 \| Barley straw \| 11,5 \| 76,2 \| 18,5 \| 5,3 \| 46,7818 \| 41,2892 \| 5,8714 \| 0,6629 \| 0,0947 \| 600 \| 1,8 \| 50,97 \| \|  \|  \| 11,5 \| 76,2 \| 18,5 \| 5,3 \| 46,7818 \| 41,2892 \| 5,8714 \| 0,6629 \| 0,0947 \| 600 \| 2 \| 44,18 \| \|  \|  \| 11,5 \| 76,2 \| 18,5 \| 5,3 \| 46,7818 \| 41,2892 \| 5,8714 \| 0,6629 \| 0,0947 \| 600 \| 2,3 \| 36,98 \| \|  \|  \| 11,5 \| 76,2 \| 18,5 \| 5,3 \| 46,7818 \| 41,2892 \| 5,8714 \| 0,6629 \| 0,0947 \| 900 \| 1,8 \| 135,83 \| \|  \|  \| 11,5 \| 76,2 \| 18,5 \| 5,3 \| 46,7818 \| 41,2892 \| 5,8714 \| 0,6629 \| 0,0947 \| 900 \| 2 \| 123,56 \| \|  \|  \| 11,5 \| 76,2 \| 18,5 \| 5,3 \| 46,7818 \| 41,2892 \| 5,8714 \| 0,6629 \| 0,0947 \| 900 \| 2,3 \| 110,30 \| \|  \|  \| 11,5 \| 76,2 \| 18,5 \| 5,3 \| 46,7818 \| 41,2892 \| 5,8714 \| 0,6629 \| 0,0947 \| 1200 \| 1,8 \| 172,09 \| \|  \|  \| 11,5 \| 76,2 \| 18,5 \| 5,3 \| 46,7818 \| 41,2892 \| 5,8714 \| 0,6629 \| 0,0947 \| 1200 \| 2 \| 163,72 \| \|  \|  \| 11,5 \| 76,2 \| 18,5 \| 5,3 \| 46,7818 \| 41,2892 \| 5,8714 \| 0,6629 \| 0,0947 \| 1200 \| 2,3 \| 154,15 \| \|  \|  \| 11,5 \| 76,2 \| 18,5 \| 5,3 \| 46,7818 \| 41,2892 \| 5,8714 \| 0,6629 \| 0,0947 \| 1500 \| 1,8 \| 207,12 \| \|  \|  \| 11,5 \| 76,2 \| 18,5 \| 5,3 \| 46,7818 \| 41,2892 \| 5,8714 \| 0,6629 \| 0,0947 \| 1500 \| 2 \| 202,73 \| \|  \|  \| 11,5 \| 76,2 \| 18,5 \| 5,3 \| 46,7818 \| 41,2892 \| 5,8714 \| 0,6629 \| 0,0947 \| 1500 \| 2,3 \| 197,01 \| \| 41 \| Corn straw \| 7,4 \| 73,1 \| 19,2 \| 7,7 \| 44,9501 \| 40,7043 \| 5,9072 \| 0,6461 \| 0,0923 \| 600 \| 1,8 \| 39,12 \| \|  \|  \| 7,4 \| 73,1 \| 19,2 \| 7,7 \| 44,9501 \| 40,7043 \| 5,9072 \| 0,6461 \| 0,0923 \| 600 \| 2 \| 31,75 \| \|  \|  \| 7,4 \| 73,1 \| 19,2 \| 7,7 \| 44,9501 \| 40,7043 \| 5,9072 \| 0,6461 \| 0,0923 \| 600 \| 2,3 \| 23,97 \| \|  \|  \| 7,4 \| 73,1 \| 19,2 \| 7,7 \| 44,9501 \| 40,7043 \| 5,9072 \| 0,6461 \| 0,0923 \| 900 \| 1,8 \| 123,05 \| \|  \|  \| 7,4 \| 73,1 \| 19,2 \| 7,7 \| 44,9501 \| 40,7043 \| 5,9072 \| 0,6461 \| 0,0923 \| 900 \| 2 \| 109,58 \| \|  \|  \| 7,4 \| 73,1 \| 19,2 \| 7,7 \| 44,9501 \| 40,7043 \| 5,9072 \| 0,6461 \| 0,0923 \| 900 \| 2,3 \| 95,21 \| \|  \|  \| 7,4 \| 73,1 \| 19,2 \| 7,7 \| 44,9501 \| 40,7043 \| 5,9072 \| 0,6461 \| 0,0923 \| 1200 \| 1,8 \| 161,58 \| \|  \|  \| 7,4 \| 73,1 \| 19,2 \| 7,7 \| 44,9501 \| 40,7043 \| 5,9072 \| 0,6461 \| 0,0923 \| 1200 \| 2 \| 152,04 \| \|  \|  \| 7,4 \| 73,1 \| 19,2 \| 7,7 \| 44,9501 \| 40,7043 \| 5,9072 \| 0,6461 \| 0,0923 \| 1200 \| 2,3 \| 141,43 \| \|  \|  \| 7,4 \| 73,1 \| 19,2 \| 7,7 \| 44,9501 \| 40,7043 \| 5,9072 \| 0,6461 \| 0,0923 \| 1500 \| 1,8 \| 198,97 \| \|  \|  \| 7,4 \| 73,1 \| 19,2 \| 7,7 \| 44,9501 \| 40,7043 \| 5,9072 \| 0,6461 \| 0,0923 \| 1500 \| 2 \| 193,47 \| \|  \|  \| 7,4 \| 73,1 \| 19,2 \| 7,7 \| 44,9501 \| 40,7043 \| 5,9072 \| 0,6461 \| 0,0923 \| 1500 \| 2,3 \| 186,75 \| \| 42 \| Mint straw \| 16,8 \| 69,7 \| 19,5 \| 10,8 \| 45,1352 \| 35,7692 \| 5,5304 \| 2,4976 \| 0,2676 \| 600 \| 1,8 \| 39,42 \| \|  \|  \| 16,8 \| 69,7 \| 19,5 \| 10,8 \| 45,1352 \| 35,7692 \| 5,5304 \| 2,4976 \| 0,2676 \| 600 \| 2 \| 32,86 \| \|  \|  \| 16,8 \| 69,7 \| 19,5 \| 10,8 \| 45,1352 \| 35,7692 \| 5,5304 \| 2,4976 \| 0,2676 \| 600 \| 2,3 \| 25,93 \| \|  \|  \| 16,8 \| 69,7 \| 19,5 \| 10,8 \| 45,1352 \| 35,7692 \| 5,5304 \| 2,4976 \| 0,2676 \| 900 \| 1,8 \| 119,62 \| \|  \|  \| 16,8 \| 69,7 \| 19,5 \| 10,8 \| 45,1352 \| 35,7692 \| 5,5304 \| 2,4976 \| 0,2676 \| 900 \| 2 \| 107,82 \| \|  \|  \| 16,8 \| 69,7 \| 19,5 \| 10,8 \| 45,1352 \| 35,7692 \| 5,5304 \| 2,4976 \| 0,2676 \| 900 \| 2,3 \| 95,13 \| \|  \|  \| 16,8 \| 69,7 \| 19,5 \| 10,8 \| 45,1352 \| 35,7692 \| 5,5304 \| 2,4976 \| 0,2676 \| 1200 \| 1,8 \| 152,96 \| \|  \|  \| 16,8 \| 69,7 \| 19,5 \| 10,8 \| 45,1352 \| 35,7692 \| 5,5304 \| 2,4976 \| 0,2676 \| 1200 \| 2 \| 144,79 \| \|  \|  \| 16,8 \| 69,7 \| 19,5 \| 10,8 \| 45,1352 \| 35,7692 \| 5,5304 \| 2,4976 \| 0,2676 \| 1200 \| 2,3 \| 135,56 \| \|  \|  \| 16,8 \| 69,7 \| 19,5 \| 10,8 \| 45,1352 \| 35,7692 \| 5,5304 \| 2,4976 \| 0,2676 \| 1500 \| 1,8 \| 185,30 \| \|  \|  \| 16,8 \| 69,7 \| 19,5 \| 10,8 \| 45,1352 \| 35,7692 \| 5,5304 \| 2,4976 \| 0,2676 \| 1500 \| 2 \| 180,84 \| \|  \|  \| 16,8 \| 69,7 \| 19,5 \| 10,8 \| 45,1352 \| 35,7692 \| 5,5304 \| 2,4976 \| 0,2676 \| 1500 \| 2,3 \| 175,19 \| \| 43 \| Oat straw \| 8,2 \| 80,5 \| 13,6 \| 5,9 \| 45,9208 \| 41,9686 \| 5,646 \| 0,4705 \| 0,0941 \| 600 \| 1,8 \| 34,55 \| \|  \|  \| 8,2 \| 80,5 \| 13,6 \| 5,9 \| 45,9208 \| 41,9686 \| 5,646 \| 0,4705 \| 0,0941 \| 600 \| 2 \| 27,23 \| \|  \|  \| 8,2 \| 80,5 \| 13,6 \| 5,9 \| 45,9208 \| 41,9686 \| 5,646 \| 0,4705 \| 0,0941 \| 600 \| 2,3 \| 19,50 \| \|  \|  \| 8,2 \| 80,5 \| 13,6 \| 5,9 \| 45,9208 \| 41,9686 \| 5,646 \| 0,4705 \| 0,0941 \| 900 \| 1,8 \| 118,51 \| \|  \|  \| 8,2 \| 80,5 \| 13,6 \| 5,9 \| 45,9208 \| 41,9686 \| 5,646 \| 0,4705 \| 0,0941 \| 900 \| 2 \| 105,08 \| \|  \|  \| 8,2 \| 80,5 \| 13,6 \| 5,9 \| 45,9208 \| 41,9686 \| 5,646 \| 0,4705 \| 0,0941 \| 900 \| 2,3 \| 90,78 \| \|  \|  \| 8,2 \| 80,5 \| 13,6 \| 5,9 \| 45,9208 \| 41,9686 \| 5,646 \| 0,4705 \| 0,0941 \| 1200 \| 1,8 \| 156,66 \| \|  \|  \| 8,2 \| 80,5 \| 13,6 \| 5,9 \| 45,9208 \| 41,9686 \| 5,646 \| 0,4705 \| 0,0941 \| 1200 \| 2 \| 147,11 \| \|  \|  \| 8,2 \| 80,5 \| 13,6 \| 5,9 \| 45,9208 \| 41,9686 \| 5,646 \| 0,4705 \| 0,0941 \| 1200 \| 2,3 \| 136,51 \| \|  \|  \| 8,2 \| 80,5 \| 13,6 \| 5,9 \| 45,9208 \| 41,9686 \| 5,646 \| 0,4705 \| 0,0941 \| 1500 \| 1,8 \| 193,69 \| \|  \|  \| 8,2 \| 80,5 \| 13,6 \| 5,9 \| 45,9208 \| 41,9686 \| 5,646 \| 0,4705 \| 0,0941 \| 1500 \| 2 \| 188,14 \| \|  \|  \| 8,2 \| 80,5 \| 13,6 \| 5,9 \| 45,9208 \| 41,9686 \| 5,646 \| 0,4705 \| 0,0941 \| 1500 \| 2,3 \| 181,38 \| \| 44 \| Rape straw \| 8,7 \| 77,4 \| 17,9 \| 4,7 \| 46,2205 \| 42,4085 \| 6,0992 \| 0,4765 \| 0,0953 \| 600 \| 1,8 \| 52,02 \| \|  \|  \| 8,7 \| 77,4 \| 17,9 \| 4,7 \| 46,2205 \| 42,4085 \| 6,0992 \| 0,4765 \| 0,0953 \| 600 \| 2 \| 44,99 \| \|  \|  \| 8,7 \| 77,4 \| 17,9 \| 4,7 \| 46,2205 \| 42,4085 \| 6,0992 \| 0,4765 \| 0,0953 \| 600 \| 2,3 \| 37,53 \| \|  \|  \| 8,7 \| 77,4 \| 17,9 \| 4,7 \| 46,2205 \| 42,4085 \| 6,0992 \| 0,4765 \| 0,0953 \| 900 \| 1,8 \| 137,23 \| \|  \|  \| 8,7 \| 77,4 \| 17,9 \| 4,7 \| 46,2205 \| 42,4085 \| 6,0992 \| 0,4765 \| 0,0953 \| 900 \| 2 \| 124,46 \| \|  \|  \| 8,7 \| 77,4 \| 17,9 \| 4,7 \| 46,2205 \| 42,4085 \| 6,0992 \| 0,4765 \| 0,0953 \| 900 \| 2,3 \| 110,70 \| \|  \|  \| 8,7 \| 77,4 \| 17,9 \| 4,7 \| 46,2205 \| 42,4085 \| 6,0992 \| 0,4765 \| 0,0953 \| 1200 \| 1,8 \| 175,21 \| \|  \|  \| 8,7 \| 77,4 \| 17,9 \| 4,7 \| 46,2205 \| 42,4085 \| 6,0992 \| 0,4765 \| 0,0953 \| 1200 \| 2 \| 166,41 \| \|  \|  \| 8,7 \| 77,4 \| 17,9 \| 4,7 \| 46,2205 \| 42,4085 \| 6,0992 \| 0,4765 \| 0,0953 \| 1200 \| 2,3 \| 156,43 \| \|  \|  \| 8,7 \| 77,4 \| 17,9 \| 4,7 \| 46,2205 \| 42,4085 \| 6,0992 \| 0,4765 \| 0,0953 \| 1500 \| 1,8 \| 211,91 \| \|  \|  \| 8,7 \| 77,4 \| 17,9 \| 4,7 \| 46,2205 \| 42,4085 \| 6,0992 \| 0,4765 \| 0,0953 \| 1500 \| 2 \| 207,19 \| \|  \|  \| 8,7 \| 77,4 \| 17,9 \| 4,7 \| 46,2205 \| 42,4085 \| 6,0992 \| 0,4765 \| 0,0953 \| 1500 \| 2,3 \| 201,14 \| \| 45 \| Rice straw \| 7,6 \| 64,3 \| 15,6 \| 20,1 \| 40,0299 \| 34,357 \| 4,5543 \| 0,799 \| 0,1598 \| 600 \| 1,8 \| 0,00 \| \|  \|  \| 7,6 \| 64,3 \| 15,6 \| 20,1 \| 40,0299 \| 34,357 \| 4,5543 \| 0,799 \| 0,1598 \| 600 \| 2 \| 0,00 \| \|  \|  \| 7,6 \| 64,3 \| 15,6 \| 20,1 \| 40,0299 \| 34,357 \| 4,5543 \| 0,799 \| 0,1598 \| 600 \| 2,3 \| 0,00 \| \|  \|  \| 7,6 \| 64,3 \| 15,6 \| 20,1 \| 40,0299 \| 34,357 \| 4,5543 \| 0,799 \| 0,1598 \| 900 \| 1,8 \| 32,05 \| \|  \|  \| 7,6 \| 64,3 \| 15,6 \| 20,1 \| 40,0299 \| 34,357 \| 4,5543 \| 0,799 \| 0,1598 \| 900 \| 2 \| 16,63 \| \|  \|  \| 7,6 \| 64,3 \| 15,6 \| 20,1 \| 40,0299 \| 34,357 \| 4,5543 \| 0,799 \| 0,1598 \| 900 \| 2,3 \| 0,59 \| \|  \|  \| 7,6 \| 64,3 \| 15,6 \| 20,1 \| 40,0299 \| 34,357 \| 4,5543 \| 0,799 \| 0,1598 \| 1200 \| 1,8 \| 69,62 \| \|  \|  \| 7,6 \| 64,3 \| 15,6 \| 20,1 \| 40,0299 \| 34,357 \| 4,5543 \| 0,799 \| 0,1598 \| 1200 \| 2 \| 57,74 \| \|  \|  \| 7,6 \| 64,3 \| 15,6 \| 20,1 \| 40,0299 \| 34,357 \| 4,5543 \| 0,799 \| 0,1598 \| 1200 \| 2,3 \| 45,19 \| \|  \|  \| 7,6 \| 64,3 \| 15,6 \| 20,1 \| 40,0299 \| 34,357 \| 4,5543 \| 0,799 \| 0,1598 \| 1500 \| 1,8 \| 106,69 \| \|  \|  \| 7,6 \| 64,3 \| 15,6 \| 20,1 \| 40,0299 \| 34,357 \| 4,5543 \| 0,799 \| 0,1598 \| 1500 \| 2 \| 98,41 \| \|  \|  \| 7,6 \| 64,3 \| 15,6 \| 20,1 \| 40,0299 \| 34,357 \| 4,5543 \| 0,799 \| 0,1598 \| 1500 \| 2,3 \| 89,41 \| \| 46 \| Straw \| 12,4 \| 73,4 \| 15,8 \| 10,8 \| 43,1728 \| 39,694 \| 4,9952 \| 0,892 \| 0,446 \| 600 \| 1,8 \| 0,00 \| \|  \|  \| 12,4 \| 73,4 \| 15,8 \| 10,8 \| 43,1728 \| 39,694 \| 4,9952 \| 0,892 \| 0,446 \| 600 \| 2 \| 0,00 \| \|  \|  \| 12,4 \| 73,4 \| 15,8 \| 10,8 \| 43,1728 \| 39,694 \| 4,9952 \| 0,892 \| 0,446 \| 600 \| 2,3 \| 0,00 \| \|  \|  \| 12,4 \| 73,4 \| 15,8 \| 10,8 \| 43,1728 \| 39,694 \| 4,9952 \| 0,892 \| 0,446 \| 900 \| 1,8 \| 65,17 \| \|  \|  \| 12,4 \| 73,4 \| 15,8 \| 10,8 \| 43,1728 \| 39,694 \| 4,9952 \| 0,892 \| 0,446 \| 900 \| 2 \| 51,21 \| \|  \|  \| 12,4 \| 73,4 \| 15,8 \| 10,8 \| 43,1728 \| 39,694 \| 4,9952 \| 0,892 \| 0,446 \| 900 \| 2,3 \| 36,60 \| \|  \|  \| 12,4 \| 73,4 \| 15,8 \| 10,8 \| 43,1728 \| 39,694 \| 4,9952 \| 0,892 \| 0,446 \| 1200 \| 1,8 \| 101,62 \| \|  \|  \| 12,4 \| 73,4 \| 15,8 \| 10,8 \| 43,1728 \| 39,694 \| 4,9952 \| 0,892 \| 0,446 \| 1200 \| 2 \| 91,11 \| \|  \|  \| 12,4 \| 73,4 \| 15,8 \| 10,8 \| 43,1728 \| 39,694 \| 4,9952 \| 0,892 \| 0,446 \| 1200 \| 2,3 \| 79,86 \| \|  \|  \| 12,4 \| 73,4 \| 15,8 \| 10,8 \| 43,1728 \| 39,694 \| 4,9952 \| 0,892 \| 0,446 \| 1500 \| 1,8 \| 137,40 \| \|  \|  \| 12,4 \| 73,4 \| 15,8 \| 10,8 \| 43,1728 \| 39,694 \| 4,9952 \| 0,892 \| 0,446 \| 1500 \| 2 \| 130,46 \| \|  \|  \| 12,4 \| 73,4 \| 15,8 \| 10,8 \| 43,1728 \| 39,694 \| 4,9952 \| 0,892 \| 0,446 \| 1500 \| 2,3 \| 122,71 \| \| 47 \| Wheat straw \| 10,1 \| 74,8 \| 18,1 \| 7,1 \| 45,8926 \| 40,5044 \| 5,6669 \| 0,6503 \| 0,1858 \| 600 \| 1,8 \| 39,01 \| \|  \|  \| 10,1 \| 74,8 \| 18,1 \| 7,1 \| 45,8926 \| 40,5044 \| 5,6669 \| 0,6503 \| 0,1858 \| 600 \| 2 \| 31,92 \| \|  \|  \| 10,1 \| 74,8 \| 18,1 \| 7,1 \| 45,8926 \| 40,5044 \| 5,6669 \| 0,6503 \| 0,1858 \| 600 \| 2,3 \| 24,43 \| \|  \|  \| 10,1 \| 74,8 \| 18,1 \| 7,1 \| 45,8926 \| 40,5044 \| 5,6669 \| 0,6503 \| 0,1858 \| 900 \| 1,8 \| 122,87 \| \|  \|  \| 10,1 \| 74,8 \| 18,1 \| 7,1 \| 45,8926 \| 40,5044 \| 5,6669 \| 0,6503 \| 0,1858 \| 900 \| 2 \| 109,94 \| \|  \|  \| 10,1 \| 74,8 \| 18,1 \| 7,1 \| 45,8926 \| 40,5044 \| 5,6669 \| 0,6503 \| 0,1858 \| 900 \| 2,3 \| 96,11 \| \|  \|  \| 10,1 \| 74,8 \| 18,1 \| 7,1 \| 45,8926 \| 40,5044 \| 5,6669 \| 0,6503 \| 0,1858 \| 1200 \| 1,8 \| 159,83 \| \|  \|  \| 10,1 \| 74,8 \| 18,1 \| 7,1 \| 45,8926 \| 40,5044 \| 5,6669 \| 0,6503 \| 0,1858 \| 1200 \| 2 \| 150,75 \| \|  \|  \| 10,1 \| 74,8 \| 18,1 \| 7,1 \| 45,8926 \| 40,5044 \| 5,6669 \| 0,6503 \| 0,1858 \| 1200 \| 2,3 \| 140,59 \| \|  \|  \| 10,1 \| 74,8 \| 18,1 \| 7,1 \| 45,8926 \| 40,5044 \| 5,6669 \| 0,6503 \| 0,1858 \| 1500 \| 1,8 \| 195,68 \| \|  \|  \| 10,1 \| 74,8 \| 18,1 \| 7,1 \| 45,8926 \| 40,5044 \| 5,6669 \| 0,6503 \| 0,1858 \| 1500 \| 2 \| 190,55 \| \|  \|  \| 10,1 \| 74,8 \| 18,1 \| 7,1 \| 45,8926 \| 40,5044 \| 5,6669 \| 0,6503 \| 0,1858 \| 1500 \| 2,3 \| 184,20 \| \| 48 \| Almond hulls \| 6,5 \| 73,8 \| 20,1 \| 6,1 \| 47,5134 \| 39,1563 \| 6,0096 \| 1,1268 \| 0,0939 \| 600 \| 1,8 \| 72,56 \| \|  \|  \| 6,5 \| 73,8 \| 20,1 \| 6,1 \| 47,5134 \| 39,1563 \| 6,0096 \| 1,1268 \| 0,0939 \| 600 \| 2 \| 65,68 \| \|  \|  \| 6,5 \| 73,8 \| 20,1 \| 6,1 \| 47,5134 \| 39,1563 \| 6,0096 \| 1,1268 \| 0,0939 \| 600 \| 2,3 \| 58,33 \| \|  \|  \| 6,5 \| 73,8 \| 20,1 \| 6,1 \| 47,5134 \| 39,1563 \| 6,0096 \| 1,1268 \| 0,0939 \| 900 \| 1,8 \| 167,01 \| \|  \|  \| 6,5 \| 73,8 \| 20,1 \| 6,1 \| 47,5134 \| 39,1563 \| 6,0096 \| 1,1268 \| 0,0939 \| 900 \| 2 \| 154,96 \| \|  \|  \| 6,5 \| 73,8 \| 20,1 \| 6,1 \| 47,5134 \| 39,1563 \| 6,0096 \| 1,1268 \| 0,0939 \| 900 \| 2,3 \| 141,62 \| \|  \|  \| 6,5 \| 73,8 \| 20,1 \| 6,1 \| 47,5134 \| 39,1563 \| 6,0096 \| 1,1268 \| 0,0939 \| 1200 \| 1,8 \| 204,21 \| \|  \|  \| 6,5 \| 73,8 \| 20,1 \| 6,1 \| 47,5134 \| 39,1563 \| 6,0096 \| 1,1268 \| 0,0939 \| 1200 \| 2 \| 196,48 \| \|  \|  \| 6,5 \| 73,8 \| 20,1 \| 6,1 \| 47,5134 \| 39,1563 \| 6,0096 \| 1,1268 \| 0,0939 \| 1200 \| 2,3 \| 187,24 \| \|  \|  \| 6,5 \| 73,8 \| 20,1 \| 6,1 \| 47,5134 \| 39,1563 \| 6,0096 \| 1,1268 \| 0,0939 \| 1500 \| 1,8 \| 240,01 \| \|  \|  \| 6,5 \| 73,8 \| 20,1 \| 6,1 \| 47,5134 \| 39,1563 \| 6,0096 \| 1,1268 \| 0,0939 \| 1500 \| 2 \| 236,61 \| \|  \|  \| 6,5 \| 73,8 \| 20,1 \| 6,1 \| 47,5134 \| 39,1563 \| 6,0096 \| 1,1268 \| 0,0939 \| 1500 \| 2,3 \| 231,61 \| \| 49 \| Almond shells \| 7,2 \| 74,9 \| 21,8 \| 3,3 \| 48,6401 \| 41,0975 \| 5,9954 \| 0,967 \| 0 \| 600 \| 1,8 \| 75,80 \| \|  \|  \| 7,2 \| 74,9 \| 21,8 \| 3,3 \| 48,6401 \| 41,0975 \| 5,9954 \| 0,967 \| 0 \| 600 \| 2 \| 69,06 \| \|  \|  \| 7,2 \| 74,9 \| 21,8 \| 3,3 \| 48,6401 \| 41,0975 \| 5,9954 \| 0,967 \| 0 \| 600 \| 2,3 \| 61,83 \| \|  \|  \| 7,2 \| 74,9 \| 21,8 \| 3,3 \| 48,6401 \| 41,0975 \| 5,9954 \| 0,967 \| 0 \| 900 \| 1,8 \| 170,65 \| \|  \|  \| 7,2 \| 74,9 \| 21,8 \| 3,3 \| 48,6401 \| 41,0975 \| 5,9954 \| 0,967 \| 0 \| 900 \| 2 \| 158,87 \| \|  \|  \| 7,2 \| 74,9 \| 21,8 \| 3,3 \| 48,6401 \| 41,0975 \| 5,9954 \| 0,967 \| 0 \| 900 \| 2,3 \| 145,76 \| \|  \|  \| 7,2 \| 74,9 \| 21,8 \| 3,3 \| 48,6401 \| 41,0975 \| 5,9954 \| 0,967 \| 0 \| 1200 \| 1,8 \| 207,61 \| \|  \|  \| 7,2 \| 74,9 \| 21,8 \| 3,3 \| 48,6401 \| 41,0975 \| 5,9954 \| 0,967 \| 0 \| 1200 \| 2 \| 200,17 \| \|  \|  \| 7,2 \| 74,9 \| 21,8 \| 3,3 \| 48,6401 \| 41,0975 \| 5,9954 \| 0,967 \| 0 \| 1200 \| 2,3 \| 191,18 \| \|  \|  \| 7,2 \| 74,9 \| 21,8 \| 3,3 \| 48,6401 \| 41,0975 \| 5,9954 \| 0,967 \| 0 \| 1500 \| 1,8 \| 243,13 \| \|  \|  \| 7,2 \| 74,9 \| 21,8 \| 3,3 \| 48,6401 \| 41,0975 \| 5,9954 \| 0,967 \| 0 \| 1500 \| 2 \| 240,02 \| \|  \|  \| 7,2 \| 74,9 \| 21,8 \| 3,3 \| 48,6401 \| 41,0975 \| 5,9954 \| 0,967 \| 0 \| 1500 \| 2,3 \| 235,26 \| \| 50 \| Coconut shells \| 4,4 \| 73,8 \| 23 \| 3,2 \| 49,4648 \| 41,7208 \| 5,4208 \| 0,0968 \| 0,0968 \| 600 \| 1,8 \| 53,08 \| \|  \|  \| 4,4 \| 73,8 \| 23 \| 3,2 \| 49,4648 \| 41,7208 \| 5,4208 \| 0,0968 \| 0,0968 \| 600 \| 2 \| 45,76 \| \|  \|  \| 4,4 \| 73,8 \| 23 \| 3,2 \| 49,4648 \| 41,7208 \| 5,4208 \| 0,0968 \| 0,0968 \| 600 \| 2,3 \| 37,99 \| \|  \|  \| 4,4 \| 73,8 \| 23 \| 3,2 \| 49,4648 \| 41,7208 \| 5,4208 \| 0,0968 \| 0,0968 \| 900 \| 1,8 \| 149,46 \| \|  \|  \| 4,4 \| 73,8 \| 23 \| 3,2 \| 49,4648 \| 41,7208 \| 5,4208 \| 0,0968 \| 0,0968 \| 900 \| 2 \| 136,34 \| \|  \|  \| 4,4 \| 73,8 \| 23 \| 3,2 \| 49,4648 \| 41,7208 \| 5,4208 \| 0,0968 \| 0,0968 \| 900 \| 2,3 \| 122,06 \| \|  \|  \| 4,4 \| 73,8 \| 23 \| 3,2 \| 49,4648 \| 41,7208 \| 5,4208 \| 0,0968 \| 0,0968 \| 1200 \| 1,8 \| 187,54 \| \|  \|  \| 4,4 \| 73,8 \| 23 \| 3,2 \| 49,4648 \| 41,7208 \| 5,4208 \| 0,0968 \| 0,0968 \| 1200 \| 2 \| 178,72 \| \|  \|  \| 4,4 \| 73,8 \| 23 \| 3,2 \| 49,4648 \| 41,7208 \| 5,4208 \| 0,0968 \| 0,0968 \| 1200 \| 2,3 \| 168,51 \| \|  \|  \| 4,4 \| 73,8 \| 23 \| 3,2 \| 49,4648 \| 41,7208 \| 5,4208 \| 0,0968 \| 0,0968 \| 1500 \| 1,8 \| 224,33 \| \|  \|  \| 4,4 \| 73,8 \| 23 \| 3,2 \| 49,4648 \| 41,7208 \| 5,4208 \| 0,0968 \| 0,0968 \| 1500 \| 2 \| 219,86 \| \|  \|  \| 4,4 \| 73,8 \| 23 \| 3,2 \| 49,4648 \| 41,7208 \| 5,4208 \| 0,0968 \| 0,0968 \| 1500 \| 2,3 \| 213,86 \| \| 51 \| Coffee husks \| 10,8 \| 76,5 \| 20,7 \| 2,8 \| 44,1288 \| 46,9476 \| 4,7628 \| 1,0692 \| 0,2916 \| 600 \| 1,8 \| 0,00 \| \|  \|  \| 10,8 \| 76,5 \| 20,7 \| 2,8 \| 44,1288 \| 46,9476 \| 4,7628 \| 1,0692 \| 0,2916 \| 600 \| 2 \| 0,00 \| \|  \|  \| 10,8 \| 76,5 \| 20,7 \| 2,8 \| 44,1288 \| 46,9476 \| 4,7628 \| 1,0692 \| 0,2916 \| 600 \| 2,3 \| 0,00 \| \|  \|  \| 10,8 \| 76,5 \| 20,7 \| 2,8 \| 44,1288 \| 46,9476 \| 4,7628 \| 1,0692 \| 0,2916 \| 900 \| 1,8 \| 37,65 \| \|  \|  \| 10,8 \| 76,5 \| 20,7 \| 2,8 \| 44,1288 \| 46,9476 \| 4,7628 \| 1,0692 \| 0,2916 \| 900 \| 2 \| 22,84 \| \|  \|  \| 10,8 \| 76,5 \| 20,7 \| 2,8 \| 44,1288 \| 46,9476 \| 4,7628 \| 1,0692 \| 0,2916 \| 900 \| 2,3 \| 7,44 \| \|  \|  \| 10,8 \| 76,5 \| 20,7 \| 2,8 \| 44,1288 \| 46,9476 \| 4,7628 \| 1,0692 \| 0,2916 \| 1200 \| 1,8 \| 76,09 \| \|  \|  \| 10,8 \| 76,5 \| 20,7 \| 2,8 \| 44,1288 \| 46,9476 \| 4,7628 \| 1,0692 \| 0,2916 \| 1200 \| 2 \| 64,67 \| \|  \|  \| 10,8 \| 76,5 \| 20,7 \| 2,8 \| 44,1288 \| 46,9476 \| 4,7628 \| 1,0692 \| 0,2916 \| 1200 \| 2,3 \| 52,60 \| \|  \|  \| 10,8 \| 76,5 \| 20,7 \| 2,8 \| 44,1288 \| 46,9476 \| 4,7628 \| 1,0692 \| 0,2916 \| 1500 \| 1,8 \| 113,96 \| \|  \|  \| 10,8 \| 76,5 \| 20,7 \| 2,8 \| 44,1288 \| 46,9476 \| 4,7628 \| 1,0692 \| 0,2916 \| 1500 \| 2 \| 106,06 \| \|  \|  \| 10,8 \| 76,5 \| 20,7 \| 2,8 \| 44,1288 \| 46,9476 \| 4,7628 \| 1,0692 \| 0,2916 \| 1500 \| 2,3 \| 97,47 \| \| 52 \| Cotton husks \| 6,9 \| 78,4 \| 18,2 \| 3,4 \| 48,6864 \| 38,4468 \| 8,1144 \| 1,3524 \| 0 \| 600 \| 1,8 \| 158,13 \| \|  \|  \| 6,9 \| 78,4 \| 18,2 \| 3,4 \| 48,6864 \| 38,4468 \| 8,1144 \| 1,3524 \| 0 \| 600 \| 2 \| 153,29 \| \|  \|  \| 6,9 \| 78,4 \| 18,2 \| 3,4 \| 48,6864 \| 38,4468 \| 8,1144 \| 1,3524 \| 0 \| 600 \| 2,3 \| 147,49 \| \|  \|  \| 6,9 \| 78,4 \| 18,2 \| 3,4 \| 48,6864 \| 38,4468 \| 8,1144 \| 1,3524 \| 0 \| 900 \| 1,8 \| 256,51 \| \|  \|  \| 6,9 \| 78,4 \| 18,2 \| 3,4 \| 48,6864 \| 38,4468 \| 8,1144 \| 1,3524 \| 0 \| 900 \| 2 \| 249,65 \| \|  \|  \| 6,9 \| 78,4 \| 18,2 \| 3,4 \| 48,6864 \| 38,4468 \| 8,1144 \| 1,3524 \| 0 \| 900 \| 2,3 \| 240,42 \| \|  \|  \| 6,9 \| 78,4 \| 18,2 \| 3,4 \| 48,6864 \| 38,4468 \| 8,1144 \| 1,3524 \| 0 \| 1200 \| 1,8 \| 291,17 \| \|  \|  \| 6,9 \| 78,4 \| 18,2 \| 3,4 \| 48,6864 \| 38,4468 \| 8,1144 \| 1,3524 \| 0 \| 1200 \| 2 \| 288,93 \| \|  \|  \| 6,9 \| 78,4 \| 18,2 \| 3,4 \| 48,6864 \| 38,4468 \| 8,1144 \| 1,3524 \| 0 \| 1200 \| 2,3 \| 284,44 \| \|  \|  \| 6,9 \| 78,4 \| 18,2 \| 3,4 \| 48,6864 \| 38,4468 \| 8,1144 \| 1,3524 \| 0 \| 1500 \| 1,8 \| 324,46 \| \|  \|  \| 6,9 \| 78,4 \| 18,2 \| 3,4 \| 48,6864 \| 38,4468 \| 8,1144 \| 1,3524 \| 0 \| 1500 \| 2 \| 326,38 \| \|  \|  \| 6,9 \| 78,4 \| 18,2 \| 3,4 \| 48,6864 \| 38,4468 \| 8,1144 \| 1,3524 \| 0 \| 1500 \| 2,3 \| 326,36 \| \| 53 \| Grape marc \| 10 \| 65,8 \| 26,4 \| 7,8 \| 49,788 \| 34,4828 \| 5,6242 \| 2,2128 \| 0,0922 \| 600 \| 1,8 \| 91,01 \| \|  \|  \| 10 \| 65,8 \| 26,4 \| 7,8 \| 49,788 \| 34,4828 \| 5,6242 \| 2,2128 \| 0,0922 \| 600 \| 2 \| 84,88 \| \|  \|  \| 10 \| 65,8 \| 26,4 \| 7,8 \| 49,788 \| 34,4828 \| 5,6242 \| 2,2128 \| 0,0922 \| 600 \| 2,3 \| 78,17 \| \|  \|  \| 10 \| 65,8 \| 26,4 \| 7,8 \| 49,788 \| 34,4828 \| 5,6242 \| 2,2128 \| 0,0922 \| 900 \| 1,8 \| 191,28 \| \|  \|  \| 10 \| 65,8 \| 26,4 \| 7,8 \| 49,788 \| 34,4828 \| 5,6242 \| 2,2128 \| 0,0922 \| 900 \| 2 \| 181,46 \| \|  \|  \| 10 \| 65,8 \| 26,4 \| 7,8 \| 49,788 \| 34,4828 \| 5,6242 \| 2,2128 \| 0,0922 \| 900 \| 2,3 \| 169,88 \| \|  \|  \| 10 \| 65,8 \| 26,4 \| 7,8 \| 49,788 \| 34,4828 \| 5,6242 \| 2,2128 \| 0,0922 \| 1200 \| 1,8 \| 224,25 \| \|  \|  \| 10 \| 65,8 \| 26,4 \| 7,8 \| 49,788 \| 34,4828 \| 5,6242 \| 2,2128 \| 0,0922 \| 1200 \| 2 \| 218,84 \| \|  \|  \| 10 \| 65,8 \| 26,4 \| 7,8 \| 49,788 \| 34,4828 \| 5,6242 \| 2,2128 \| 0,0922 \| 1200 \| 2,3 \| 211,55 \| \|  \|  \| 10 \| 65,8 \| 26,4 \| 7,8 \| 49,788 \| 34,4828 \| 5,6242 \| 2,2128 \| 0,0922 \| 1500 \| 1,8 \| 255,98 \| \|  \|  \| 10 \| 65,8 \| 26,4 \| 7,8 \| 49,788 \| 34,4828 \| 5,6242 \| 2,2128 \| 0,0922 \| 1500 \| 2 \| 254,79 \| \|  \|  \| 10 \| 65,8 \| 26,4 \| 7,8 \| 49,788 \| 34,4828 \| 5,6242 \| 2,2128 \| 0,0922 \| 1500 \| 2,3 \| 251,79 \| \| 54 \| Groundnut shells \| 7,9 \| 73,9 \| 22,7 \| 3,4 \| 49,1694 \| 39,0264 \| 7,245 \| 1,1592 \| 0 \| 600 \| 1,8 \| 129,63 \| \|  \|  \| 7,9 \| 73,9 \| 22,7 \| 3,4 \| 49,1694 \| 39,0264 \| 7,245 \| 1,1592 \| 0 \| 600 \| 2 \| 124,07 \| \|  \|  \| 7,9 \| 73,9 \| 22,7 \| 3,4 \| 49,1694 \| 39,0264 \| 7,245 \| 1,1592 \| 0 \| 600 \| 2,3 \| 117,77 \| \|  \|  \| 7,9 \| 73,9 \| 22,7 \| 3,4 \| 49,1694 \| 39,0264 \| 7,245 \| 1,1592 \| 0 \| 900 \| 1,8 \| 227,81 \| \|  \|  \| 7,9 \| 73,9 \| 22,7 \| 3,4 \| 49,1694 \| 39,0264 \| 7,245 \| 1,1592 \| 0 \| 900 \| 2 \| 219,29 \| \|  \|  \| 7,9 \| 73,9 \| 22,7 \| 3,4 \| 49,1694 \| 39,0264 \| 7,245 \| 1,1592 \| 0 \| 900 \| 2,3 \| 208,73 \| \|  \|  \| 7,9 \| 73,9 \| 22,7 \| 3,4 \| 49,1694 \| 39,0264 \| 7,245 \| 1,1592 \| 0 \| 1200 \| 1,8 \| 262,62 \| \|  \|  \| 7,9 \| 73,9 \| 22,7 \| 3,4 \| 49,1694 \| 39,0264 \| 7,245 \| 1,1592 \| 0 \| 1200 \| 2 \| 258,70 \| \|  \|  \| 7,9 \| 73,9 \| 22,7 \| 3,4 \| 49,1694 \| 39,0264 \| 7,245 \| 1,1592 \| 0 \| 1200 \| 2,3 \| 252,70 \| \|  \|  \| 7,9 \| 73,9 \| 22,7 \| 3,4 \| 49,1694 \| 39,0264 \| 7,245 \| 1,1592 \| 0 \| 1500 \| 1,8 \| 295,95 \| \|  \|  \| 7,9 \| 73,9 \| 22,7 \| 3,4 \| 49,1694 \| 39,0264 \| 7,245 \| 1,1592 \| 0 \| 1500 \| 2 \| 296,32 \| \|  \|  \| 7,9 \| 73,9 \| 22,7 \| 3,4 \| 49,1694 \| 39,0264 \| 7,245 \| 1,1592 \| 0 \| 1500 \| 2,3 \| 294,77 \| \| 55 \| Hazelnut shells \| 7,2 \| 77,1 \| 21,4 \| 1,5 \| 50,7275 \| 40,976 \| 5,4175 \| 1,379 \| 0 \| 600 \| 1,8 \| 75,14 \| \|  \|  \| 7,2 \| 77,1 \| 21,4 \| 1,5 \| 50,7275 \| 40,976 \| 5,4175 \| 1,379 \| 0 \| 600 \| 2 \| 68,46 \| \|  \|  \| 7,2 \| 77,1 \| 21,4 \| 1,5 \| 50,7275 \| 40,976 \| 5,4175 \| 1,379 \| 0 \| 600 \| 2,3 \| 61,28 \| \|  \|  \| 7,2 \| 77,1 \| 21,4 \| 1,5 \| 50,7275 \| 40,976 \| 5,4175 \| 1,379 \| 0 \| 900 \| 1,8 \| 174,74 \| \|  \|  \| 7,2 \| 77,1 \| 21,4 \| 1,5 \| 50,7275 \| 40,976 \| 5,4175 \| 1,379 \| 0 \| 900 \| 2 \| 163,26 \| \|  \|  \| 7,2 \| 77,1 \| 21,4 \| 1,5 \| 50,7275 \| 40,976 \| 5,4175 \| 1,379 \| 0 \| 900 \| 2,3 \| 150,32 \| \|  \|  \| 7,2 \| 77,1 \| 21,4 \| 1,5 \| 50,7275 \| 40,976 \| 5,4175 \| 1,379 \| 0 \| 1200 \| 1,8 \| 210,67 \| \|  \|  \| 7,2 \| 77,1 \| 21,4 \| 1,5 \| 50,7275 \| 40,976 \| 5,4175 \| 1,379 \| 0 \| 1200 \| 2 \| 203,59 \| \|  \|  \| 7,2 \| 77,1 \| 21,4 \| 1,5 \| 50,7275 \| 40,976 \| 5,4175 \| 1,379 \| 0 \| 1200 \| 2,3 \| 194,85 \| \|  \|  \| 7,2 \| 77,1 \| 21,4 \| 1,5 \| 50,7275 \| 40,976 \| 5,4175 \| 1,379 \| 0 \| 1500 \| 1,8 \| 245,20 \| \|  \|  \| 7,2 \| 77,1 \| 21,4 \| 1,5 \| 50,7275 \| 40,976 \| 5,4175 \| 1,379 \| 0 \| 1500 \| 2 \| 242,49 \| \|  \|  \| 7,2 \| 77,1 \| 21,4 \| 1,5 \| 50,7275 \| 40,976 \| 5,4175 \| 1,379 \| 0 \| 1500 \| 2,3 \| 238,04 \| \| 56 \| Mustard husks \| 5,6 \| 72,6 \| 23,3 \| 4,1 \| 43,9222 \| 42,5796 \| 8,8228 \| 0,3836 \| 0,1918 \| 600 \| 1,8 \| 130,41 \| \|  \|  \| 5,6 \| 72,6 \| 23,3 \| 4,1 \| 43,9222 \| 42,5796 \| 8,8228 \| 0,3836 \| 0,1918 \| 600 \| 2 \| 124,29 \| \|  \|  \| 5,6 \| 72,6 \| 23,3 \| 4,1 \| 43,9222 \| 42,5796 \| 8,8228 \| 0,3836 \| 0,1918 \| 600 \| 2,3 \| 117,54 \| \|  \|  \| 5,6 \| 72,6 \| 23,3 \| 4,1 \| 43,9222 \| 42,5796 \| 8,8228 \| 0,3836 \| 0,1918 \| 900 \| 1,8 \| 217,68 \| \|  \|  \| 5,6 \| 72,6 \| 23,3 \| 4,1 \| 43,9222 \| 42,5796 \| 8,8228 \| 0,3836 \| 0,1918 \| 900 \| 2 \| 207,22 \| \|  \|  \| 5,6 \| 72,6 \| 23,3 \| 4,1 \| 43,9222 \| 42,5796 \| 8,8228 \| 0,3836 \| 0,1918 \| 900 \| 2,3 \| 195,17 \| \|  \|  \| 5,6 \| 72,6 \| 23,3 \| 4,1 \| 43,9222 \| 42,5796 \| 8,8228 \| 0,3836 \| 0,1918 \| 1200 \| 1,8 \| 257,28 \| \|  \|  \| 5,6 \| 72,6 \| 23,3 \| 4,1 \| 43,9222 \| 42,5796 \| 8,8228 \| 0,3836 \| 0,1918 \| 1200 \| 2 \| 251,39 \| \|  \|  \| 5,6 \| 72,6 \| 23,3 \| 4,1 \| 43,9222 \| 42,5796 \| 8,8228 \| 0,3836 \| 0,1918 \| 1200 \| 2,3 \| 243,73 \| \|  \|  \| 5,6 \| 72,6 \| 23,3 \| 4,1 \| 43,9222 \| 42,5796 \| 8,8228 \| 0,3836 \| 0,1918 \| 1500 \| 1,8 \| 295,08 \| \|  \|  \| 5,6 \| 72,6 \| 23,3 \| 4,1 \| 43,9222 \| 42,5796 \| 8,8228 \| 0,3836 \| 0,1918 \| 1500 \| 2 \| 293,67 \| \|  \|  \| 5,6 \| 72,6 \| 23,3 \| 4,1 \| 43,9222 \| 42,5796 \| 8,8228 \| 0,3836 \| 0,1918 \| 1500 \| 2,3 \| 290,48 \| \| 57 \| Olive husks \| 6,8 \| 79 \| 18,7 \| 2,3 \| 48,85 \| 41,1317 \| 6,0574 \| 1,5632 \| 0,0977 \| 600 \| 1,8 \| 80,78 \| \|  \|  \| 6,8 \| 79 \| 18,7 \| 2,3 \| 48,85 \| 41,1317 \| 6,0574 \| 1,5632 \| 0,0977 \| 600 \| 2 \| 74,09 \| \|  \|  \| 6,8 \| 79 \| 18,7 \| 2,3 \| 48,85 \| 41,1317 \| 6,0574 \| 1,5632 \| 0,0977 \| 600 \| 2,3 \| 66,90 \| \|  \|  \| 6,8 \| 79 \| 18,7 \| 2,3 \| 48,85 \| 41,1317 \| 6,0574 \| 1,5632 \| 0,0977 \| 900 \| 1,8 \| 176,67 \| \|  \|  \| 6,8 \| 79 \| 18,7 \| 2,3 \| 48,85 \| 41,1317 \| 6,0574 \| 1,5632 \| 0,0977 \| 900 \| 2 \| 165,06 \| \|  \|  \| 6,8 \| 79 \| 18,7 \| 2,3 \| 48,85 \| 41,1317 \| 6,0574 \| 1,5632 \| 0,0977 \| 900 \| 2,3 \| 152,07 \| \|  \|  \| 6,8 \| 79 \| 18,7 \| 2,3 \| 48,85 \| 41,1317 \| 6,0574 \| 1,5632 \| 0,0977 \| 1200 \| 1,8 \| 213,73 \| \|  \|  \| 6,8 \| 79 \| 18,7 \| 2,3 \| 48,85 \| 41,1317 \| 6,0574 \| 1,5632 \| 0,0977 \| 1200 \| 2 \| 206,51 \| \|  \|  \| 6,8 \| 79 \| 18,7 \| 2,3 \| 48,85 \| 41,1317 \| 6,0574 \| 1,5632 \| 0,0977 \| 1200 \| 2,3 \| 197,68 \| \|  \|  \| 6,8 \| 79 \| 18,7 \| 2,3 \| 48,85 \| 41,1317 \| 6,0574 \| 1,5632 \| 0,0977 \| 1500 \| 1,8 \| 249,31 \| \|  \|  \| 6,8 \| 79 \| 18,7 \| 2,3 \| 48,85 \| 41,1317 \| 6,0574 \| 1,5632 \| 0,0977 \| 1500 \| 2 \| 246,46 \| \|  \|  \| 6,8 \| 79 \| 18,7 \| 2,3 \| 48,85 \| 41,1317 \| 6,0574 \| 1,5632 \| 0,0977 \| 1500 \| 2,3 \| 241,92 \| \| 58 \| Olive pits \| 6,1 \| 77 \| 19,9 \| 3,1 \| 51,1632 \| 38,1786 \| 6,3954 \| 1,0659 \| 0,0969 \| 600 \| 1,8 \| 123,05 \| \|  \|  \| 6,1 \| 77 \| 19,9 \| 3,1 \| 51,1632 \| 38,1786 \| 6,3954 \| 1,0659 \| 0,0969 \| 600 \| 2 \| 117,33 \| \|  \|  \| 6,1 \| 77 \| 19,9 \| 3,1 \| 51,1632 \| 38,1786 \| 6,3954 \| 1,0659 \| 0,0969 \| 600 \| 2,3 \| 110,86 \| \|  \|  \| 6,1 \| 77 \| 19,9 \| 3,1 \| 51,1632 \| 38,1786 \| 6,3954 \| 1,0659 \| 0,0969 \| 900 \| 1,8 \| 227,58 \| \|  \|  \| 6,1 \| 77 \| 19,9 \| 3,1 \| 51,1632 \| 38,1786 \| 6,3954 \| 1,0659 \| 0,0969 \| 900 \| 2 \| 219,00 \| \|  \|  \| 6,1 \| 77 \| 19,9 \| 3,1 \| 51,1632 \| 38,1786 \| 6,3954 \| 1,0659 \| 0,0969 \| 900 \| 2,3 \| 208,28 \| \|  \|  \| 6,1 \| 77 \| 19,9 \| 3,1 \| 51,1632 \| 38,1786 \| 6,3954 \| 1,0659 \| 0,0969 \| 1200 \| 1,8 \| 261,88 \| \|  \|  \| 6,1 \| 77 \| 19,9 \| 3,1 \| 51,1632 \| 38,1786 \| 6,3954 \| 1,0659 \| 0,0969 \| 1200 \| 2 \| 257,98 \| \|  \|  \| 6,1 \| 77 \| 19,9 \| 3,1 \| 51,1632 \| 38,1786 \| 6,3954 \| 1,0659 \| 0,0969 \| 1200 \| 2,3 \| 251,92 \| \|  \|  \| 6,1 \| 77 \| 19,9 \| 3,1 \| 51,1632 \| 38,1786 \| 6,3954 \| 1,0659 \| 0,0969 \| 1500 \| 1,8 \| 294,81 \| \|  \|  \| 6,1 \| 77 \| 19,9 \| 3,1 \| 51,1632 \| 38,1786 \| 6,3954 \| 1,0659 \| 0,0969 \| 1500 \| 2 \| 295,25 \| \|  \|  \| 6,1 \| 77 \| 19,9 \| 3,1 \| 51,1632 \| 38,1786 \| 6,3954 \| 1,0659 \| 0,0969 \| 1500 \| 2,3 \| 293,72 \| \| 59 \| Olive residue \| 10,6 \| 67,3 \| 25,5 \| 7,2 \| 54,1952 \| 31,7376 \| 5,3824 \| 1,2992 \| 0,1856 \| 600 \| 1,8 \| 127,10 \| \|  \|  \| 10,6 \| 67,3 \| 25,5 \| 7,2 \| 54,1952 \| 31,7376 \| 5,3824 \| 1,2992 \| 0,1856 \| 600 \| 2 \| 122,40 \| \|  \|  \| 10,6 \| 67,3 \| 25,5 \| 7,2 \| 54,1952 \| 31,7376 \| 5,3824 \| 1,2992 \| 0,1856 \| 600 \| 2,3 \| 116,73 \| \|  \|  \| 10,6 \| 67,3 \| 25,5 \| 7,2 \| 54,1952 \| 31,7376 \| 5,3824 \| 1,2992 \| 0,1856 \| 900 \| 1,8 \| 235,47 \| \|  \|  \| 10,6 \| 67,3 \| 25,5 \| 7,2 \| 54,1952 \| 31,7376 \| 5,3824 \| 1,2992 \| 0,1856 \| 900 \| 2 \| 229,85 \| \|  \|  \| 10,6 \| 67,3 \| 25,5 \| 7,2 \| 54,1952 \| 31,7376 \| 5,3824 \| 1,2992 \| 0,1856 \| 900 \| 2,3 \| 221,68 \| \|  \|  \| 10,6 \| 67,3 \| 25,5 \| 7,2 \| 54,1952 \| 31,7376 \| 5,3824 \| 1,2992 \| 0,1856 \| 1200 \| 1,8 \| 264,32 \| \|  \|  \| 10,6 \| 67,3 \| 25,5 \| 7,2 \| 54,1952 \| 31,7376 \| 5,3824 \| 1,2992 \| 0,1856 \| 1200 \| 2 \| 262,94 \| \|  \|  \| 10,6 \| 67,3 \| 25,5 \| 7,2 \| 54,1952 \| 31,7376 \| 5,3824 \| 1,2992 \| 0,1856 \| 1200 \| 2,3 \| 259,31 \| \|  \|  \| 10,6 \| 67,3 \| 25,5 \| 7,2 \| 54,1952 \| 31,7376 \| 5,3824 \| 1,2992 \| 0,1856 \| 1500 \| 1,8 \| 292,37 \| \|  \|  \| 10,6 \| 67,3 \| 25,5 \| 7,2 \| 54,1952 \| 31,7376 \| 5,3824 \| 1,2992 \| 0,1856 \| 1500 \| 2 \| 294,82 \| \|  \|  \| 10,6 \| 67,3 \| 25,5 \| 7,2 \| 54,1952 \| 31,7376 \| 5,3824 \| 1,2992 \| 0,1856 \| 1500 \| 2,3 \| 295,36 \| \| 60 \| Palm fibres-husks \| 36,4 \| 72,8 \| 18,9 \| 8,3 \| 47,2255 \| 36,7717 \| 6,0522 \| 1,3755 \| 0,2751 \| 600 \| 1,8 \| 53,90 \| \|  \|  \| 36,4 \| 72,8 \| 18,9 \| 8,3 \| 47,2255 \| 36,7717 \| 6,0522 \| 1,3755 \| 0,2751 \| 600 \| 2 \| 49,31 \| \|  \|  \| 36,4 \| 72,8 \| 18,9 \| 8,3 \| 47,2255 \| 36,7717 \| 6,0522 \| 1,3755 \| 0,2751 \| 600 \| 2,3 \| 44,38 \| \|  \|  \| 36,4 \| 72,8 \| 18,9 \| 8,3 \| 47,2255 \| 36,7717 \| 6,0522 \| 1,3755 \| 0,2751 \| 900 \| 1,8 \| 119,27 \| \|  \|  \| 36,4 \| 72,8 \| 18,9 \| 8,3 \| 47,2255 \| 36,7717 \| 6,0522 \| 1,3755 \| 0,2751 \| 900 \| 2 \| 111,37 \| \|  \|  \| 36,4 \| 72,8 \| 18,9 \| 8,3 \| 47,2255 \| 36,7717 \| 6,0522 \| 1,3755 \| 0,2751 \| 900 \| 2,3 \| 102,51 \| \|  \|  \| 36,4 \| 72,8 \| 18,9 \| 8,3 \| 47,2255 \| 36,7717 \| 6,0522 \| 1,3755 \| 0,2751 \| 1200 \| 1,8 \| 144,09 \| \|  \|  \| 36,4 \| 72,8 \| 18,9 \| 8,3 \| 47,2255 \| 36,7717 \| 6,0522 \| 1,3755 \| 0,2751 \| 1200 \| 2 \| 139,17 \| \|  \|  \| 36,4 \| 72,8 \| 18,9 \| 8,3 \| 47,2255 \| 36,7717 \| 6,0522 \| 1,3755 \| 0,2751 \| 1200 \| 2,3 \| 133,15 \| \|  \|  \| 36,4 \| 72,8 \| 18,9 \| 8,3 \| 47,2255 \| 36,7717 \| 6,0522 \| 1,3755 \| 0,2751 \| 1500 \| 1,8 \| 167,97 \| \|  \|  \| 36,4 \| 72,8 \| 18,9 \| 8,3 \| 47,2255 \| 36,7717 \| 6,0522 \| 1,3755 \| 0,2751 \| 1500 \| 2 \| 166,01 \| \|  \|  \| 36,4 \| 72,8 \| 18,9 \| 8,3 \| 47,2255 \| 36,7717 \| 6,0522 \| 1,3755 \| 0,2751 \| 1500 \| 2,3 \| 162,91 \| \| 61 \| Palm kernels \| 11 \| 77,3 \| 17,5 \| 5,2 \| 48,348 \| 37,446 \| 6,162 \| 2,5596 \| 0,2844 \| 600 \| 1,8 \| 87,84 \| \|  \|  \| 11 \| 77,3 \| 17,5 \| 5,2 \| 48,348 \| 37,446 \| 6,162 \| 2,5596 \| 0,2844 \| 600 \| 2 \| 81,62 \| \|  \|  \| 11 \| 77,3 \| 17,5 \| 5,2 \| 48,348 \| 37,446 \| 6,162 \| 2,5596 \| 0,2844 \| 600 \| 2,3 \| 74,88 \| \|  \|  \| 11 \| 77,3 \| 17,5 \| 5,2 \| 48,348 \| 37,446 \| 6,162 \| 2,5596 \| 0,2844 \| 900 \| 1,8 \| 181,65 \| \|  \|  \| 11 \| 77,3 \| 17,5 \| 5,2 \| 48,348 \| 37,446 \| 6,162 \| 2,5596 \| 0,2844 \| 900 \| 2 \| 171,23 \| \|  \|  \| 11 \| 77,3 \| 17,5 \| 5,2 \| 48,348 \| 37,446 \| 6,162 \| 2,5596 \| 0,2844 \| 900 \| 2,3 \| 159,30 \| \|  \|  \| 11 \| 77,3 \| 17,5 \| 5,2 \| 48,348 \| 37,446 \| 6,162 \| 2,5596 \| 0,2844 \| 1200 \| 1,8 \| 216,04 \| \|  \|  \| 11 \| 77,3 \| 17,5 \| 5,2 \| 48,348 \| 37,446 \| 6,162 \| 2,5596 \| 0,2844 \| 1200 \| 2 \| 209,91 \| \|  \|  \| 11 \| 77,3 \| 17,5 \| 5,2 \| 48,348 \| 37,446 \| 6,162 \| 2,5596 \| 0,2844 \| 1200 \| 2,3 \| 202,09 \| \|  \|  \| 11 \| 77,3 \| 17,5 \| 5,2 \| 48,348 \| 37,446 \| 6,162 \| 2,5596 \| 0,2844 \| 1500 \| 1,8 \| 249,07 \| \|  \|  \| 11 \| 77,3 \| 17,5 \| 5,2 \| 48,348 \| 37,446 \| 6,162 \| 2,5596 \| 0,2844 \| 1500 \| 2 \| 247,13 \| \|  \|  \| 11 \| 77,3 \| 17,5 \| 5,2 \| 48,348 \| 37,446 \| 6,162 \| 2,5596 \| 0,2844 \| 1500 \| 2,3 \| 243,49 \| \| 62 \| Pepper plant \| 6,5 \| 64,7 \| 20,9 \| 14,4 \| 36,1232 \| 41,944 \| 4,28 \| 2,7392 \| 0,5136 \| 600 \| 1,8 \| 0,00 \| \|  \|  \| 6,5 \| 64,7 \| 20,9 \| 14,4 \| 36,1232 \| 41,944 \| 4,28 \| 2,7392 \| 0,5136 \| 600 \| 2 \| 0,00 \| \|  \|  \| 6,5 \| 64,7 \| 20,9 \| 14,4 \| 36,1232 \| 41,944 \| 4,28 \| 2,7392 \| 0,5136 \| 600 \| 2,3 \| 0,00 \| \|  \|  \| 6,5 \| 64,7 \| 20,9 \| 14,4 \| 36,1232 \| 41,944 \| 4,28 \| 2,7392 \| 0,5136 \| 900 \| 1,8 \| 0,00 \| \|  \|  \| 6,5 \| 64,7 \| 20,9 \| 14,4 \| 36,1232 \| 41,944 \| 4,28 \| 2,7392 \| 0,5136 \| 900 \| 2 \| 0,00 \| \|  \|  \| 6,5 \| 64,7 \| 20,9 \| 14,4 \| 36,1232 \| 41,944 \| 4,28 \| 2,7392 \| 0,5136 \| 900 \| 2,3 \| 0,00 \| \|  \|  \| 6,5 \| 64,7 \| 20,9 \| 14,4 \| 36,1232 \| 41,944 \| 4,28 \| 2,7392 \| 0,5136 \| 1200 \| 1,8 \| 0,00 \| \|  \|  \| 6,5 \| 64,7 \| 20,9 \| 14,4 \| 36,1232 \| 41,944 \| 4,28 \| 2,7392 \| 0,5136 \| 1200 \| 2 \| 0,00 \| \|  \|  \| 6,5 \| 64,7 \| 20,9 \| 14,4 \| 36,1232 \| 41,944 \| 4,28 \| 2,7392 \| 0,5136 \| 1200 \| 2,3 \| 0,00 \| \|  \|  \| 6,5 \| 64,7 \| 20,9 \| 14,4 \| 36,1232 \| 41,944 \| 4,28 \| 2,7392 \| 0,5136 \| 1500 \| 1,8 \| 12,46 \| \|  \|  \| 6,5 \| 64,7 \| 20,9 \| 14,4 \| 36,1232 \| 41,944 \| 4,28 \| 2,7392 \| 0,5136 \| 1500 \| 2 \| 2,30 \| \|  \|  \| 6,5 \| 64,7 \| 20,9 \| 14,4 \| 36,1232 \| 41,944 \| 4,28 \| 2,7392 \| 0,5136 \| 1500 \| 2,3 \| 0,00 \| \| 63 \| Pepper residue \| 9,7 \| 64,8 \| 27 \| 8,2 \| 41,9526 \| 43,2378 \| 2,9376 \| 3,1212 \| 0,5508 \| 600 \| 1,8 \| 0,00 \| \|  \|  \| 9,7 \| 64,8 \| 27 \| 8,2 \| 41,9526 \| 43,2378 \| 2,9376 \| 3,1212 \| 0,5508 \| 600 \| 2 \| 0,00 \| \|  \|  \| 9,7 \| 64,8 \| 27 \| 8,2 \| 41,9526 \| 43,2378 \| 2,9376 \| 3,1212 \| 0,5508 \| 600 \| 2,3 \| 0,00 \| \|  \|  \| 9,7 \| 64,8 \| 27 \| 8,2 \| 41,9526 \| 43,2378 \| 2,9376 \| 3,1212 \| 0,5508 \| 900 \| 1,8 \| 0,00 \| \|  \|  \| 9,7 \| 64,8 \| 27 \| 8,2 \| 41,9526 \| 43,2378 \| 2,9376 \| 3,1212 \| 0,5508 \| 900 \| 2 \| 0,00 \| \|  \|  \| 9,7 \| 64,8 \| 27 \| 8,2 \| 41,9526 \| 43,2378 \| 2,9376 \| 3,1212 \| 0,5508 \| 900 \| 2,3 \| 0,00 \| \|  \|  \| 9,7 \| 64,8 \| 27 \| 8,2 \| 41,9526 \| 43,2378 \| 2,9376 \| 3,1212 \| 0,5508 \| 1200 \| 1,8 \| 0,00 \| \|  \|  \| 9,7 \| 64,8 \| 27 \| 8,2 \| 41,9526 \| 43,2378 \| 2,9376 \| 3,1212 \| 0,5508 \| 1200 \| 2 \| 0,00 \| \|  \|  \| 9,7 \| 64,8 \| 27 \| 8,2 \| 41,9526 \| 43,2378 \| 2,9376 \| 3,1212 \| 0,5508 \| 1200 \| 2,3 \| 0,00 \| \|  \|  \| 9,7 \| 64,8 \| 27 \| 8,2 \| 41,9526 \| 43,2378 \| 2,9376 \| 3,1212 \| 0,5508 \| 1500 \| 1,8 \| 26,31 \| \|  \|  \| 9,7 \| 64,8 \| 27 \| 8,2 \| 41,9526 \| 43,2378 \| 2,9376 \| 3,1212 \| 0,5508 \| 1500 \| 2 \| 16,57 \| \|  \|  \| 9,7 \| 64,8 \| 27 \| 8,2 \| 41,9526 \| 43,2378 \| 2,9376 \| 3,1212 \| 0,5508 \| 1500 \| 2,3 \| 6,30 \| \| 64 \| Pistachio shells \| 7,5 \| 81,6 \| 17 \| 1,4 \| 50,1874 \| 41,2148 \| 6,3104 \| 0,6902 \| 0,1972 \| 600 \| 1,8 \| 100,81 \| \|  \|  \| 7,5 \| 81,6 \| 17 \| 1,4 \| 50,1874 \| 41,2148 \| 6,3104 \| 0,6902 \| 0,1972 \| 600 \| 2 \| 94,60 \| \|  \|  \| 7,5 \| 81,6 \| 17 \| 1,4 \| 50,1874 \| 41,2148 \| 6,3104 \| 0,6902 \| 0,1972 \| 600 \| 2,3 \| 87,81 \| \|  \|  \| 7,5 \| 81,6 \| 17 \| 1,4 \| 50,1874 \| 41,2148 \| 6,3104 \| 0,6902 \| 0,1972 \| 900 \| 1,8 \| 199,39 \| \|  \|  \| 7,5 \| 81,6 \| 17 \| 1,4 \| 50,1874 \| 41,2148 \| 6,3104 \| 0,6902 \| 0,1972 \| 900 \| 2 \| 189,14 \| \|  \|  \| 7,5 \| 81,6 \| 17 \| 1,4 \| 50,1874 \| 41,2148 \| 6,3104 \| 0,6902 \| 0,1972 \| 900 \| 2,3 \| 177,20 \| \|  \|  \| 7,5 \| 81,6 \| 17 \| 1,4 \| 50,1874 \| 41,2148 \| 6,3104 \| 0,6902 \| 0,1972 \| 1200 \| 1,8 \| 235,09 \| \|  \|  \| 7,5 \| 81,6 \| 17 \| 1,4 \| 50,1874 \| 41,2148 \| 6,3104 \| 0,6902 \| 0,1972 \| 1200 \| 2 \| 229,38 \| \|  \|  \| 7,5 \| 81,6 \| 17 \| 1,4 \| 50,1874 \| 41,2148 \| 6,3104 \| 0,6902 \| 0,1972 \| 1200 \| 2,3 \| 221,80 \| \|  \|  \| 7,5 \| 81,6 \| 17 \| 1,4 \| 50,1874 \| 41,2148 \| 6,3104 \| 0,6902 \| 0,1972 \| 1500 \| 1,8 \| 269,28 \| \|  \|  \| 7,5 \| 81,6 \| 17 \| 1,4 \| 50,1874 \| 41,2148 \| 6,3104 \| 0,6902 \| 0,1972 \| 1500 \| 2 \| 267,95 \| \|  \|  \| 7,5 \| 81,6 \| 17 \| 1,4 \| 50,1874 \| 41,2148 \| 6,3104 \| 0,6902 \| 0,1972 \| 1500 \| 2,3 \| 264,78 \| \| 65 \| Plum pits \| 33,6 \| 80,8 \| 17,8 \| 1,4 \| 49,2014 \| 41,8064 \| 6,6062 \| 0,8874 \| 0,0986 \| 600 \| 1,8 \| 72,05 \| \|  \|  \| 33,6 \| 80,8 \| 17,8 \| 1,4 \| 49,2014 \| 41,8064 \| 6,6062 \| 0,8874 \| 0,0986 \| 600 \| 2 \| 67,54 \| \|  \|  \| 33,6 \| 80,8 \| 17,8 \| 1,4 \| 49,2014 \| 41,8064 \| 6,6062 \| 0,8874 \| 0,0986 \| 600 \| 2,3 \| 62,62 \| \|  \|  \| 33,6 \| 80,8 \| 17,8 \| 1,4 \| 49,2014 \| 41,8064 \| 6,6062 \| 0,8874 \| 0,0986 \| 900 \| 1,8 \| 141,05 \| \|  \|  \| 33,6 \| 80,8 \| 17,8 \| 1,4 \| 49,2014 \| 41,8064 \| 6,6062 \| 0,8874 \| 0,0986 \| 900 \| 2 \| 133,48 \| \|  \|  \| 33,6 \| 80,8 \| 17,8 \| 1,4 \| 49,2014 \| 41,8064 \| 6,6062 \| 0,8874 \| 0,0986 \| 900 \| 2,3 \| 124,75 \| \|  \|  \| 33,6 \| 80,8 \| 17,8 \| 1,4 \| 49,2014 \| 41,8064 \| 6,6062 \| 0,8874 \| 0,0986 \| 1200 \| 1,8 \| 167,21 \| \|  \|  \| 33,6 \| 80,8 \| 17,8 \| 1,4 \| 49,2014 \| 41,8064 \| 6,6062 \| 0,8874 \| 0,0986 \| 1200 \| 2 \| 162,87 \| \|  \|  \| 33,6 \| 80,8 \| 17,8 \| 1,4 \| 49,2014 \| 41,8064 \| 6,6062 \| 0,8874 \| 0,0986 \| 1200 \| 2,3 \| 157,25 \| \|  \|  \| 33,6 \| 80,8 \| 17,8 \| 1,4 \| 49,2014 \| 41,8064 \| 6,6062 \| 0,8874 \| 0,0986 \| 1500 \| 1,8 \| 192,25 \| \|  \|  \| 33,6 \| 80,8 \| 17,8 \| 1,4 \| 49,2014 \| 41,8064 \| 6,6062 \| 0,8874 \| 0,0986 \| 1500 \| 2 \| 191,06 \| \|  \|  \| 33,6 \| 80,8 \| 17,8 \| 1,4 \| 49,2014 \| 41,8064 \| 6,6062 \| 0,8874 \| 0,0986 \| 1500 \| 2,3 \| 188,58 \| \| 66 \| Rice husks \| 10,6 \| 62,8 \| 19,2 \| 18 \| 40,426 \| 35,834 \| 5,002 \| 0,656 \| 0,082 \| 600 \| 1,8 \| 0,00 \| \|  \|  \| 10,6 \| 62,8 \| 19,2 \| 18 \| 40,426 \| 35,834 \| 5,002 \| 0,656 \| 0,082 \| 600 \| 2 \| 0,00 \| \|  \|  \| 10,6 \| 62,8 \| 19,2 \| 18 \| 40,426 \| 35,834 \| 5,002 \| 0,656 \| 0,082 \| 600 \| 2,3 \| 0,00 \| \|  \|  \| 10,6 \| 62,8 \| 19,2 \| 18 \| 40,426 \| 35,834 \| 5,002 \| 0,656 \| 0,082 \| 900 \| 1,8 \| 47,30 \| \|  \|  \| 10,6 \| 62,8 \| 19,2 \| 18 \| 40,426 \| 35,834 \| 5,002 \| 0,656 \| 0,082 \| 900 \| 2 \| 32,71 \| \|  \|  \| 10,6 \| 62,8 \| 19,2 \| 18 \| 40,426 \| 35,834 \| 5,002 \| 0,656 \| 0,082 \| 900 \| 2,3 \| 17,50 \| \|  \|  \| 10,6 \| 62,8 \| 19,2 \| 18 \| 40,426 \| 35,834 \| 5,002 \| 0,656 \| 0,082 \| 1200 \| 1,8 \| 84,18 \| \|  \|  \| 10,6 \| 62,8 \| 19,2 \| 18 \| 40,426 \| 35,834 \| 5,002 \| 0,656 \| 0,082 \| 1200 \| 2 \| 73,04 \| \|  \|  \| 10,6 \| 62,8 \| 19,2 \| 18 \| 40,426 \| 35,834 \| 5,002 \| 0,656 \| 0,082 \| 1200 \| 2,3 \| 61,23 \| \|  \|  \| 10,6 \| 62,8 \| 19,2 \| 18 \| 40,426 \| 35,834 \| 5,002 \| 0,656 \| 0,082 \| 1500 \| 1,8 \| 120,47 \| \|  \|  \| 10,6 \| 62,8 \| 19,2 \| 18 \| 40,426 \| 35,834 \| 5,002 \| 0,656 \| 0,082 \| 1500 \| 2 \| 112,85 \| \|  \|  \| 10,6 \| 62,8 \| 19,2 \| 18 \| 40,426 \| 35,834 \| 5,002 \| 0,656 \| 0,082 \| 1500 \| 2,3 \| 104,49 \| \| 67 \| Soya husks \| 6,3 \| 74,3 \| 20,3 \| 5,4 \| 42,9484 \| 44,3674 \| 6,3382 \| 0,8514 \| 0,0946 \| 600 \| 1,8 \| 24,79 \| \|  \|  \| 6,3 \| 74,3 \| 20,3 \| 5,4 \| 42,9484 \| 44,3674 \| 6,3382 \| 0,8514 \| 0,0946 \| 600 \| 2 \| 17,09 \| \|  \|  \| 6,3 \| 74,3 \| 20,3 \| 5,4 \| 42,9484 \| 44,3674 \| 6,3382 \| 0,8514 \| 0,0946 \| 600 \| 2,3 \| 8,99 \| \|  \|  \| 6,3 \| 74,3 \| 20,3 \| 5,4 \| 42,9484 \| 44,3674 \| 6,3382 \| 0,8514 \| 0,0946 \| 900 \| 1,8 \| 100,07 \| \|  \|  \| 6,3 \| 74,3 \| 20,3 \| 5,4 \| 42,9484 \| 44,3674 \| 6,3382 \| 0,8514 \| 0,0946 \| 900 \| 2 \| 85,80 \| \|  \|  \| 6,3 \| 74,3 \| 20,3 \| 5,4 \| 42,9484 \| 44,3674 \| 6,3382 \| 0,8514 \| 0,0946 \| 900 \| 2,3 \| 70,80 \| \|  \|  \| 6,3 \| 74,3 \| 20,3 \| 5,4 \| 42,9484 \| 44,3674 \| 6,3382 \| 0,8514 \| 0,0946 \| 1200 \| 1,8 \| 140,92 \| \|  \|  \| 6,3 \| 74,3 \| 20,3 \| 5,4 \| 42,9484 \| 44,3674 \| 6,3382 \| 0,8514 \| 0,0946 \| 1200 \| 2 \| 130,42 \| \|  \|  \| 6,3 \| 74,3 \| 20,3 \| 5,4 \| 42,9484 \| 44,3674 \| 6,3382 \| 0,8514 \| 0,0946 \| 1200 \| 2,3 \| 119,05 \| \|  \|  \| 6,3 \| 74,3 \| 20,3 \| 5,4 \| 42,9484 \| 44,3674 \| 6,3382 \| 0,8514 \| 0,0946 \| 1500 \| 1,8 \| 180,74 \| \|  \|  \| 6,3 \| 74,3 \| 20,3 \| 5,4 \| 42,9484 \| 44,3674 \| 6,3382 \| 0,8514 \| 0,0946 \| 1500 \| 2 \| 174,14 \| \|  \|  \| 6,3 \| 74,3 \| 20,3 \| 5,4 \| 42,9484 \| 44,3674 \| 6,3382 \| 0,8514 \| 0,0946 \| 1500 \| 2,3 \| 166,54 \| \| 68 \| Sugar cane bagasse \| 10,4 \| 85,5 \| 12,4 \| 2,1 \| 48,7542 \| 42,9781 \| 5,874 \| 0,1958 \| 0,0979 \| 600 \| 1,8 \| 64,37 \| \|  \|  \| 10,4 \| 85,5 \| 12,4 \| 2,1 \| 48,7542 \| 42,9781 \| 5,874 \| 0,1958 \| 0,0979 \| 600 \| 2 \| 57,72 \| \|  \|  \| 10,4 \| 85,5 \| 12,4 \| 2,1 \| 48,7542 \| 42,9781 \| 5,874 \| 0,1958 \| 0,0979 \| 600 \| 2,3 \| 50,62 \| \|  \|  \| 10,4 \| 85,5 \| 12,4 \| 2,1 \| 48,7542 \| 42,9781 \| 5,874 \| 0,1958 \| 0,0979 \| 900 \| 1,8 \| 154,06 \| \|  \|  \| 10,4 \| 85,5 \| 12,4 \| 2,1 \| 48,7542 \| 42,9781 \| 5,874 \| 0,1958 \| 0,0979 \| 900 \| 2 \| 142,22 \| \|  \|  \| 10,4 \| 85,5 \| 12,4 \| 2,1 \| 48,7542 \| 42,9781 \| 5,874 \| 0,1958 \| 0,0979 \| 900 \| 2,3 \| 129,21 \| \|  \|  \| 10,4 \| 85,5 \| 12,4 \| 2,1 \| 48,7542 \| 42,9781 \| 5,874 \| 0,1958 \| 0,0979 \| 1200 \| 1,8 \| 190,28 \| \|  \|  \| 10,4 \| 85,5 \| 12,4 \| 2,1 \| 48,7542 \| 42,9781 \| 5,874 \| 0,1958 \| 0,0979 \| 1200 \| 2 \| 182,55 \| \|  \|  \| 10,4 \| 85,5 \| 12,4 \| 2,1 \| 48,7542 \| 42,9781 \| 5,874 \| 0,1958 \| 0,0979 \| 1200 \| 2,3 \| 173,42 \| \|  \|  \| 10,4 \| 85,5 \| 12,4 \| 2,1 \| 48,7542 \| 42,9781 \| 5,874 \| 0,1958 \| 0,0979 \| 1500 \| 1,8 \| 225,13 \| \|  \|  \| 10,4 \| 85,5 \| 12,4 \| 2,1 \| 48,7542 \| 42,9781 \| 5,874 \| 0,1958 \| 0,0979 \| 1500 \| 2 \| 221,55 \| \|  \|  \| 10,4 \| 85,5 \| 12,4 \| 2,1 \| 48,7542 \| 42,9781 \| 5,874 \| 0,1958 \| 0,0979 \| 1500 \| 2,3 \| 216,45 \| \| 69 \| Sunflower husks \| 9,1 \| 76 \| 20,9 \| 3,1 \| 48,8376 \| 41,667 \| 5,3295 \| 1,0659 \| 0 \| 600 \| 1,8 \| 51,01 \| \|  \|  \| 9,1 \| 76 \| 20,9 \| 3,1 \| 48,8376 \| 41,667 \| 5,3295 \| 1,0659 \| 0 \| 600 \| 2 \| 44,06 \| \|  \|  \| 9,1 \| 76 \| 20,9 \| 3,1 \| 48,8376 \| 41,667 \| 5,3295 \| 1,0659 \| 0 \| 600 \| 2,3 \| 36,68 \| \|  \|  \| 9,1 \| 76 \| 20,9 \| 3,1 \| 48,8376 \| 41,667 \| 5,3295 \| 1,0659 \| 0 \| 900 \| 1,8 \| 142,52 \| \|  \|  \| 9,1 \| 76 \| 20,9 \| 3,1 \| 48,8376 \| 41,667 \| 5,3295 \| 1,0659 \| 0 \| 900 \| 2 \| 130,03 \| \|  \|  \| 9,1 \| 76 \| 20,9 \| 3,1 \| 48,8376 \| 41,667 \| 5,3295 \| 1,0659 \| 0 \| 900 \| 2,3 \| 116,45 \| \|  \|  \| 9,1 \| 76 \| 20,9 \| 3,1 \| 48,8376 \| 41,667 \| 5,3295 \| 1,0659 \| 0 \| 1200 \| 1,8 \| 179,00 \| \|  \|  \| 9,1 \| 76 \| 20,9 \| 3,1 \| 48,8376 \| 41,667 \| 5,3295 \| 1,0659 \| 0 \| 1200 \| 2 \| 170,58 \| \|  \|  \| 9,1 \| 76 \| 20,9 \| 3,1 \| 48,8376 \| 41,667 \| 5,3295 \| 1,0659 \| 0 \| 1200 \| 2,3 \| 160,86 \| \|  \|  \| 9,1 \| 76 \| 20,9 \| 3,1 \| 48,8376 \| 41,667 \| 5,3295 \| 1,0659 \| 0 \| 1500 \| 1,8 \| 214,22 \| \|  \|  \| 9,1 \| 76 \| 20,9 \| 3,1 \| 48,8376 \| 41,667 \| 5,3295 \| 1,0659 \| 0 \| 1500 \| 2 \| 209,94 \| \|  \|  \| 9,1 \| 76 \| 20,9 \| 3,1 \| 48,8376 \| 41,667 \| 5,3295 \| 1,0659 \| 0 \| 1500 \| 2,3 \| 204,22 \| \| 70 \| Walnut blows \| 23,5 \| 80,7 \| 16,9 \| 2,4 \| 53,5824 \| 36,0144 \| 6,5392 \| 1,3664 \| 0,0976 \| 600 \| 1,8 \| 126,43 \| \|  \|  \| 23,5 \| 80,7 \| 16,9 \| 2,4 \| 53,5824 \| 36,0144 \| 6,5392 \| 1,3664 \| 0,0976 \| 600 \| 2 \| 122,86 \| \|  \|  \| 23,5 \| 80,7 \| 16,9 \| 2,4 \| 53,5824 \| 36,0144 \| 6,5392 \| 1,3664 \| 0,0976 \| 600 \| 2,3 \| 118,35 \| \|  \|  \| 23,5 \| 80,7 \| 16,9 \| 2,4 \| 53,5824 \| 36,0144 \| 6,5392 \| 1,3664 \| 0,0976 \| 900 \| 1,8 \| 215,26 \| \|  \|  \| 23,5 \| 80,7 \| 16,9 \| 2,4 \| 53,5824 \| 36,0144 \| 6,5392 \| 1,3664 \| 0,0976 \| 900 \| 2 \| 211,03 \| \|  \|  \| 23,5 \| 80,7 \| 16,9 \| 2,4 \| 53,5824 \| 36,0144 \| 6,5392 \| 1,3664 \| 0,0976 \| 900 \| 2,3 \| 204,60 \| \|  \|  \| 23,5 \| 80,7 \| 16,9 \| 2,4 \| 53,5824 \| 36,0144 \| 6,5392 \| 1,3664 \| 0,0976 \| 1200 \| 1,8 \| 241,09 \| \|  \|  \| 23,5 \| 80,7 \| 16,9 \| 2,4 \| 53,5824 \| 36,0144 \| 6,5392 \| 1,3664 \| 0,0976 \| 1200 \| 2 \| 240,49 \| \|  \|  \| 23,5 \| 80,7 \| 16,9 \| 2,4 \| 53,5824 \| 36,0144 \| 6,5392 \| 1,3664 \| 0,0976 \| 1200 \| 2,3 \| 237,96 \| \|  \|  \| 23,5 \| 80,7 \| 16,9 \| 2,4 \| 53,5824 \| 36,0144 \| 6,5392 \| 1,3664 \| 0,0976 \| 1500 \| 1,8 \| 266,14 \| \|  \|  \| 23,5 \| 80,7 \| 16,9 \| 2,4 \| 53,5824 \| 36,0144 \| 6,5392 \| 1,3664 \| 0,0976 \| 1500 \| 2 \| 268,75 \| \|  \|  \| 23,5 \| 80,7 \| 16,9 \| 2,4 \| 53,5824 \| 36,0144 \| 6,5392 \| 1,3664 \| 0,0976 \| 1500 \| 2,3 \| 269,78 \| \| 71 \| Walnut hulls and blows \| 47,9 \| 79,6 \| 17,5 \| 2,9 \| 53,5021 \| 35,4415 \| 6,5057 \| 1,5536 \| 0,0971 \| 600 \| 1,8 \| 86,03 \| \|  \|  \| 47,9 \| 79,6 \| 17,5 \| 2,9 \| 53,5021 \| 35,4415 \| 6,5057 \| 1,5536 \| 0,0971 \| 600 \| 2 \| 83,60 \| \|  \|  \| 47,9 \| 79,6 \| 17,5 \| 2,9 \| 53,5021 \| 35,4415 \| 6,5057 \| 1,5536 \| 0,0971 \| 600 \| 2,3 \| 80,53 \| \|  \|  \| 47,9 \| 79,6 \| 17,5 \| 2,9 \| 53,5021 \| 35,4415 \| 6,5057 \| 1,5536 \| 0,0971 \| 900 \| 1,8 \| 146,66 \| \|  \|  \| 47,9 \| 79,6 \| 17,5 \| 2,9 \| 53,5021 \| 35,4415 \| 6,5057 \| 1,5536 \| 0,0971 \| 900 \| 2 \| 143,80 \| \|  \|  \| 47,9 \| 79,6 \| 17,5 \| 2,9 \| 53,5021 \| 35,4415 \| 6,5057 \| 1,5536 \| 0,0971 \| 900 \| 2,3 \| 139,44 \| \|  \|  \| 47,9 \| 79,6 \| 17,5 \| 2,9 \| 53,5021 \| 35,4415 \| 6,5057 \| 1,5536 \| 0,0971 \| 1200 \| 1,8 \| 164,18 \| \|  \|  \| 47,9 \| 79,6 \| 17,5 \| 2,9 \| 53,5021 \| 35,4415 \| 6,5057 \| 1,5536 \| 0,0971 \| 1200 \| 2 \| 163,79 \| \|  \|  \| 47,9 \| 79,6 \| 17,5 \| 2,9 \| 53,5021 \| 35,4415 \| 6,5057 \| 1,5536 \| 0,0971 \| 1200 \| 2,3 \| 162,09 \| \|  \|  \| 47,9 \| 79,6 \| 17,5 \| 2,9 \| 53,5021 \| 35,4415 \| 6,5057 \| 1,5536 \| 0,0971 \| 1500 \| 1,8 \| 181,19 \| \|  \|  \| 47,9 \| 79,6 \| 17,5 \| 2,9 \| 53,5021 \| 35,4415 \| 6,5057 \| 1,5536 \| 0,0971 \| 1500 \| 2 \| 182,98 \| \|  \|  \| 47,9 \| 79,6 \| 17,5 \| 2,9 \| 53,5021 \| 35,4415 \| 6,5057 \| 1,5536 \| 0,0971 \| 1500 \| 2,3 \| 183,69 \| \| 72 \| Walnut shells \| 6,8 \| 59,3 \| 37,9 \| 2,8 \| 48,5028 \| 41,2128 \| 6,0264 \| 1,3608 \| 0,0972 \| 600 \| 1,8 \| 76,10 \| \|  \|  \| 6,8 \| 59,3 \| 37,9 \| 2,8 \| 48,5028 \| 41,2128 \| 6,0264 \| 1,3608 \| 0,0972 \| 600 \| 2 \| 69,30 \| \|  \|  \| 6,8 \| 59,3 \| 37,9 \| 2,8 \| 48,5028 \| 41,2128 \| 6,0264 \| 1,3608 \| 0,0972 \| 600 \| 2,3 \| 62,02 \| \|  \|  \| 6,8 \| 59,3 \| 37,9 \| 2,8 \| 48,5028 \| 41,2128 \| 6,0264 \| 1,3608 \| 0,0972 \| 900 \| 1,8 \| 170,91 \| \|  \|  \| 6,8 \| 59,3 \| 37,9 \| 2,8 \| 48,5028 \| 41,2128 \| 6,0264 \| 1,3608 \| 0,0972 \| 900 \| 2 \| 159,02 \| \|  \|  \| 6,8 \| 59,3 \| 37,9 \| 2,8 \| 48,5028 \| 41,2128 \| 6,0264 \| 1,3608 \| 0,0972 \| 900 \| 2,3 \| 145,80 \| \|  \|  \| 6,8 \| 59,3 \| 37,9 \| 2,8 \| 48,5028 \| 41,2128 \| 6,0264 \| 1,3608 \| 0,0972 \| 1200 \| 1,8 \| 208,18 \| \|  \|  \| 6,8 \| 59,3 \| 37,9 \| 2,8 \| 48,5028 \| 41,2128 \| 6,0264 \| 1,3608 \| 0,0972 \| 1200 \| 2 \| 200,64 \| \|  \|  \| 6,8 \| 59,3 \| 37,9 \| 2,8 \| 48,5028 \| 41,2128 \| 6,0264 \| 1,3608 \| 0,0972 \| 1200 \| 2,3 \| 191,55 \| \|  \|  \| 6,8 \| 59,3 \| 37,9 \| 2,8 \| 48,5028 \| 41,2128 \| 6,0264 \| 1,3608 \| 0,0972 \| 1500 \| 1,8 \| 244,00 \| \|  \|  \| 6,8 \| 59,3 \| 37,9 \| 2,8 \| 48,5028 \| 41,2128 \| 6,0264 \| 1,3608 \| 0,0972 \| 1500 \| 2 \| 240,82 \| \|  \|  \| 6,8 \| 59,3 \| 37,9 \| 2,8 \| 48,5028 \| 41,2128 \| 6,0264 \| 1,3608 \| 0,0972 \| 1500 \| 2,3 \| 235,98 \| \| 73 \| Chicken litter \| 9,3 \| 47,8 \| 14,4 \| 37,8 \| 37,631 \| 15,7366 \| 4,2296 \| 3,8564 \| 0,7464 \| 600 \| 1,8 \| 0,00 \| \|  \|  \| 9,3 \| 47,8 \| 14,4 \| 37,8 \| 37,631 \| 15,7366 \| 4,2296 \| 3,8564 \| 0,7464 \| 600 \| 2 \| 0,00 \| \|  \|  \| 9,3 \| 47,8 \| 14,4 \| 37,8 \| 37,631 \| 15,7366 \| 4,2296 \| 3,8564 \| 0,7464 \| 600 \| 2,3 \| 0,00 \| \|  \|  \| 9,3 \| 47,8 \| 14,4 \| 37,8 \| 37,631 \| 15,7366 \| 4,2296 \| 3,8564 \| 0,7464 \| 900 \| 1,8 \| 72,47 \| \|  \|  \| 9,3 \| 47,8 \| 14,4 \| 37,8 \| 37,631 \| 15,7366 \| 4,2296 \| 3,8564 \| 0,7464 \| 900 \| 2 \| 58,14 \| \|  \|  \| 9,3 \| 47,8 \| 14,4 \| 37,8 \| 37,631 \| 15,7366 \| 4,2296 \| 3,8564 \| 0,7464 \| 900 \| 2,3 \| 43,06 \| \|  \|  \| 9,3 \| 47,8 \| 14,4 \| 37,8 \| 37,631 \| 15,7366 \| 4,2296 \| 3,8564 \| 0,7464 \| 1200 \| 1,8 \| 105,49 \| \|  \|  \| 9,3 \| 47,8 \| 14,4 \| 37,8 \| 37,631 \| 15,7366 \| 4,2296 \| 3,8564 \| 0,7464 \| 1200 \| 2 \| 94,88 \| \|  \|  \| 9,3 \| 47,8 \| 14,4 \| 37,8 \| 37,631 \| 15,7366 \| 4,2296 \| 3,8564 \| 0,7464 \| 1200 \| 2,3 \| 83,41 \| \|  \|  \| 9,3 \| 47,8 \| 14,4 \| 37,8 \| 37,631 \| 15,7366 \| 4,2296 \| 3,8564 \| 0,7464 \| 1500 \| 1,8 \| 138,16 \| \|  \|  \| 9,3 \| 47,8 \| 14,4 \| 37,8 \| 37,631 \| 15,7366 \| 4,2296 \| 3,8564 \| 0,7464 \| 1500 \| 2 \| 131,29 \| \|  \|  \| 9,3 \| 47,8 \| 14,4 \| 37,8 \| 37,631 \| 15,7366 \| 4,2296 \| 3,8564 \| 0,7464 \| 1500 \| 2,3 \| 123,48 \| \| 74 \| Meat-bone meal \| 2,5 \| 63,3 \| 12,7 \| 24 \| 43,548 \| 15,808 \| 6,08 \| 9,272 \| 1,292 \| 600 \| 1,8 \| 99,31 \| \|  \|  \| 2,5 \| 63,3 \| 12,7 \| 24 \| 43,548 \| 15,808 \| 6,08 \| 9,272 \| 1,292 \| 600 \| 2 \| 92,42 \| \|  \|  \| 2,5 \| 63,3 \| 12,7 \| 24 \| 43,548 \| 15,808 \| 6,08 \| 9,272 \| 1,292 \| 600 \| 2,3 \| 84,89 \| \|  \|  \| 2,5 \| 63,3 \| 12,7 \| 24 \| 43,548 \| 15,808 \| 6,08 \| 9,272 \| 1,292 \| 900 \| 1,8 \| 206,51 \| \|  \|  \| 2,5 \| 63,3 \| 12,7 \| 24 \| 43,548 \| 15,808 \| 6,08 \| 9,272 \| 1,292 \| 900 \| 2 \| 195,91 \| \|  \|  \| 2,5 \| 63,3 \| 12,7 \| 24 \| 43,548 \| 15,808 \| 6,08 \| 9,272 \| 1,292 \| 900 \| 2,3 \| 183,36 \| \|  \|  \| 2,5 \| 63,3 \| 12,7 \| 24 \| 43,548 \| 15,808 \| 6,08 \| 9,272 \| 1,292 \| 1200 \| 1,8 \| 240,32 \| \|  \|  \| 2,5 \| 63,3 \| 12,7 \| 24 \| 43,548 \| 15,808 \| 6,08 \| 9,272 \| 1,292 \| 1200 \| 2 \| 234,37 \| \|  \|  \| 2,5 \| 63,3 \| 12,7 \| 24 \| 43,548 \| 15,808 \| 6,08 \| 9,272 \| 1,292 \| 1200 \| 2,3 \| 226,42 \| \|  \|  \| 2,5 \| 63,3 \| 12,7 \| 24 \| 43,548 \| 15,808 \| 6,08 \| 9,272 \| 1,292 \| 1500 \| 1,8 \| 273,19 \| \|  \|  \| 2,5 \| 63,3 \| 12,7 \| 24 \| 43,548 \| 15,808 \| 6,08 \| 9,272 \| 1,292 \| 1500 \| 2 \| 271,72 \| \|  \|  \| 2,5 \| 63,3 \| 12,7 \| 24 \| 43,548 \| 15,808 \| 6,08 \| 9,272 \| 1,292 \| 1500 \| 2,3 \| 268,33 \| \| 75 \| Biomass mixture \| 8,8 \| 69,4 \| 18,1 \| 12,5 \| 49,6125 \| 28,9625 \| 5,775 \| 2,3625 \| 0,7875 \| 600 \| 1,8 \| 113,40 \| \|  \|  \| 8,8 \| 69,4 \| 18,1 \| 12,5 \| 49,6125 \| 28,9625 \| 5,775 \| 2,3625 \| 0,7875 \| 600 \| 2 \| 107,77 \| \|  \|  \| 8,8 \| 69,4 \| 18,1 \| 12,5 \| 49,6125 \| 28,9625 \| 5,775 \| 2,3625 \| 0,7875 \| 600 \| 2,3 \| 101,39 \| \|  \|  \| 8,8 \| 69,4 \| 18,1 \| 12,5 \| 49,6125 \| 28,9625 \| 5,775 \| 2,3625 \| 0,7875 \| 900 \| 1,8 \| 218,17 \| \|  \|  \| 8,8 \| 69,4 \| 18,1 \| 12,5 \| 49,6125 \| 28,9625 \| 5,775 \| 2,3625 \| 0,7875 \| 900 \| 2 \| 210,15 \| \|  \|  \| 8,8 \| 69,4 \| 18,1 \| 12,5 \| 49,6125 \| 28,9625 \| 5,775 \| 2,3625 \| 0,7875 \| 900 \| 2,3 \| 199,90 \| \|  \|  \| 8,8 \| 69,4 \| 18,1 \| 12,5 \| 49,6125 \| 28,9625 \| 5,775 \| 2,3625 \| 0,7875 \| 1200 \| 1,8 \| 249,39 \| \|  \|  \| 8,8 \| 69,4 \| 18,1 \| 12,5 \| 49,6125 \| 28,9625 \| 5,775 \| 2,3625 \| 0,7875 \| 1200 \| 2 \| 245,86 \| \|  \|  \| 8,8 \| 69,4 \| 18,1 \| 12,5 \| 49,6125 \| 28,9625 \| 5,775 \| 2,3625 \| 0,7875 \| 1200 \| 2,3 \| 240,16 \| \|  \|  \| 8,8 \| 69,4 \| 18,1 \| 12,5 \| 49,6125 \| 28,9625 \| 5,775 \| 2,3625 \| 0,7875 \| 1500 \| 1,8 \| 279,58 \| \|  \|  \| 8,8 \| 69,4 \| 18,1 \| 12,5 \| 49,6125 \| 28,9625 \| 5,775 \| 2,3625 \| 0,7875 \| 1500 \| 2 \| 280,21 \| \|  \|  \| 8,8 \| 69,4 \| 18,1 \| 12,5 \| 49,6125 \| 28,9625 \| 5,775 \| 2,3625 \| 0,7875 \| 1500 \| 2,3 \| 278,89 \| \| 76 \| Wood-agricultural residue \| 30,3 \| 78,5 \| 18,2 \| 3,3 \| 50,6708 \| 39,8404 \| 5,802 \| 0,3868 \| 0 \| 600 \| 1,8 \| 57,20 \| \|  \|  \| 30,3 \| 78,5 \| 18,2 \| 3,3 \| 50,6708 \| 39,8404 \| 5,802 \| 0,3868 \| 0 \| 600 \| 2 \| 49,78 \| \|  \|  \| 30,3 \| 78,5 \| 18,2 \| 3,3 \| 50,6708 \| 39,8404 \| 5,802 \| 0,3868 \| 0 \| 600 \| 2,3 \| 41,90 \| \|  \|  \| 30,3 \| 78,5 \| 18,2 \| 3,3 \| 50,6708 \| 39,8404 \| 5,802 \| 0,3868 \| 0 \| 900 \| 1,8 \| 124,89 \| \|  \|  \| 30,3 \| 78,5 \| 18,2 \| 3,3 \| 50,6708 \| 39,8404 \| 5,802 \| 0,3868 \| 0 \| 900 \| 2 \| 111,30 \| \|  \|  \| 30,3 \| 78,5 \| 18,2 \| 3,3 \| 50,6708 \| 39,8404 \| 5,802 \| 0,3868 \| 0 \| 900 \| 2,3 \| 96,70 \| \|  \|  \| 30,3 \| 78,5 \| 18,2 \| 3,3 \| 50,6708 \| 39,8404 \| 5,802 \| 0,3868 \| 0 \| 1200 \| 1,8 \| 158,69 \| \|  \|  \| 30,3 \| 78,5 \| 18,2 \| 3,3 \| 50,6708 \| 39,8404 \| 5,802 \| 0,3868 \| 0 \| 1200 \| 2 \| 149,06 \| \|  \|  \| 30,3 \| 78,5 \| 18,2 \| 3,3 \| 50,6708 \| 39,8404 \| 5,802 \| 0,3868 \| 0 \| 1200 \| 2,3 \| 138,15 \| \|  \|  \| 30,3 \| 78,5 \| 18,2 \| 3,3 \| 50,6708 \| 39,8404 \| 5,802 \| 0,3868 \| 0 \| 1500 \| 1,8 \| 191,37 \| \|  \|  \| 30,3 \| 78,5 \| 18,2 \| 3,3 \| 50,6708 \| 39,8404 \| 5,802 \| 0,3868 \| 0 \| 1500 \| 2 \| 185,86 \| \|  \|  \| 30,3 \| 78,5 \| 18,2 \| 3,3 \| 50,6708 \| 39,8404 \| 5,802 \| 0,3868 \| 0 \| 1500 \| 2,3 \| 178,84 \| \| 77 \| Wood-almond residue \| 22,7 \| 77,2 \| 15,9 \| 6,9 \| 47,3879 \| 39,5675 \| 5,4929 \| 0,5586 \| 0,0931 \| 600 \| 1,8 \| 42,23 \| \|  \|  \| 22,7 \| 77,2 \| 15,9 \| 6,9 \| 47,3879 \| 39,5675 \| 5,4929 \| 0,5586 \| 0,0931 \| 600 \| 2 \| 36,28 \| \|  \|  \| 22,7 \| 77,2 \| 15,9 \| 6,9 \| 47,3879 \| 39,5675 \| 5,4929 \| 0,5586 \| 0,0931 \| 600 \| 2,3 \| 29,96 \| \|  \|  \| 22,7 \| 77,2 \| 15,9 \| 6,9 \| 47,3879 \| 39,5675 \| 5,4929 \| 0,5586 \| 0,0931 \| 900 \| 1,8 \| 118,69 \| \|  \|  \| 22,7 \| 77,2 \| 15,9 \| 6,9 \| 47,3879 \| 39,5675 \| 5,4929 \| 0,5586 \| 0,0931 \| 900 \| 2 \| 108,00 \| \|  \|  \| 22,7 \| 77,2 \| 15,9 \| 6,9 \| 47,3879 \| 39,5675 \| 5,4929 \| 0,5586 \| 0,0931 \| 900 \| 2,3 \| 96,41 \| \|  \|  \| 22,7 \| 77,2 \| 15,9 \| 6,9 \| 47,3879 \| 39,5675 \| 5,4929 \| 0,5586 \| 0,0931 \| 1200 \| 1,8 \| 149,64 \| \|  \|  \| 22,7 \| 77,2 \| 15,9 \| 6,9 \| 47,3879 \| 39,5675 \| 5,4929 \| 0,5586 \| 0,0931 \| 1200 \| 2 \| 142,40 \| \|  \|  \| 22,7 \| 77,2 \| 15,9 \| 6,9 \| 47,3879 \| 39,5675 \| 5,4929 \| 0,5586 \| 0,0931 \| 1200 \| 2,3 \| 134,07 \| \|  \|  \| 22,7 \| 77,2 \| 15,9 \| 6,9 \| 47,3879 \| 39,5675 \| 5,4929 \| 0,5586 \| 0,0931 \| 1500 \| 1,8 \| 179,57 \| \|  \|  \| 22,7 \| 77,2 \| 15,9 \| 6,9 \| 47,3879 \| 39,5675 \| 5,4929 \| 0,5586 \| 0,0931 \| 1500 \| 2 \| 175,82 \| \|  \|  \| 22,7 \| 77,2 \| 15,9 \| 6,9 \| 47,3879 \| 39,5675 \| 5,4929 \| 0,5586 \| 0,0931 \| 1500 \| 2,3 \| 170,87 \| \| 78 \| Wood-straw residue \| 7,3 \| 75,51 \| 16,7 \| 8,2 \| 47,4606 \| 38,097 \| 5,7834 \| 0,3672 \| 0,0918 \| 600 \| 1,8 \| 73,57 \| \|  \|  \| 7,3 \| 75,51 \| 16,7 \| 8,2 \| 47,4606 \| 38,097 \| 5,7834 \| 0,3672 \| 0,0918 \| 600 \| 2 \| 68,47 \| \|  \|  \| 7,3 \| 75,51 \| 16,7 \| 8,2 \| 47,4606 \| 38,097 \| 5,7834 \| 0,3672 \| 0,0918 \| 600 \| 2,3 \| 62,92 \| \|  \|  \| 7,3 \| 75,51 \| 16,7 \| 8,2 \| 47,4606 \| 38,097 \| 5,7834 \| 0,3672 \| 0,0918 \| 900 \| 1,8 \| 172,40 \| \|  \|  \| 7,3 \| 75,51 \| 16,7 \| 8,2 \| 47,4606 \| 38,097 \| 5,7834 \| 0,3672 \| 0,0918 \| 900 \| 2 \| 164,21 \| \|  \|  \| 7,3 \| 75,51 \| 16,7 \| 8,2 \| 47,4606 \| 38,097 \| 5,7834 \| 0,3672 \| 0,0918 \| 900 \| 2,3 \| 154,69 \| \|  \|  \| 7,3 \| 75,51 \| 16,7 \| 8,2 \| 47,4606 \| 38,097 \| 5,7834 \| 0,3672 \| 0,0918 \| 1200 \| 1,8 \| 203,63 \| \|  \|  \| 7,3 \| 75,51 \| 16,7 \| 8,2 \| 47,4606 \| 38,097 \| 5,7834 \| 0,3672 \| 0,0918 \| 1200 \| 2 \| 199,12 \| \|  \|  \| 7,3 \| 75,51 \| 16,7 \| 8,2 \| 47,4606 \| 38,097 \| 5,7834 \| 0,3672 \| 0,0918 \| 1200 \| 2,3 \| 193,23 \| \|  \|  \| 7,3 \| 75,51 \| 16,7 \| 8,2 \| 47,4606 \| 38,097 \| 5,7834 \| 0,3672 \| 0,0918 \| 1500 \| 1,8 \| 233,73 \| \|  \|  \| 7,3 \| 75,51 \| 16,7 \| 8,2 \| 47,4606 \| 38,097 \| 5,7834 \| 0,3672 \| 0,0918 \| 1500 \| 2 \| 232,75 \| \|  \|  \| 7,3 \| 75,51 \| 16,7 \| 8,2 \| 47,4606 \| 38,097 \| 5,7834 \| 0,3672 \| 0,0918 \| 1500 \| 2,3 \| 230,44 \| \| 79 \| Currency shredded \| 4,7 \| 82,9 \| 11,6 \| 5,5 \| 42,903 \| 43,5645 \| 5,9535 \| 1,7955 \| 0,2835 \| 600 \| 1,8 \| 17,04 \| \|  \|  \| 4,7 \| 82,9 \| 11,6 \| 5,5 \| 42,903 \| 43,5645 \| 5,9535 \| 1,7955 \| 0,2835 \| 600 \| 2 \| 11,00 \| \|  \|  \| 4,7 \| 82,9 \| 11,6 \| 5,5 \| 42,903 \| 43,5645 \| 5,9535 \| 1,7955 \| 0,2835 \| 600 \| 2,3 \| 4,66 \| \|  \|  \| 4,7 \| 82,9 \| 11,6 \| 5,5 \| 42,903 \| 43,5645 \| 5,9535 \| 1,7955 \| 0,2835 \| 900 \| 1,8 \| 100,13 \| \|  \|  \| 4,7 \| 82,9 \| 11,6 \| 5,5 \| 42,903 \| 43,5645 \| 5,9535 \| 1,7955 \| 0,2835 \| 900 \| 2 \| 89,05 \| \|  \|  \| 4,7 \| 82,9 \| 11,6 \| 5,5 \| 42,903 \| 43,5645 \| 5,9535 \| 1,7955 \| 0,2835 \| 900 \| 2,3 \| 77,33 \| \|  \|  \| 4,7 \| 82,9 \| 11,6 \| 5,5 \| 42,903 \| 43,5645 \| 5,9535 \| 1,7955 \| 0,2835 \| 1200 \| 1,8 \| 136,02 \| \|  \|  \| 4,7 \| 82,9 \| 11,6 \| 5,5 \| 42,903 \| 43,5645 \| 5,9535 \| 1,7955 \| 0,2835 \| 1200 \| 2 \| 128,18 \| \|  \|  \| 4,7 \| 82,9 \| 11,6 \| 5,5 \| 42,903 \| 43,5645 \| 5,9535 \| 1,7955 \| 0,2835 \| 1200 \| 2,3 \| 119,58 \| \|  \|  \| 4,7 \| 82,9 \| 11,6 \| 5,5 \| 42,903 \| 43,5645 \| 5,9535 \| 1,7955 \| 0,2835 \| 1500 \| 1,8 \| 170,93 \| \|  \|  \| 4,7 \| 82,9 \| 11,6 \| 5,5 \| 42,903 \| 43,5645 \| 5,9535 \| 1,7955 \| 0,2835 \| 1500 \| 2 \| 166,39 \| \|  \|  \| 4,7 \| 82,9 \| 11,6 \| 5,5 \| 42,903 \| 43,5645 \| 5,9535 \| 1,7955 \| 0,2835 \| 1500 \| 2,3 \| 161,00 \| \| 80 \| Demolition wood \| 16,3 \| 75,8 \| 17,3 \| 6,9 \| 48,1327 \| 37,8917 \| 5,9584 \| 1,0241 \| 0,0931 \| 600 \| 1,8 \| 71,88 \| \|  \|  \| 16,3 \| 75,8 \| 17,3 \| 6,9 \| 48,1327 \| 37,8917 \| 5,9584 \| 1,0241 \| 0,0931 \| 600 \| 2 \| 65,85 \| \|  \|  \| 16,3 \| 75,8 \| 17,3 \| 6,9 \| 48,1327 \| 37,8917 \| 5,9584 \| 1,0241 \| 0,0931 \| 600 \| 2,3 \| 59,37 \| \|  \|  \| 16,3 \| 75,8 \| 17,3 \| 6,9 \| 48,1327 \| 37,8917 \| 5,9584 \| 1,0241 \| 0,0931 \| 900 \| 1,8 \| 158,99 \| \|  \|  \| 16,3 \| 75,8 \| 17,3 \| 6,9 \| 48,1327 \| 37,8917 \| 5,9584 \| 1,0241 \| 0,0931 \| 900 \| 2 \| 148,66 \| \|  \|  \| 16,3 \| 75,8 \| 17,3 \| 6,9 \| 48,1327 \| 37,8917 \| 5,9584 \| 1,0241 \| 0,0931 \| 900 \| 2,3 \| 137,03 \| \|  \|  \| 16,3 \| 75,8 \| 17,3 \| 6,9 \| 48,1327 \| 37,8917 \| 5,9584 \| 1,0241 \| 0,0931 \| 1200 \| 1,8 \| 191,53 \| \|  \|  \| 16,3 \| 75,8 \| 17,3 \| 6,9 \| 48,1327 \| 37,8917 \| 5,9584 \| 1,0241 \| 0,0931 \| 1200 \| 2 \| 185,16 \| \|  \|  \| 16,3 \| 75,8 \| 17,3 \| 6,9 \| 48,1327 \| 37,8917 \| 5,9584 \| 1,0241 \| 0,0931 \| 1200 \| 2,3 \| 177,31 \| \|  \|  \| 16,3 \| 75,8 \| 17,3 \| 6,9 \| 48,1327 \| 37,8917 \| 5,9584 \| 1,0241 \| 0,0931 \| 1500 \| 1,8 \| 222,82 \| \|  \|  \| 16,3 \| 75,8 \| 17,3 \| 6,9 \| 48,1327 \| 37,8917 \| 5,9584 \| 1,0241 \| 0,0931 \| 1500 \| 2 \| 220,38 \| \|  \|  \| 16,3 \| 75,8 \| 17,3 \| 6,9 \| 48,1327 \| 37,8917 \| 5,9584 \| 1,0241 \| 0,0931 \| 1500 \| 2,3 \| 216,39 \| \| 81 \| Furniture waste \| 12,1 \| 83 \| 13,4 \| 3,6 \| 49,9352 \| 40,2952 \| 5,8804 \| 0,2892 \| 0 \| 600 \| 1,8 \| 81,15 \| \|  \|  \| 12,1 \| 83 \| 13,4 \| 3,6 \| 49,9352 \| 40,2952 \| 5,8804 \| 0,2892 \| 0 \| 600 \| 2 \| 74,98 \| \|  \|  \| 12,1 \| 83 \| 13,4 \| 3,6 \| 49,9352 \| 40,2952 \| 5,8804 \| 0,2892 \| 0 \| 600 \| 2,3 \| 68,31 \| \|  \|  \| 12,1 \| 83 \| 13,4 \| 3,6 \| 49,9352 \| 40,2952 \| 5,8804 \| 0,2892 \| 0 \| 900 \| 1,8 \| 174,69 \| \|  \|  \| 12,1 \| 83 \| 13,4 \| 3,6 \| 49,9352 \| 40,2952 \| 5,8804 \| 0,2892 \| 0 \| 900 \| 2 \| 164,25 \| \|  \|  \| 12,1 \| 83 \| 13,4 \| 3,6 \| 49,9352 \| 40,2952 \| 5,8804 \| 0,2892 \| 0 \| 900 \| 2,3 \| 152,35 \| \|  \|  \| 12,1 \| 83 \| 13,4 \| 3,6 \| 49,9352 \| 40,2952 \| 5,8804 \| 0,2892 \| 0 \| 1200 \| 1,8 \| 208,62 \| \|  \|  \| 12,1 \| 83 \| 13,4 \| 3,6 \| 49,9352 \| 40,2952 \| 5,8804 \| 0,2892 \| 0 \| 1200 \| 2 \| 202,42 \| \|  \|  \| 12,1 \| 83 \| 13,4 \| 3,6 \| 49,9352 \| 40,2952 \| 5,8804 \| 0,2892 \| 0 \| 1200 \| 2,3 \| 194,56 \| \|  \|  \| 12,1 \| 83 \| 13,4 \| 3,6 \| 49,9352 \| 40,2952 \| 5,8804 \| 0,2892 \| 0 \| 1500 \| 1,8 \| 241,19 \| \|  \|  \| 12,1 \| 83 \| 13,4 \| 3,6 \| 49,9352 \| 40,2952 \| 5,8804 \| 0,2892 \| 0 \| 1500 \| 2 \| 239,13 \| \|  \|  \| 12,1 \| 83 \| 13,4 \| 3,6 \| 49,9352 \| 40,2952 \| 5,8804 \| 0,2892 \| 0 \| 1500 \| 2,3 \| 235,39 \| \| 82 \| Mixed waste paper \| 8,8 \| 84,2 \| 7,5 \| 8,3 \| 47,9591 \| 36,8634 \| 6,6024 \| 0,1834 \| 0,0917 \| 600 \| 1,8 \| 101,62 \| \|  \|  \| 8,8 \| 84,2 \| 7,5 \| 8,3 \| 47,9591 \| 36,8634 \| 6,6024 \| 0,1834 \| 0,0917 \| 600 \| 2 \| 95,51 \| \|  \|  \| 8,8 \| 84,2 \| 7,5 \| 8,3 \| 47,9591 \| 36,8634 \| 6,6024 \| 0,1834 \| 0,0917 \| 600 \| 2,3 \| 88,81 \| \|  \|  \| 8,8 \| 84,2 \| 7,5 \| 8,3 \| 47,9591 \| 36,8634 \| 6,6024 \| 0,1834 \| 0,0917 \| 900 \| 1,8 \| 197,67 \| \|  \|  \| 8,8 \| 84,2 \| 7,5 \| 8,3 \| 47,9591 \| 36,8634 \| 6,6024 \| 0,1834 \| 0,0917 \| 900 \| 2 \| 187,68 \| \|  \|  \| 8,8 \| 84,2 \| 7,5 \| 8,3 \| 47,9591 \| 36,8634 \| 6,6024 \| 0,1834 \| 0,0917 \| 900 \| 2,3 \| 175,99 \| \|  \|  \| 8,8 \| 84,2 \| 7,5 \| 8,3 \| 47,9591 \| 36,8634 \| 6,6024 \| 0,1834 \| 0,0917 \| 1200 \| 1,8 \| 232,42 \| \|  \|  \| 8,8 \| 84,2 \| 7,5 \| 8,3 \| 47,9591 \| 36,8634 \| 6,6024 \| 0,1834 \| 0,0917 \| 1200 \| 2 \| 226,90 \| \|  \|  \| 8,8 \| 84,2 \| 7,5 \| 8,3 \| 47,9591 \| 36,8634 \| 6,6024 \| 0,1834 \| 0,0917 \| 1200 \| 2,3 \| 219,52 \| \|  \|  \| 8,8 \| 84,2 \| 7,5 \| 8,3 \| 47,9591 \| 36,8634 \| 6,6024 \| 0,1834 \| 0,0917 \| 1500 \| 1,8 \| 265,76 \| \|  \|  \| 8,8 \| 84,2 \| 7,5 \| 8,3 \| 47,9591 \| 36,8634 \| 6,6024 \| 0,1834 \| 0,0917 \| 1500 \| 2 \| 264,53 \| \|  \|  \| 8,8 \| 84,2 \| 7,5 \| 8,3 \| 47,9591 \| 36,8634 \| 6,6024 \| 0,1834 \| 0,0917 \| 1500 \| 2,3 \| 261,48 \| \| 83 \| Greenhouse-plastic waste \| 2,5 \| 62,6 \| 5,6 \| 31,8 \| 48,3538 \| 11,1848 \| 7,6384 \| 1,023 \| 0 \| 600 \| 1,8 \| 195,48 \| \|  \|  \| 2,5 \| 62,6 \| 5,6 \| 31,8 \| 48,3538 \| 11,1848 \| 7,6384 \| 1,023 \| 0 \| 600 \| 2 \| 194,33 \| \|  \|  \| 2,5 \| 62,6 \| 5,6 \| 31,8 \| 48,3538 \| 11,1848 \| 7,6384 \| 1,023 \| 0 \| 600 \| 2,3 \| 190,77 \| \|  \|  \| 2,5 \| 62,6 \| 5,6 \| 31,8 \| 48,3538 \| 11,1848 \| 7,6384 \| 1,023 \| 0 \| 900 \| 1,8 \| 295,67 \| \|  \|  \| 2,5 \| 62,6 \| 5,6 \| 31,8 \| 48,3538 \| 11,1848 \| 7,6384 \| 1,023 \| 0 \| 900 \| 2 \| 306,85 \| \|  \|  \| 2,5 \| 62,6 \| 5,6 \| 31,8 \| 48,3538 \| 11,1848 \| 7,6384 \| 1,023 \| 0 \| 900 \| 2,3 \| 304,28 \| \|  \|  \| 2,5 \| 62,6 \| 5,6 \| 31,8 \| 48,3538 \| 11,1848 \| 7,6384 \| 1,023 \| 0 \| 1200 \| 1,8 \| 327,52 \| \|  \|  \| 2,5 \| 62,6 \| 5,6 \| 31,8 \| 48,3538 \| 11,1848 \| 7,6384 \| 1,023 \| 0 \| 1200 \| 2 \| 339,73 \| \|  \|  \| 2,5 \| 62,6 \| 5,6 \| 31,8 \| 48,3538 \| 11,1848 \| 7,6384 \| 1,023 \| 0 \| 1200 \| 2,3 \| 340,55 \| \|  \|  \| 2,5 \| 62,6 \| 5,6 \| 31,8 \| 48,3538 \| 11,1848 \| 7,6384 \| 1,023 \| 0 \| 1500 \| 1,8 \| 356,97 \| \|  \|  \| 2,5 \| 62,6 \| 5,6 \| 31,8 \| 48,3538 \| 11,1848 \| 7,6384 \| 1,023 \| 0 \| 1500 \| 2 \| 371,24 \| \|  \|  \| 2,5 \| 62,6 \| 5,6 \| 31,8 \| 48,3538 \| 11,1848 \| 7,6384 \| 1,023 \| 0 \| 1500 \| 2,3 \| 375,79 \| \| 84 \| Refuse-derived fuel \| 4,2 \| 73,4 \| 0,5 \| 26,1 \| 39,7582 \| 27,1952 \| 5,7642 \| 0,8129 \| 0,3695 \| 600 \| 1,8 \| 17,89 \| \|  \|  \| 4,2 \| 73,4 \| 0,5 \| 26,1 \| 39,7582 \| 27,1952 \| 5,7642 \| 0,8129 \| 0,3695 \| 600 \| 2 \| 9,85 \| \|  \|  \| 4,2 \| 73,4 \| 0,5 \| 26,1 \| 39,7582 \| 27,1952 \| 5,7642 \| 0,8129 \| 0,3695 \| 600 \| 2,3 \| 1,42 \| \|  \|  \| 4,2 \| 73,4 \| 0,5 \| 26,1 \| 39,7582 \| 27,1952 \| 5,7642 \| 0,8129 \| 0,3695 \| 900 \| 1,8 \| 101,56 \| \|  \|  \| 4,2 \| 73,4 \| 0,5 \| 26,1 \| 39,7582 \| 27,1952 \| 5,7642 \| 0,8129 \| 0,3695 \| 900 \| 2 \| 87,09 \| \|  \|  \| 4,2 \| 73,4 \| 0,5 \| 26,1 \| 39,7582 \| 27,1952 \| 5,7642 \| 0,8129 \| 0,3695 \| 900 \| 2,3 \| 71,80 \| \|  \|  \| 4,2 \| 73,4 \| 0,5 \| 26,1 \| 39,7582 \| 27,1952 \| 5,7642 \| 0,8129 \| 0,3695 \| 1200 \| 1,8 \| 139,35 \| \|  \|  \| 4,2 \| 73,4 \| 0,5 \| 26,1 \| 39,7582 \| 27,1952 \| 5,7642 \| 0,8129 \| 0,3695 \| 1200 \| 2 \| 128,83 \| \|  \|  \| 4,2 \| 73,4 \| 0,5 \| 26,1 \| 39,7582 \| 27,1952 \| 5,7642 \| 0,8129 \| 0,3695 \| 1200 \| 2,3 \| 117,34 \| \|  \|  \| 4,2 \| 73,4 \| 0,5 \| 26,1 \| 39,7582 \| 27,1952 \| 5,7642 \| 0,8129 \| 0,3695 \| 1500 \| 1,8 \| 176,35 \| \|  \|  \| 4,2 \| 73,4 \| 0,5 \| 26,1 \| 39,7582 \| 27,1952 \| 5,7642 \| 0,8129 \| 0,3695 \| 1500 \| 2 \| 169,85 \| \|  \|  \| 4,2 \| 73,4 \| 0,5 \| 26,1 \| 39,7582 \| 27,1952 \| 5,7642 \| 0,8129 \| 0,3695 \| 1500 \| 2,3 \| 162,27 \| \| 85 \| Sewage sludge \| 6,4 \| 48 \| 5,7 \| 46,3 \| 27,3333 \| 17,9358 \| 3,9201 \| 3,2757 \| 1,2351 \| 600 \| 1,8 \| 0,00 \| \|  \|  \| 6,4 \| 48 \| 5,7 \| 46,3 \| 27,3333 \| 17,9358 \| 3,9201 \| 3,2757 \| 1,2351 \| 600 \| 2 \| 0,00 \| \|  \|  \| 6,4 \| 48 \| 5,7 \| 46,3 \| 27,3333 \| 17,9358 \| 3,9201 \| 3,2757 \| 1,2351 \| 600 \| 2,3 \| 0,00 \| \|  \|  \| 6,4 \| 48 \| 5,7 \| 46,3 \| 27,3333 \| 17,9358 \| 3,9201 \| 3,2757 \| 1,2351 \| 900 \| 1,8 \| 0,00 \| \|  \|  \| 6,4 \| 48 \| 5,7 \| 46,3 \| 27,3333 \| 17,9358 \| 3,9201 \| 3,2757 \| 1,2351 \| 900 \| 2 \| 0,00 \| \|  \|  \| 6,4 \| 48 \| 5,7 \| 46,3 \| 27,3333 \| 17,9358 \| 3,9201 \| 3,2757 \| 1,2351 \| 900 \| 2,3 \| 0,00 \| \|  \|  \| 6,4 \| 48 \| 5,7 \| 46,3 \| 27,3333 \| 17,9358 \| 3,9201 \| 3,2757 \| 1,2351 \| 1200 \| 1,8 \| 0,00 \| \|  \|  \| 6,4 \| 48 \| 5,7 \| 46,3 \| 27,3333 \| 17,9358 \| 3,9201 \| 3,2757 \| 1,2351 \| 1200 \| 2 \| 0,00 \| \|  \|  \| 6,4 \| 48 \| 5,7 \| 46,3 \| 27,3333 \| 17,9358 \| 3,9201 \| 3,2757 \| 1,2351 \| 1200 \| 2,3 \| 0,00 \| \|  \|  \| 6,4 \| 48 \| 5,7 \| 46,3 \| 27,3333 \| 17,9358 \| 3,9201 \| 3,2757 \| 1,2351 \| 1500 \| 1,8 \| 0,00 \| \|  \|  \| 6,4 \| 48 \| 5,7 \| 46,3 \| 27,3333 \| 17,9358 \| 3,9201 \| 3,2757 \| 1,2351 \| 1500 \| 2 \| 0,00 \| \|  \|  \| 6,4 \| 48 \| 5,7 \| 46,3 \| 27,3333 \| 17,9358 \| 3,9201 \| 3,2757 \| 1,2351 \| 1500 \| 2,3 \| 0,00 \| \| 86 \| Wood yard waste \| 38,1 \| 66 \| 13,6 \| 20,4 \| 41,5512 \| 32,1584 \| 4,776 \| 0,8756 \| 0,2388 \| 600 \| 1,8 \| 0,00 \| \|  \|  \| 38,1 \| 66 \| 13,6 \| 20,4 \| 41,5512 \| 32,1584 \| 4,776 \| 0,8756 \| 0,2388 \| 600 \| 2 \| 0,00 \| \|  \|  \| 38,1 \| 66 \| 13,6 \| 20,4 \| 41,5512 \| 32,1584 \| 4,776 \| 0,8756 \| 0,2388 \| 600 \| 2,3 \| 0,00 \| \|  \|  \| 38,1 \| 66 \| 13,6 \| 20,4 \| 41,5512 \| 32,1584 \| 4,776 \| 0,8756 \| 0,2388 \| 900 \| 1,8 \| 47,89 \| \|  \|  \| 38,1 \| 66 \| 13,6 \| 20,4 \| 41,5512 \| 32,1584 \| 4,776 \| 0,8756 \| 0,2388 \| 900 \| 2 \| 38,07 \| \|  \|  \| 38,1 \| 66 \| 13,6 \| 20,4 \| 41,5512 \| 32,1584 \| 4,776 \| 0,8756 \| 0,2388 \| 900 \| 2,3 \| 27,78 \| \|  \|  \| 38,1 \| 66 \| 13,6 \| 20,4 \| 41,5512 \| 32,1584 \| 4,776 \| 0,8756 \| 0,2388 \| 1200 \| 1,8 \| 72,57 \| \|  \|  \| 38,1 \| 66 \| 13,6 \| 20,4 \| 41,5512 \| 32,1584 \| 4,776 \| 0,8756 \| 0,2388 \| 1200 \| 2 \| 65,23 \| \|  \|  \| 38,1 \| 66 \| 13,6 \| 20,4 \| 41,5512 \| 32,1584 \| 4,776 \| 0,8756 \| 0,2388 \| 1200 \| 2,3 \| 57,35 \| \|  \|  \| 38,1 \| 66 \| 13,6 \| 20,4 \| 41,5512 \| 32,1584 \| 4,776 \| 0,8756 \| 0,2388 \| 1500 \| 1,8 \| 96,83 \| \|  \|  \| 38,1 \| 66 \| 13,6 \| 20,4 \| 41,5512 \| 32,1584 \| 4,776 \| 0,8756 \| 0,2388 \| 1500 \| 2 \| 92,01 \| \|  \|  \| 38,1 \| 66 \| 13,6 \| 20,4 \| 41,5512 \| 32,1584 \| 4,776 \| 0,8756 \| 0,2388 \| 1500 \| 2,3 \| 86,59 \| |
| --- | --- | --- | --- | --- | --- | --- | --- | --- | --- | --- | --- | --- | --- | --- | --- | --- | --- | --- | --- | --- | --- | --- | --- | --- | --- | --- | --- | --- | --- | --- | --- | --- | --- | --- | --- | --- | --- | --- | --- | --- | --- | --- | --- | --- | --- | --- | --- | --- | --- | --- | --- | --- | --- | --- | --- | --- | --- | --- | --- | --- | --- | --- | --- | --- | --- | --- | --- | --- | --- | --- | --- | --- | --- | --- | --- | --- | --- | --- | --- | --- | --- | --- | --- | --- | --- | --- | --- | --- | --- | --- | --- | --- | --- | --- | --- | --- | --- | --- | --- | --- | --- | --- | --- | --- | --- | --- | --- | --- | --- | --- | --- | --- | --- | --- | --- | --- | --- | --- | --- | --- | --- | --- | --- | --- | --- | --- | --- | --- | --- | --- | --- | --- | --- | --- | --- | --- | --- | --- | --- | --- | --- | --- | --- | --- | --- | --- | --- | --- | --- | --- | --- | --- | --- | --- | --- | --- | --- | --- | --- | --- | --- | --- | --- | --- | --- | --- | --- | --- | --- | --- | --- | --- | --- | --- | --- | --- | --- | --- | --- | --- | --- | --- | --- | --- | --- | --- | --- | --- | --- | --- | --- | --- | --- | --- | --- | --- | --- | --- | --- | --- | --- | --- | --- | --- | --- | --- | --- | --- | --- | --- | --- | --- | --- | --- | --- | --- | --- | --- | --- | --- | --- | --- | --- | --- | --- | --- | --- | --- | --- | --- | --- | --- | --- | --- | --- | --- | --- | --- | --- | --- | --- | --- | --- | --- | --- | --- | --- | --- | --- | --- | --- | --- | --- | --- | --- | --- | --- | --- | --- | --- | --- | --- | --- | --- | --- | --- | --- | --- | --- | --- | --- | --- | --- | --- | --- | --- | --- | --- | --- | --- | --- | --- | --- | --- | --- | --- | --- | --- | --- | --- | --- | --- | --- | --- | --- | --- | --- | --- | --- | --- | --- | --- | --- | --- | --- | --- | --- | --- | --- | --- | --- | --- | --- | --- | --- | --- | --- | --- | --- | --- | --- | --- | --- | --- | --- | --- | --- | --- | --- | --- | --- | --- | --- | --- | --- | --- | --- | --- | --- | --- | --- | --- | --- | --- | --- | --- | --- | --- | --- | --- | --- | --- | --- | --- | --- | --- | --- | --- | --- | --- | --- | --- | --- | --- | --- | --- | --- | --- | --- | --- | --- | --- | --- | --- | --- | --- | --- | --- | --- | --- | --- | --- | --- | --- | --- | --- | --- | --- | --- | --- | --- | --- | --- | --- | --- | --- | --- | --- | --- | --- | --- | --- | --- | --- | --- | --- | --- | --- | --- | --- | --- | --- | --- | --- | --- | --- | --- | --- | --- | --- | --- | --- | --- | --- | --- | --- | --- | --- | --- | --- | --- | --- | --- | --- | --- | --- | --- | --- | --- | --- | --- | --- | --- | --- | --- | --- | --- | --- | --- | --- | --- | --- | --- | --- | --- | --- | --- | --- | --- | --- | --- | --- | --- | --- | --- | --- | --- | --- | --- | --- | --- | --- | --- | --- | --- | --- | --- | --- | --- | --- | --- | --- | --- | --- | --- | --- | --- | --- | --- | --- | --- | --- | --- | --- | --- | --- | --- | --- | --- | --- | --- | --- | --- | --- | --- | --- | --- | --- | --- | --- | --- | --- | --- | --- | --- | --- | --- | --- | --- | --- | --- | --- | --- | --- | --- | --- | --- | --- | --- | --- | --- | --- | --- | --- | --- | --- | --- | --- | --- | --- | --- | --- | --- | --- | --- | --- | --- | --- | --- | --- | --- | --- | --- | --- | --- | --- | --- | --- | --- | --- | --- | --- | --- | --- | --- | --- | --- | --- | --- | --- | --- | --- | --- | --- | --- | --- | --- | --- | --- | --- | --- | --- | --- | --- | --- | --- | --- | --- | --- | --- | --- | --- | --- | --- | --- | --- | --- | --- | --- | --- | --- | --- | --- | --- | --- | --- | --- | --- | --- | --- | --- | --- | --- | --- | --- | --- | --- | --- | --- | --- | --- | --- | --- | --- | --- | --- | --- | --- | --- | --- | --- | --- | --- | --- | --- | --- | --- | --- | --- | --- | --- | --- | --- | --- | --- | --- | --- | --- | --- | --- | --- | --- | --- | --- | --- | --- | --- | --- | --- | --- | --- | --- | --- | --- | --- | --- | --- | --- | --- | --- | --- | --- | --- | --- | --- | --- | --- | --- | --- | --- | --- | --- | --- | --- | --- | --- | --- | --- | --- | --- | --- | --- | --- | --- | --- | --- | --- | --- | --- | --- | --- | --- | --- | --- | --- | --- | --- | --- | --- | --- | --- | --- | --- | --- | --- | --- | --- | --- | --- | --- | --- | --- | --- | --- | --- | --- | --- | --- | --- | --- | --- | --- | --- | --- | --- | --- | --- | --- | --- | --- | --- | --- | --- | --- | --- | --- | --- | --- | --- | --- | --- | --- | --- | --- | --- | --- | --- | --- | --- | --- | --- | --- | --- | --- | --- | --- | --- | --- | --- | --- | --- | --- | --- | --- | --- | --- | --- | --- | --- | --- | --- | --- | --- | --- | --- | --- | --- | --- | --- | --- | --- | --- | --- | --- | --- | --- | --- | --- | --- | --- | --- | --- | --- | --- | --- | --- | --- | --- | --- | --- | --- | --- | --- | --- | --- | --- | --- | --- | --- | --- | --- | --- | --- | --- | --- | --- | --- | --- | --- | --- | --- | --- | --- | --- | --- | --- | --- | --- | --- | --- | --- | --- | --- | --- | --- | --- | --- | --- | --- | --- | --- | --- | --- | --- | --- | --- | --- | --- | --- | --- | --- | --- | --- | --- | --- | --- | --- | --- | --- | --- | --- | --- | --- | --- | --- | --- | --- | --- | --- | --- | --- | --- | --- | --- | --- | --- | --- | --- | --- | --- | --- | --- | --- | --- | --- | --- | --- | --- | --- | --- | --- | --- | --- | --- | --- | --- | --- | --- | --- | --- | --- | --- | --- | --- | --- | --- | --- | --- | --- | --- | --- | --- | --- | --- | --- | --- | --- | --- | --- | --- | --- | --- | --- | --- | --- | --- | --- | --- | --- | --- | --- | --- | --- | --- | --- | --- | --- | --- | --- | --- | --- | --- | --- | --- | --- | --- | --- | --- | --- | --- | --- | --- | --- | --- | --- | --- | --- | --- | --- | --- | --- | --- | --- | --- | --- | --- | --- | --- | --- | --- | --- | --- | --- | --- | --- | --- | --- | --- | --- | --- | --- | --- | --- | --- | --- | --- | --- | --- | --- | --- | --- | --- | --- | --- | --- | --- | --- | --- | --- | --- | --- | --- | --- | --- | --- | --- | --- | --- | --- | --- | --- | --- | --- | --- | --- | --- | --- | --- | --- | --- | --- | --- | --- | --- | --- | --- | --- | --- | --- | --- | --- | --- | --- | --- | --- | --- | --- | --- | --- | --- | --- | --- | --- | --- | --- | --- | --- | --- | --- | --- | --- | --- | --- | --- | --- | --- | --- | --- | --- | --- | --- | --- | --- | --- | --- | --- | --- | --- | --- | --- | --- | --- | --- | --- | --- | --- | --- | --- | --- | --- | --- | --- | --- | --- | --- | --- | --- | --- | --- | --- | --- | --- | --- | --- | --- | --- | --- | --- | --- | --- | --- | --- | --- | --- | --- | --- | --- | --- | --- | --- | --- | --- | --- | --- | --- | --- | --- | --- | --- | --- | --- | --- | --- | --- | --- | --- | --- | --- | --- | --- | --- | --- | --- | --- | --- | --- | --- | --- | --- | --- | --- | --- | --- | --- | --- | --- | --- | --- | --- | --- | --- | --- | --- | --- | --- | --- | --- | --- | --- | --- | --- | --- | --- | --- | --- | --- | --- | --- | --- | --- | --- | --- | --- | --- | --- | --- | --- | --- | --- | --- | --- | --- | --- | --- | --- | --- | --- | --- | --- | --- | --- | --- | --- | --- | --- | --- | --- | --- | --- | --- | --- | --- | --- | --- | --- | --- | --- | --- | --- | --- | --- | --- | --- | --- | --- | --- | --- | --- | --- | --- | --- | --- | --- | --- | --- | --- | --- | --- | --- | --- | --- | --- | --- | --- | --- | --- | --- | --- | --- | --- | --- | --- | --- | --- | --- | --- | --- | --- | --- | --- | --- | --- | --- | --- | --- | --- | --- | --- | --- | --- | --- | --- | --- | --- | --- | --- | --- | --- | --- | --- | --- | --- | --- | --- | --- | --- | --- | --- | --- | --- | --- | --- | --- | --- | --- | --- | --- | --- | --- | --- | --- | --- | --- | --- | --- | --- | --- | --- | --- | --- | --- | --- | --- | --- | --- | --- | --- | --- | --- | --- | --- | --- | --- | --- | --- | --- | --- | --- | --- | --- | --- | --- | --- | --- | --- | --- | --- | --- | --- | --- | --- | --- | --- | --- | --- | --- | --- | --- | --- | --- | --- | --- | --- | --- | --- | --- | --- | --- | --- | --- | --- | --- | --- | --- | --- | --- | --- | --- | --- | --- | --- | --- | --- | --- | --- | --- | --- | --- | --- | --- | --- | --- | --- | --- | --- | --- | --- | --- | --- | --- | --- | --- | --- | --- | --- | --- | --- | --- | --- | --- | --- | --- | --- | --- | --- | --- | --- | --- | --- | --- | --- | --- | --- | --- | --- | --- | --- | --- | --- | --- | --- | --- | --- | --- | --- | --- | --- | --- | --- | --- | --- | --- | --- | --- | --- | --- | --- | --- | --- | --- | --- | --- | --- | --- | --- | --- | --- | --- | --- | --- | --- | --- | --- | --- | --- | --- | --- | --- | --- | --- | --- | --- | --- | --- | --- | --- | --- | --- | --- | --- | --- | --- | --- | --- | --- | --- | --- | --- | --- | --- | --- | --- | --- | --- | --- | --- | --- | --- | --- | --- | --- | --- | --- | --- | --- | --- | --- | --- | --- | --- | --- | --- | --- | --- | --- | --- | --- | --- | --- | --- | --- | --- | --- | --- | --- | --- | --- | --- | --- | --- | --- | --- | --- | --- | --- | --- | --- | --- | --- | --- | --- | --- | --- | --- | --- | --- | --- | --- | --- | --- | --- | --- | --- | --- | --- | --- | --- | --- | --- | --- | --- | --- | --- | --- | --- | --- | --- | --- | --- | --- | --- | --- | --- | --- | --- | --- | --- | --- | --- | --- | --- | --- | --- | --- | --- | --- | --- | --- | --- | --- | --- | --- | --- | --- | --- | --- | --- | --- | --- | --- | --- | --- | --- | --- | --- | --- | --- | --- | --- | --- | --- | --- | --- | --- | --- | --- | --- | --- | --- | --- | --- | --- | --- | --- | --- | --- | --- | --- | --- | --- | --- | --- | --- | --- | --- | --- | --- | --- | --- | --- | --- | --- | --- | --- | --- | --- | --- | --- | --- | --- | --- | --- | --- | --- | --- | --- | --- | --- | --- | --- | --- | --- | --- | --- | --- | --- | --- | --- | --- | --- | --- | --- | --- | --- | --- | --- | --- | --- | --- | --- | --- | --- | --- | --- | --- | --- | --- | --- | --- | --- | --- | --- | --- | --- | --- | --- | --- | --- | --- | --- | --- | --- | --- | --- | --- | --- | --- | --- | --- | --- | --- | --- | --- | --- | --- | --- | --- | --- | --- | --- | --- | --- | --- | --- | --- | --- | --- | --- | --- | --- | --- | --- | --- | --- | --- | --- | --- | --- | --- | --- | --- | --- | --- | --- | --- | --- | --- | --- | --- | --- | --- | --- | --- | --- | --- | --- | --- | --- | --- | --- | --- | --- | --- | --- | --- | --- | --- | --- | --- | --- | --- | --- | --- | --- | --- | --- | --- | --- | --- | --- | --- | --- | --- | --- | --- | --- | --- | --- | --- | --- | --- | --- | --- | --- | --- | --- | --- | --- | --- | --- | --- | --- | --- | --- | --- | --- | --- | --- | --- | --- | --- | --- | --- | --- | --- | --- | --- | --- | --- | --- | --- | --- | --- | --- | --- | --- | --- | --- | --- | --- | --- | --- | --- | --- | --- | --- | --- | --- | --- | --- | --- | --- | --- | --- | --- | --- | --- | --- | --- | --- | --- | --- | --- | --- | --- | --- | --- | --- | --- | --- | --- | --- | --- | --- | --- | --- | --- | --- | --- | --- | --- | --- | --- | --- | --- | --- | --- | --- | --- | --- | --- | --- | --- | --- | --- | --- | --- | --- | --- | --- | --- | --- | --- | --- | --- | --- | --- | --- | --- | --- | --- | --- | --- | --- | --- | --- | --- | --- | --- | --- | --- | --- | --- | --- | --- | --- | --- | --- | --- | --- | --- | --- | --- | --- | --- | --- | --- | --- | --- | --- | --- | --- | --- | --- | --- | --- | --- | --- | --- | --- | --- | --- | --- | --- | --- | --- | --- | --- | --- | --- | --- | --- | --- | --- | --- | --- | --- | --- | --- | --- | --- | --- | --- | --- | --- | --- | --- | --- | --- | --- | --- | --- | --- | --- | --- | --- | --- | --- | --- | --- | --- | --- | --- | --- | --- | --- | --- | --- | --- | --- | --- | --- | --- | --- | --- | --- | --- | --- | --- | --- | --- | --- | --- | --- | --- | --- | --- | --- | --- | --- | --- | --- | --- | --- | --- | --- | --- | --- | --- | --- | --- | --- | --- | --- | --- | --- | --- | --- | --- | --- | --- | --- | --- | --- | --- | --- | --- | --- | --- | --- | --- | --- | --- | --- | --- | --- | --- | --- | --- | --- | --- | --- | --- | --- | --- | --- | --- | --- | --- | --- | --- | --- | --- | --- | --- | --- | --- | --- | --- | --- | --- | --- | --- | --- | --- | --- | --- | --- | --- | --- | --- | --- | --- | --- | --- | --- | --- | --- | --- | --- | --- | --- | --- | --- | --- | --- | --- | --- | --- | --- | --- | --- | --- | --- | --- | --- | --- | --- | --- | --- | --- | --- | --- | --- | --- | --- | --- | --- | --- | --- | --- | --- | --- | --- | --- | --- | --- | --- | --- | --- | --- | --- | --- | --- | --- | --- | --- | --- | --- | --- | --- | --- | --- | --- | --- | --- | --- | --- | --- | --- | --- | --- | --- | --- | --- | --- | --- | --- | --- | --- | --- | --- | --- | --- | --- | --- | --- | --- | --- | --- | --- | --- | --- | --- | --- | --- | --- | --- | --- | --- | --- | --- | --- | --- | --- | --- | --- | --- | --- | --- | --- | --- | --- | --- | --- | --- | --- | --- | --- | --- | --- | --- | --- | --- | --- | --- | --- | --- | --- | --- | --- | --- | --- | --- | --- | --- | --- | --- | --- | --- | --- | --- | --- | --- | --- | --- | --- | --- | --- | --- | --- | --- | --- | --- | --- | --- | --- | --- | --- | --- | --- | --- | --- | --- | --- | --- | --- | --- | --- | --- | --- | --- | --- | --- | --- | --- | --- | --- | --- | --- | --- | --- | --- | --- | --- | --- | --- | --- | --- | --- | --- | --- | --- | --- | --- | --- | --- | --- | --- | --- | --- | --- | --- | --- | --- | --- | --- | --- | --- | --- | --- | --- | --- | --- | --- | --- | --- | --- | --- | --- | --- | --- | --- | --- | --- | --- | --- | --- | --- | --- | --- | --- | --- | --- | --- | --- | --- | --- | --- | --- | --- | --- | --- | --- | --- | --- | --- | --- | --- | --- | --- | --- | --- | --- | --- | --- | --- | --- | --- | --- | --- | --- | --- | --- | --- | --- | --- | --- | --- | --- | --- | --- | --- | --- | --- | --- | --- | --- | --- | --- | --- | --- | --- | --- | --- | --- | --- | --- | --- | --- | --- | --- | --- | --- | --- | --- | --- | --- | --- | --- | --- | --- | --- | --- | --- | --- | --- | --- | --- | --- | --- | --- | --- | --- | --- | --- | --- | --- | --- | --- | --- | --- | --- | --- | --- | --- | --- | --- | --- | --- | --- | --- | --- | --- | --- | --- | --- | --- | --- | --- | --- | --- | --- | --- | --- | --- | --- | --- | --- | --- | --- | --- | --- | --- | --- | --- | --- | --- | --- | --- | --- | --- | --- | --- | --- | --- | --- | --- | --- | --- | --- | --- | --- | --- | --- | --- | --- | --- | --- | --- | --- | --- | --- | --- | --- | --- | --- | --- | --- | --- | --- | --- | --- | --- | --- | --- | --- | --- | --- | --- | --- | --- | --- | --- | --- | --- | --- | --- | --- | --- | --- | --- | --- | --- | --- | --- | --- | --- | --- | --- | --- | --- | --- | --- | --- | --- | --- | --- | --- | --- | --- | --- | --- | --- | --- | --- | --- | --- | --- | --- | --- | --- | --- | --- | --- | --- | --- | --- | --- | --- | --- | --- | --- | --- | --- | --- | --- | --- | --- | --- | --- | --- | --- | --- | --- | --- | --- | --- | --- | --- | --- | --- | --- | --- | --- | --- | --- | --- | --- | --- | --- | --- | --- | --- | --- | --- | --- | --- | --- | --- | --- | --- | --- | --- | --- | --- | --- | --- | --- | --- | --- | --- | --- | --- | --- | --- | --- | --- | --- | --- | --- | --- | --- | --- | --- | --- | --- | --- | --- | --- | --- | --- | --- | --- | --- | --- | --- | --- | --- | --- | --- | --- | --- | --- | --- | --- | --- | --- | --- | --- | --- | --- | --- | --- | --- | --- | --- | --- | --- | --- | --- | --- | --- | --- | --- | --- | --- | --- | --- | --- | --- | --- | --- | --- | --- | --- | --- | --- | --- | --- | --- | --- | --- | --- | --- | --- | --- | --- | --- | --- | --- | --- | --- | --- | --- | --- | --- | --- | --- | --- | --- | --- | --- | --- | --- | --- | --- | --- | --- | --- | --- | --- | --- | --- | --- | --- | --- | --- | --- | --- | --- | --- | --- | --- | --- | --- | --- | --- | --- | --- | --- | --- | --- | --- | --- | --- | --- | --- | --- | --- | --- | --- | --- | --- | --- | --- | --- | --- | --- | --- | --- | --- | --- | --- | --- | --- | --- | --- | --- | --- | --- | --- | --- | --- | --- | --- | --- | --- | --- | --- | --- | --- | --- | --- | --- | --- | --- | --- | --- | --- | --- | --- | --- | --- | --- | --- | --- | --- | --- | --- | --- | --- | --- | --- | --- | --- | --- | --- | --- | --- | --- | --- | --- | --- | --- | --- | --- | --- | --- | --- | --- | --- | --- | --- | --- | --- | --- | --- | --- | --- | --- | --- | --- | --- | --- | --- | --- | --- | --- | --- | --- | --- | --- | --- | --- | --- | --- | --- | --- | --- | --- | --- | --- | --- | --- | --- | --- | --- | --- | --- | --- | --- | --- | --- | --- | --- | --- | --- | --- | --- | --- | --- | --- | --- | --- | --- | --- | --- | --- | --- | --- | --- | --- | --- | --- | --- | --- | --- | --- | --- | --- | --- | --- | --- | --- | --- | --- | --- | --- | --- | --- | --- | --- | --- | --- | --- | --- | --- | --- | --- | --- | --- | --- | --- | --- | --- | --- | --- | --- | --- | --- | --- | --- | --- | --- | --- | --- | --- | --- | --- | --- | --- | --- | --- | --- | --- | --- | --- | --- | --- | --- | --- | --- | --- | --- | --- | --- | --- | --- | --- | --- | --- | --- | --- | --- | --- | --- | --- | --- | --- | --- | --- | --- | --- | --- | --- | --- | --- | --- | --- | --- | --- | --- | --- | --- | --- | --- | --- | --- | --- | --- | --- | --- | --- | --- | --- | --- | --- | --- | --- | --- | --- | --- | --- | --- | --- | --- | --- | --- | --- | --- | --- | --- | --- | --- | --- | --- | --- | --- | --- | --- | --- | --- | --- | --- | --- | --- | --- | --- | --- | --- | --- | --- | --- | --- | --- | --- | --- | --- | --- | --- | --- | --- | --- | --- | --- | --- | --- | --- | --- | --- | --- | --- | --- | --- | --- | --- | --- | --- | --- | --- | --- | --- | --- | --- | --- | --- | --- | --- | --- | --- | --- | --- | --- | --- | --- | --- | --- | --- | --- | --- | --- | --- | --- | --- | --- | --- | --- | --- | --- | --- | --- | --- | --- | --- | --- | --- | --- | --- | --- | --- | --- | --- | --- | --- | --- | --- | --- | --- | --- | --- | --- | --- | --- | --- | --- | --- | --- | --- | --- | --- | --- | --- | --- | --- | --- | --- | --- | --- | --- | --- | --- | --- | --- | --- | --- | --- | --- | --- | --- | --- | --- | --- | --- | --- | --- | --- | --- | --- | --- | --- | --- | --- | --- | --- | --- | --- | --- | --- | --- | --- | --- | --- | --- | --- | --- | --- | --- | --- | --- | --- | --- | --- | --- | --- | --- | --- | --- | --- | --- | --- | --- | --- | --- | --- | --- | --- | --- | --- | --- | --- | --- | --- | --- | --- | --- | --- | --- | --- | --- | --- | --- | --- | --- | --- | --- | --- | --- | --- | --- | --- | --- | --- | --- | --- | --- | --- | --- | --- | --- | --- | --- | --- | --- | --- | --- | --- | --- | --- | --- | --- | --- | --- | --- | --- | --- | --- | --- | --- | --- | --- | --- | --- | --- | --- | --- | --- | --- | --- | --- | --- | --- | --- | --- | --- | --- | --- | --- | --- | --- | --- | --- | --- | --- | --- | --- | --- | --- | --- | --- | --- | --- | --- | --- | --- | --- | --- | --- | --- | --- | --- | --- | --- | --- | --- | --- | --- | --- | --- | --- | --- | --- | --- | --- | --- | --- | --- | --- | --- | --- | --- | --- | --- | --- | --- | --- | --- | --- | --- | --- | --- | --- | --- | --- | --- | --- | --- | --- | --- | --- | --- | --- | --- | --- | --- | --- | --- | --- | --- | --- | --- | --- | --- | --- | --- | --- | --- | --- | --- | --- | --- | --- | --- | --- | --- | --- | --- | --- | --- | --- | --- | --- | --- | --- | --- | --- | --- | --- | --- | --- | --- | --- | --- | --- | --- | --- | --- | --- | --- | --- | --- | --- | --- | --- | --- | --- | --- | --- | --- | --- | --- | --- | --- | --- | --- | --- | --- | --- | --- | --- | --- | --- | --- | --- | --- | --- | --- | --- | --- | --- | --- | --- | --- | --- | --- | --- | --- | --- | --- | --- | --- | --- | --- | --- | --- | --- | --- | --- | --- | --- | --- | --- | --- | --- | --- | --- | --- | --- | --- | --- | --- | --- | --- | --- | --- | --- | --- | --- | --- | --- | --- | --- | --- | --- | --- | --- | --- | --- | --- | --- | --- | --- | --- | --- | --- | --- | --- | --- | --- | --- | --- | --- | --- | --- | --- | --- | --- | --- | --- | --- | --- | --- | --- | --- | --- | --- | --- | --- | --- | --- | --- | --- | --- | --- | --- | --- | --- | --- | --- | --- | --- | --- | --- | --- | --- | --- | --- | --- | --- | --- | --- | --- | --- | --- | --- | --- | --- | --- | --- | --- | --- | --- | --- | --- | --- | --- | --- | --- | --- | --- | --- | --- | --- | --- | --- | --- | --- | --- | --- | --- | --- | --- | --- | --- | --- | --- | --- | --- | --- | --- | --- | --- | --- | --- | --- | --- | --- | --- | --- | --- | --- | --- | --- | --- | --- | --- | --- | --- | --- | --- | --- | --- | --- | --- | --- | --- | --- | --- | --- | --- | --- | --- | --- | --- | --- | --- | --- | --- | --- | --- | --- | --- | --- | --- | --- | --- | --- | --- | --- | --- | --- | --- | --- | --- | --- | --- | --- | --- | --- | --- | --- | --- | --- | --- | --- | --- | --- | --- | --- | --- | --- | --- | --- | --- | --- | --- | --- | --- | --- | --- | --- | --- | --- | --- | --- | --- | --- | --- | --- | --- | --- | --- | --- | --- | --- | --- | --- | --- | --- | --- | --- | --- | --- | --- | --- | --- | --- | --- | --- | --- | --- | --- | --- | --- | --- | --- | --- | --- | --- | --- | --- | --- | --- | --- | --- | --- | --- | --- | --- | --- | --- | --- | --- | --- | --- | --- | --- | --- | --- | --- | --- | --- | --- | --- | --- | --- | --- | --- | --- | --- | --- | --- | --- | --- | --- | --- | --- | --- | --- | --- | --- | --- | --- | --- | --- | --- | --- | --- | --- | --- | --- | --- | --- | --- | --- | --- | --- | --- | --- | --- | --- | --- | --- | --- | --- | --- | --- | --- | --- | --- | --- | --- | --- | --- | --- | --- | --- | --- | --- | --- | --- | --- | --- | --- | --- | --- | --- | --- | --- | --- | --- | --- | --- | --- | --- | --- | --- | --- | --- | --- | --- | --- | --- | --- | --- | --- | --- | --- | --- | --- | --- | --- | --- | --- | --- | --- | --- | --- | --- | --- | --- | --- | --- | --- | --- | --- | --- | --- | --- | --- | --- | --- | --- | --- | --- | --- | --- | --- | --- | --- | --- | --- | --- | --- | --- | --- | --- | --- | --- | --- | --- | --- | --- | --- | --- | --- | --- | --- | --- | --- | --- | --- | --- | --- | --- | --- | --- | --- | --- | --- | --- | --- | --- | --- | --- | --- | --- | --- | --- | --- | --- | --- | --- | --- | --- | --- | --- | --- | --- | --- | --- | --- | --- | --- | --- | --- | --- | --- | --- | --- | --- | --- | --- | --- | --- | --- | --- | --- | --- | --- | --- | --- | --- | --- | --- | --- | --- | --- | --- | --- | --- | --- | --- | --- | --- | --- | --- | --- | --- | --- | --- | --- | --- | --- | --- | --- | --- | --- | --- | --- | --- | --- | --- | --- | --- | --- | --- | --- | --- | --- | --- | --- | --- | --- | --- | --- | --- | --- | --- | --- | --- | --- | --- | --- | --- | --- | --- | --- | --- | --- | --- | --- | --- | --- | --- | --- | --- | --- | --- | --- | --- | --- | --- | --- | --- | --- | --- | --- | --- | --- | --- | --- | --- | --- | --- | --- | --- | --- | --- | --- | --- | --- | --- | --- | --- | --- | --- | --- | --- | --- | --- | --- | --- | --- | --- | --- | --- | --- | --- | --- | --- | --- | --- | --- | --- | --- | --- | --- | --- | --- | --- | --- | --- | --- | --- | --- | --- | --- | --- | --- | --- | --- | --- | --- | --- | --- | --- | --- | --- | --- | --- | --- | --- | --- | --- | --- | --- | --- | --- | --- | --- | --- | --- | --- | --- | --- | --- | --- | --- | --- | --- | --- | --- | --- | --- | --- | --- | --- | --- | --- | --- | --- | --- | --- | --- | --- | --- | --- | --- | --- | --- | --- | --- | --- | --- | --- | --- | --- | --- | --- | --- | --- | --- | --- | --- | --- | --- | --- | --- | --- | --- | --- | --- | --- | --- | --- | --- | --- | --- | --- | --- | --- | --- | --- | --- | --- | --- | --- | --- | --- | --- | --- | --- | --- | --- | --- | --- | --- | --- | --- | --- | --- | --- | --- | --- | --- | --- | --- | --- | --- | --- | --- | --- | --- | --- | --- | --- | --- | --- | --- | --- | --- | --- | --- | --- | --- | --- | --- | --- | --- | --- | --- | --- | --- | --- | --- | --- | --- | --- | --- | --- | --- | --- | --- | --- | --- | --- | --- | --- | --- | --- | --- | --- | --- | --- | --- | --- | --- | --- | --- | --- | --- | --- | --- | --- | --- | --- | --- | --- | --- | --- | --- | --- | --- | --- | --- | --- | --- | --- | --- | --- | --- | --- | --- | --- | --- | --- | --- | --- | --- | --- | --- | --- | --- | --- | --- | --- | --- | --- | --- | --- | --- | --- | --- | --- | --- | --- | --- | --- | --- | --- | --- | --- | --- | --- | --- | --- | --- | --- | --- | --- | --- | --- | --- | --- | --- | --- | --- | --- | --- | --- | --- | --- | --- | --- | --- | --- | --- | --- | --- | --- | --- | --- | --- | --- | --- | --- | --- | --- | --- | --- | --- | --- | --- | --- | --- | --- | --- | --- | --- | --- | --- | --- | --- | --- | --- | --- | --- | --- | --- | --- | --- | --- | --- | --- | --- | --- | --- | --- | --- | --- | --- | --- | --- | --- | --- | --- | --- | --- | --- | --- | --- | --- | --- | --- | --- | --- | --- | --- | --- | --- | --- | --- | --- | --- | --- | --- | --- | --- | --- | --- | --- | --- | --- | --- | --- | --- | --- | --- | --- | --- | --- | --- | --- | --- | --- | --- | --- | --- | --- | --- | --- | --- | --- | --- | --- | --- | --- | --- | --- | --- | --- | --- | --- | --- | --- | --- | --- | --- | --- | --- | --- | --- | --- | --- | --- | --- | --- | --- | --- | --- | --- | --- | --- | --- | --- | --- | --- | --- | --- | --- | --- | --- | --- | --- | --- | --- | --- | --- | --- | --- | --- | --- | --- | --- | --- | --- | --- | --- | --- | --- | --- | --- | --- | --- | --- | --- | --- | --- | --- | --- | --- | --- | --- | --- | --- | --- | --- | --- | --- | --- | --- | --- | --- | --- | --- | --- | --- | --- | --- | --- | --- | --- | --- | --- | --- | --- | --- | --- | --- | --- | --- | --- | --- | --- | --- | --- | --- | --- | --- | --- | --- | --- | --- | --- | --- | --- | --- | --- | --- | --- | --- | --- | --- | --- | --- | --- | --- | --- | --- | --- | --- | --- | --- | --- | --- | --- | --- | --- | --- | --- | --- | --- | --- | --- | --- | --- | --- | --- | --- | --- | --- | --- | --- | --- | --- | --- | --- | --- | --- | --- | --- | --- | --- | --- | --- | --- | --- | --- | --- | --- | --- | --- | --- | --- | --- | --- | --- | --- | --- | --- | --- | --- | --- | --- | --- | --- | --- | --- | --- | --- | --- | --- | --- | --- | --- | --- | --- | --- | --- | --- | --- | --- | --- | --- | --- | --- | --- | --- | --- | --- | --- | --- | --- | --- | --- | --- | --- | --- | --- | --- | --- | --- | --- | --- | --- | --- | --- | --- | --- | --- | --- | --- | --- | --- | --- | --- | --- | --- | --- | --- | --- | --- | --- | --- | --- | --- | --- | --- | --- | --- | --- | --- | --- | --- | --- | --- | --- | --- | --- | --- | --- | --- | --- | --- | --- | --- | --- | --- | --- | --- | --- | --- | --- | --- | --- | --- | --- | --- | --- | --- | --- | --- | --- | --- | --- | --- | --- | --- | --- | --- | --- | --- | --- | --- | --- | --- | --- | --- | --- | --- | --- | --- | --- | --- | --- | --- | --- | --- | --- | --- | --- | --- | --- | --- | --- | --- | --- | --- | --- | --- | --- | --- | --- | --- | --- | --- | --- | --- | --- | --- | --- | --- | --- | --- | --- | --- | --- | --- | --- | --- | --- | --- | --- | --- | --- | --- | --- | --- | --- | --- | --- | --- | --- | --- | --- | --- | --- | --- | --- | --- | --- | --- | --- | --- | --- | --- | --- | --- | --- | --- | --- | --- | --- | --- | --- | --- | --- | --- | --- | --- | --- | --- | --- | --- | --- | --- | --- | --- | --- | --- | --- | --- | --- | --- | --- | --- | --- | --- | --- | --- | --- | --- | --- | --- | --- | --- | --- | --- | --- | --- | --- | --- | --- | --- | --- | --- | --- | --- | --- | --- | --- | --- | --- | --- | --- | --- | --- | --- | --- | --- | --- | --- | --- | --- | --- | --- | --- | --- | --- | --- | --- | --- | --- | --- | --- | --- | --- | --- | --- | --- | --- | --- | --- | --- | --- | --- | --- | --- | --- | --- | --- | --- | --- | --- | --- | --- | --- | --- | --- | --- | --- | --- | --- | --- | --- | --- | --- | --- | --- | --- | --- | --- | --- | --- | --- | --- | --- | --- | --- | --- | --- | --- | --- | --- | --- | --- | --- | --- | --- | --- | --- | --- | --- | --- | --- | --- | --- | --- | --- | --- | --- | --- | --- | --- | --- | --- | --- | --- | --- | --- | --- | --- | --- | --- | --- | --- | --- | --- | --- | --- | --- | --- | --- | --- | --- | --- | --- | --- | --- | --- | --- | --- | --- | --- | --- | --- | --- | --- | --- | --- | --- | --- | --- | --- | --- | --- | --- | --- | --- | --- | --- | --- | --- | --- | --- | --- | --- | --- | --- | --- | --- | --- | --- | --- | --- | --- | --- | --- | --- | --- | --- | --- | --- | --- | --- | --- | --- | --- | --- | --- | --- | --- | --- | --- | --- | --- | --- | --- | --- | --- | --- | --- | --- | --- | --- | --- | --- | --- | --- | --- | --- | --- | --- | --- | --- | --- | --- | --- | --- | --- | --- | --- | --- | --- | --- | --- | --- | --- | --- | --- | --- | --- | --- | --- | --- | --- | --- | --- | --- | --- | --- | --- | --- | --- | --- | --- | --- | --- | --- | --- | --- | --- | --- | --- | --- | --- | --- | --- | --- | --- | --- | --- | --- | --- | --- | --- | --- | --- | --- | --- | --- | --- | --- | --- | --- | --- | --- | --- | --- | --- | --- | --- | --- | --- | --- | --- | --- | --- | --- | --- | --- | --- | --- | --- | --- | --- | --- | --- | --- | --- | --- | --- | --- | --- | --- | --- | --- | --- | --- | --- | --- | --- | --- | --- | --- | --- | --- | --- | --- | --- | --- | --- | --- | --- | --- | --- | --- | --- | --- | --- | --- | --- | --- | --- | --- | --- | --- | --- | --- | --- | --- | --- | --- | --- | --- | --- | --- | --- | --- | --- | --- | --- | --- | --- | --- | --- | --- | --- | --- | --- | --- | --- | --- | --- | --- | --- | --- | --- | --- | --- | --- | --- | --- | --- | --- | --- | --- | --- | --- | --- | --- | --- | --- | --- | --- | --- | --- | --- | --- | --- | --- | --- | --- | --- | --- | --- | --- | --- | --- | --- | --- | --- | --- | --- | --- | --- | --- | --- | --- | --- | --- | --- | --- | --- | --- | --- | --- | --- | --- | --- | --- | --- | --- | --- | --- | --- | --- | --- | --- | --- | --- | --- | --- | --- | --- | --- | --- | --- | --- | --- | --- | --- | --- | --- | --- | --- | --- | --- | --- | --- | --- | --- | --- | --- | --- | --- | --- | --- | --- | --- | --- | --- | --- | --- | --- | --- | --- | --- | --- | --- | --- | --- | --- | --- | --- | --- | --- | --- | --- | --- | --- | --- | --- | --- | --- | --- | --- | --- | --- | --- | --- | --- | --- | --- | --- | --- | --- | --- | --- | --- | --- | --- | --- | --- | --- | --- | --- | --- | --- | --- | --- | --- | --- | --- | --- | --- | --- | --- | --- | --- | --- | --- | --- | --- | --- | --- | --- | --- | --- | --- | --- | --- | --- | --- | --- | --- | --- | --- | --- | --- | --- | --- | --- | --- | --- | --- | --- | --- | --- | --- | --- | --- | --- | --- | --- | --- | --- | --- | --- | --- | --- | --- | --- | --- | --- | --- | --- | --- | --- | --- | --- | --- | --- | --- | --- | --- | --- | --- | --- | --- | --- | --- | --- | --- | --- | --- | --- | --- | --- | --- | --- | --- | --- | --- | --- | --- | --- | --- | --- | --- | --- | --- | --- | --- | --- | --- | --- | --- | --- | --- | --- | --- | --- | --- | --- | --- | --- | --- | --- | --- | --- | --- | --- | --- | --- | --- | --- | --- | --- | --- | --- | --- | --- | --- | --- | --- | --- | --- | --- | --- | --- | --- | --- | --- | --- | --- | --- | --- | --- | --- | --- | --- | --- | --- | --- | --- | --- | --- | --- | --- | --- | --- | --- | --- | --- | --- | --- | --- | --- | --- | --- | --- | --- | --- | --- | --- | --- | --- | --- | --- | --- | --- | --- | --- | --- | --- | --- | --- | --- | --- | --- | --- | --- | --- | --- | --- | --- | --- | --- | --- | --- | --- | --- | --- | --- | --- | --- | --- | --- | --- | --- | --- | --- | --- | --- | --- | --- | --- | --- | --- | --- | --- | --- | --- | --- | --- | --- | --- | --- | --- | --- | --- | --- | --- | --- | --- | --- | --- | --- | --- | --- | --- | --- | --- | --- | --- | --- | --- | --- | --- | --- | --- | --- | --- | --- | --- | --- | --- | --- | --- | --- | --- | --- | --- | --- | --- | --- | --- | --- | --- | --- | --- | --- | --- | --- | --- | --- | --- | --- | --- | --- | --- | --- | --- | --- | --- | --- | --- | --- | --- | --- | --- | --- | --- | --- | --- | --- | --- | --- | --- | --- | --- | --- | --- | --- | --- | --- | --- | --- | --- | --- | --- | --- | --- | --- | --- | --- | --- | --- | --- | --- | --- | --- | --- | --- | --- | --- | --- | --- | --- | --- | --- | --- | --- | --- | --- | --- | --- | --- | --- | --- | --- | --- | --- | --- | --- | --- | --- | --- | --- | --- | --- | --- | --- | --- | --- | --- | --- | --- | --- | --- | --- | --- | --- | --- | --- | --- | --- | --- | --- | --- | --- | --- | --- | --- | --- | --- | --- | --- | --- | --- | --- | --- | --- | --- | --- | --- | --- | --- | --- | --- | --- | --- | --- | --- | --- | --- | --- | --- | --- | --- | --- | --- | --- | --- | --- | --- | --- | --- | --- | --- | --- | --- | --- | --- | --- | --- | --- | --- | --- | --- | --- | --- | --- | --- | --- | --- | --- | --- | --- | --- | --- | --- | --- | --- | --- | --- | --- | --- | --- | --- | --- | --- | --- | --- | --- | --- | --- | --- | --- | --- | --- | --- | --- | --- | --- | --- | --- | --- | --- | --- | --- | --- | --- | --- | --- | --- | --- | --- | --- | --- | --- | --- | --- | --- | --- | --- | --- | --- | --- | --- | --- | --- | --- | --- | --- | --- | --- | --- | --- | --- | --- | --- | --- | --- | --- | --- | --- | --- | --- | --- | --- | --- | --- | --- | --- | --- | --- | --- | --- | --- | --- | --- | --- | --- | --- | --- | --- | --- | --- | --- | --- | --- | --- | --- | --- | --- | --- | --- | --- | --- | --- | --- | --- | --- | --- | --- | --- | --- | --- | --- | --- | --- | --- | --- | --- | --- | --- | --- | --- | --- | --- | --- | --- | --- | --- | --- | --- | --- | --- | --- | --- | --- | --- | --- | --- | --- | --- | --- | --- | --- | --- | --- | --- | --- | --- | --- | --- | --- | --- | --- | --- | --- | --- | --- | --- | --- | --- | --- | --- | --- | --- | --- | --- | --- | --- | --- | --- | --- | --- | --- | --- | --- | --- | --- | --- | --- | --- | --- | --- | --- | --- | --- | --- | --- | --- | --- | --- | --- | --- | --- | --- | --- | --- | --- | --- | --- | --- | --- | --- | --- | --- | --- | --- | --- | --- | --- | --- | --- | --- | --- | --- | --- | --- | --- | --- | --- | --- | --- | --- | --- | --- | --- | --- | --- | --- | --- | --- | --- | --- | --- | --- | --- | --- | --- | --- | --- | --- | --- | --- | --- | --- | --- | --- | --- | --- | --- | --- | --- | --- | --- | --- | --- | --- | --- | --- | --- | --- | --- | --- | --- | --- | --- | --- | --- | --- | --- | --- | --- | --- | --- | --- | --- | --- | --- | --- | --- | --- | --- | --- | --- | --- | --- | --- | --- | --- | --- | --- | --- | --- | --- | --- | --- | --- | --- | --- | --- | --- | --- | --- | --- | --- | --- | --- | --- | --- | --- | --- | --- | --- | --- | --- | --- | --- | --- | --- | --- | --- | --- | --- | --- | --- | --- | --- | --- | --- | --- | --- | --- | --- | --- | --- | --- | --- | --- | --- | --- | --- | --- | --- | --- | --- | --- | --- | --- | --- | --- | --- | --- | --- | --- | --- | --- | --- | --- | --- | --- | --- | --- | --- | --- | --- | --- | --- | --- | --- | --- | --- | --- | --- | --- | --- | --- | --- | --- | --- | --- | --- | --- | --- | --- | --- | --- | --- | --- | --- | --- | --- | --- | --- | --- | --- | --- | --- | --- | --- | --- | --- | --- | --- | --- | --- | --- | --- | --- | --- | --- | --- | --- | --- | --- | --- | --- | --- | --- | --- | --- | --- | --- | --- | --- | --- | --- | --- | --- | --- | --- | --- | --- | --- | --- | --- | --- | --- | --- | --- | --- | --- | --- | --- | --- | --- | --- | --- | --- | --- | --- | --- | --- | --- | --- | --- | --- | --- | --- | --- | --- | --- | --- | --- | --- | --- | --- | --- | --- | --- | --- | --- | --- | --- | --- | --- | --- | --- | --- | --- | --- | --- | --- | --- | --- | --- | --- | --- | --- | --- | --- | --- | --- | --- | --- | --- | --- | --- | --- | --- | --- | --- | --- | --- | --- | --- | --- | --- | --- | --- | --- | --- | --- | --- | --- | --- | --- | --- | --- | --- | --- | --- | --- | --- | --- | --- | --- | --- | --- | --- | --- | --- | --- | --- | --- | --- | --- | --- | --- | --- | --- | --- | --- | --- | --- | --- | --- | --- | --- | --- | --- | --- | --- | --- | --- | --- | --- | --- | --- | --- | --- | --- | --- | --- | --- | --- | --- | --- | --- | --- | --- | --- | --- | --- | --- | --- | --- | --- | --- | --- | --- | --- | --- | --- | --- | --- | --- | --- | --- | --- | --- | --- | --- | --- | --- | --- | --- | --- | --- | --- | --- | --- | --- | --- | --- | --- | --- | --- | --- | --- | --- | --- | --- | --- | --- | --- | --- | --- | --- | --- | --- | --- | --- | --- | --- | --- | --- | --- | --- | --- | --- | --- | --- | --- | --- | --- | --- | --- | --- | --- | --- | --- | --- | --- | --- | --- | --- | --- | --- | --- | --- | --- | --- | --- | --- | --- | --- | --- | --- | --- | --- | --- | --- | --- | --- | --- | --- | --- | --- | --- | --- | --- | --- | --- | --- | --- | --- | --- | --- | --- | --- | --- | --- | --- | --- | --- | --- | --- | --- | --- | --- | --- | --- | --- | --- | --- | --- | --- | --- | --- | --- | --- | --- | --- | --- | --- | --- | --- | --- | --- | --- | --- | --- | --- | --- | --- | --- | --- | --- | --- | --- | --- | --- | --- | --- | --- | --- | --- | --- | --- | --- | --- | --- | --- | --- | --- | --- | --- | --- | --- | --- | --- | --- | --- | --- | --- | --- | --- | --- | --- | --- | --- | --- | --- | --- | --- | --- | --- | --- | --- | --- | --- | --- | --- | --- | --- | --- | --- | --- | --- | --- | --- | --- | --- | --- | --- | --- | --- | --- | --- | --- | --- | --- | --- | --- | --- | --- | --- | --- | --- | --- | --- | --- | --- | --- | --- | --- | --- | --- | --- | --- | --- | --- | --- | --- | --- | --- | --- | --- | --- | --- | --- | --- | --- | --- | --- | --- | --- | --- | --- | --- | --- | --- | --- | --- | --- | --- | --- | --- | --- | --- | --- | --- | --- | --- | --- | --- | --- | --- | --- | --- | --- | --- | --- | --- | --- | --- | --- | --- | --- | --- | --- | --- | --- | --- | --- | --- | --- | --- | --- | --- | --- | --- | --- | --- | --- | --- | --- | --- | --- | --- | --- | --- | --- | --- | --- | --- | --- | --- | --- | --- | --- | --- | --- | --- | --- | --- | --- | --- | --- | --- | --- | --- | --- | --- | --- | --- | --- | --- | --- | --- | --- | --- | --- | --- | --- | --- | --- | --- | --- | --- | --- | --- | --- | --- | --- | --- | --- | --- | --- | --- | --- | --- | --- | --- | --- | --- | --- | --- | --- | --- | --- | --- | --- | --- | --- | --- | --- | --- | --- | --- | --- | --- | --- | --- | --- | --- | --- | --- | --- | --- | --- | --- | --- | --- | --- | --- | --- | --- | --- | --- | --- | --- | --- | --- | --- | --- | --- | --- | --- | --- | --- | --- | --- | --- | --- | --- | --- | --- | --- | --- | --- | --- | --- | --- | --- | --- | --- | --- | --- | --- | --- | --- | --- | --- | --- | --- | --- | --- | --- | --- | --- | --- | --- | --- | --- | --- | --- | --- | --- | --- | --- | --- | --- | --- | --- | --- | --- | --- | --- | --- | --- | --- | --- | --- | --- | --- | --- | --- | --- | --- | --- | --- | --- | --- | --- | --- | --- | --- | --- | --- | --- | --- | --- | --- | --- | --- | --- | --- | --- | --- | --- | --- | --- | --- | --- | --- | --- | --- | --- | --- | --- | --- | --- | --- | --- | --- | --- | --- | --- | --- | --- | --- | --- | --- | --- | --- | --- | --- | --- | --- | --- | --- | --- | --- | --- | --- | --- | --- | --- | --- | --- | --- | --- | --- | --- | --- | --- | --- | --- | --- | --- | --- | --- | --- | --- | --- | --- | --- | --- | --- | --- | --- | --- | --- | --- | --- | --- | --- | --- | --- | --- | --- | --- | --- | --- | --- | --- | --- | --- | --- | --- | --- | --- | --- | --- | --- | --- | --- | --- | --- | --- | --- | --- | --- | --- | --- | --- | --- | --- | --- | --- | --- | --- | --- | --- | --- | --- | --- | --- | --- | --- | --- | --- | --- | --- | --- | --- | --- | --- | --- | --- | --- | --- | --- | --- | --- | --- | --- | --- | --- | --- | --- | --- | --- | --- | --- | --- | --- | --- | --- | --- | --- | --- | --- | --- | --- | --- | --- | --- | --- | --- | --- | --- | --- | --- | --- | --- | --- | --- | --- | --- | --- | --- | --- | --- | --- | --- | --- | --- | --- | --- | --- | --- | --- | --- | --- | --- | --- | --- | --- | --- | --- | --- | --- | --- | --- | --- | --- | --- | --- | --- | --- | --- | --- | --- | --- | --- | --- | --- | --- | --- | --- | --- | --- | --- | --- | --- | --- | --- | --- | --- | --- | --- | --- | --- | --- | --- | --- | --- | --- | --- | --- | --- | --- | --- | --- | --- | --- | --- | --- | --- | --- | --- | --- | --- | --- | --- | --- | --- | --- | --- | --- | --- | --- | --- | --- | --- | --- | --- | --- | --- | --- | --- | --- | --- | --- | --- | --- | --- | --- | --- | --- | --- | --- | --- | --- | --- | --- | --- | --- | --- | --- | --- | --- | --- | --- | --- | --- | --- | --- | --- | --- | --- | --- | --- | --- | --- | --- | --- | --- | --- | --- | --- | --- | --- | --- | --- | --- | --- | --- | --- | --- | --- | --- | --- | --- | --- | --- | --- | --- | --- | --- | --- | --- | --- | --- | --- | --- | --- | --- | --- | --- | --- | --- | --- | --- | --- | --- | --- | --- | --- | --- | --- | --- | --- | --- | --- | --- | --- | --- | --- | --- | --- | --- | --- | --- | --- | --- | --- | --- | --- | --- | --- | --- | --- | --- | --- | --- | --- | --- | --- | --- | --- | --- | --- | --- | --- | --- | --- | --- | --- | --- | --- | --- | --- | --- | --- | --- | --- | --- | --- | --- | --- | --- | --- | --- | --- | --- | --- | --- | --- | --- | --- | --- | --- | --- | --- | --- | --- | --- | --- | --- | --- | --- | --- | --- | --- | --- | --- | --- | --- | --- | --- | --- | --- | --- | --- | --- | --- | --- | --- | --- | --- | --- | --- | --- | --- | --- | --- | --- | --- | --- | --- | --- | --- | --- | --- | --- | --- | --- | --- | --- | --- | --- | --- | --- | --- | --- | --- | --- | --- | --- | --- | --- | --- | --- | --- | --- | --- | --- | --- | --- | --- | --- | --- | --- | --- | --- | --- | --- | --- | --- | --- | --- | --- | --- | --- | --- | --- | --- | --- | --- | --- | --- | --- | --- | --- | --- | --- | --- | --- | --- | --- | --- | --- | --- | --- | --- | --- | --- | --- | --- | --- | --- | --- | --- | --- | --- | --- | --- | --- | --- | --- | --- | --- | --- | --- | --- | --- | --- | --- | --- | --- | --- | --- | --- | --- | --- | --- | --- | --- | --- | --- | --- | --- | --- | --- | --- | --- | --- | --- | --- | --- | --- | --- | --- | --- | --- | --- | --- | --- | --- | --- | --- | --- | --- | --- | --- | --- | --- | --- | --- | --- | --- | --- | --- | --- | --- | --- | --- | --- | --- | --- | --- | --- | --- | --- | --- | --- | --- | --- | --- | --- | --- | --- | --- | --- | --- | --- | --- | --- | --- | --- | --- | --- | --- | --- | --- | --- | --- | --- | --- | --- | --- | --- | --- | --- | --- | --- | --- | --- | --- | --- | --- | --- | --- | --- | --- | --- | --- | --- | --- | --- | --- | --- | --- | --- | --- | --- | --- | --- | --- | --- | --- | --- | --- | --- | --- | --- | --- | --- | --- | --- | --- | --- | --- | --- | --- | --- | --- | --- | --- | --- | --- | --- | --- | --- | --- | --- | --- | --- | --- | --- | --- | --- | --- | --- | --- | --- | --- | --- | --- | --- | --- | --- | --- | --- | --- | --- | --- | --- | --- | --- | --- | --- | --- | --- | --- | --- | --- | --- | --- | --- | --- | --- | --- | --- | --- | --- | --- | --- | --- | --- | --- | --- | --- | --- | --- | --- | --- | --- | --- | --- | --- | --- | --- | --- | --- | --- | --- | --- | --- | --- | --- | --- | --- | --- | --- | --- | --- | --- | --- | --- | --- | --- | --- | --- | --- | --- | --- | --- | --- | --- | --- | --- | --- | --- | --- | --- | --- | --- | --- | --- | --- | --- | --- | --- | --- | --- | --- | --- | --- | --- | --- | --- | --- | --- | --- | --- | --- | --- | --- | --- | --- | --- | --- | --- | --- | --- | --- | --- | --- | --- | --- | --- | --- | --- | --- | --- | --- | --- | --- | --- | --- | --- | --- | --- | --- | --- | --- | --- | --- | --- | --- | --- | --- | --- | --- | --- | --- | --- | --- | --- | --- | --- | --- | --- | --- | --- | --- | --- | --- | --- | --- | --- | --- | --- | --- | --- | --- | --- | --- | --- | --- | --- | --- | --- | --- | --- | --- | --- | --- | --- | --- | --- | --- | --- | --- | --- | --- | --- | --- | --- | --- | --- | --- | --- | --- | --- | --- | --- | --- | --- | --- | --- | --- | --- | --- | --- | --- | --- | --- | --- | --- | --- | --- | --- | --- | --- | --- | --- | --- | --- | --- | --- | --- | --- | --- | --- | --- | --- | --- | --- | --- | --- | --- | --- | --- | --- | --- | --- | --- | --- | --- | --- | --- | --- | --- | --- | --- | --- | --- | --- | --- | --- | --- | --- | --- | --- | --- | --- | --- | --- | --- | --- | --- | --- | --- | --- | --- | --- | --- | --- | --- | --- | --- | --- | --- | --- | --- | --- | --- | --- | --- | --- | --- | --- | --- | --- | --- | --- | --- | --- | --- | --- | --- | --- | --- | --- | --- | --- | --- | --- | --- | --- | --- | --- | --- | --- | --- | --- | --- | --- | --- | --- | --- | --- | --- | --- | --- | --- | --- | --- | --- | --- | --- | --- | --- | --- | --- | --- | --- | --- | --- | --- | --- | --- | --- | --- | --- | --- | --- | --- | --- | --- | --- | --- | --- | --- | --- | --- | --- | --- | --- | --- | --- | --- | --- | --- | --- | --- | --- | --- | --- | --- | --- | --- | --- | --- | --- | --- | --- | --- | --- | --- | --- | --- | --- | --- | --- | --- | --- | --- | --- | --- | --- | --- | --- | --- | --- | --- | --- | --- | --- | --- | --- | --- | --- | --- | --- | --- | --- | --- | --- | --- | --- | --- | --- | --- | --- | --- | --- | --- | --- | --- | --- | --- | --- | --- | --- | --- | --- | --- | --- | --- | --- | --- | --- | --- | --- | --- | --- | --- | --- | --- | --- | --- | --- | --- | --- | --- | --- | --- | --- | --- | --- | --- | --- | --- | --- | --- | --- | --- | --- | --- | --- | --- | --- | --- | --- | --- | --- | --- | --- | --- | --- | --- | --- | --- | --- | --- | --- | --- | --- | --- | --- | --- | --- | --- | --- | --- | --- | --- | --- | --- | --- | --- | --- | --- | --- | --- | --- | --- | --- | --- | --- | --- | --- | --- | --- | --- | --- | --- | --- | --- | --- | --- | --- | --- | --- | --- | --- | --- | --- | --- | --- | --- | --- | --- | --- | --- | --- | --- | --- | --- | --- | --- | --- | --- | --- | --- | --- | --- | --- | --- | --- | --- | --- | --- | --- | --- | --- | --- | --- | --- | --- | --- | --- | --- | --- | --- | --- | --- | --- | --- | --- | --- | --- | --- | --- | --- | --- | --- | --- | --- | --- | --- | --- | --- | --- | --- | --- | --- | --- | --- | --- | --- | --- | --- | --- | --- | --- | --- | --- | --- | --- | --- | --- | --- | --- | --- | --- | --- | --- | --- | --- | --- | --- | --- | --- | --- | --- | --- | --- | --- | --- | --- | --- | --- | --- | --- | --- | --- | --- | --- | --- | --- | --- | --- | --- | --- | --- | --- | --- | --- | --- | --- | --- | --- | --- | --- | --- | --- | --- | --- | --- | --- | --- | --- | --- | --- | --- | --- | --- | --- | --- | --- | --- | --- | --- | --- | --- | --- | --- | --- | --- | --- | --- | --- | --- | --- | --- | --- | --- | --- | --- | --- | --- | --- | --- | --- | --- | --- | --- | --- | --- | --- | --- | --- | --- | --- | --- | --- | --- | --- | --- | --- | --- | --- | --- | --- | --- | --- | --- | --- | --- | --- | --- | --- | --- | --- | --- | --- | --- | --- | --- | --- | --- | --- | --- | --- | --- | --- | --- | --- | --- | --- | --- | --- | --- | --- | --- | --- | --- | --- | --- | --- | --- | --- | --- | --- | --- | --- | --- | --- | --- | --- | --- | --- | --- | --- | --- | --- | --- | --- | --- | --- | --- | --- | --- | --- | --- | --- | --- | --- | --- | --- | --- | --- | --- | --- | --- | --- | --- | --- | --- | --- | --- | --- | --- | --- | --- | --- | --- | --- | --- | --- | --- | --- | --- | --- | --- | --- | --- | --- | --- | --- | --- | --- | --- | --- | --- | --- | --- | --- | --- | --- | --- | --- | --- | --- | --- | --- | --- | --- | --- | --- | --- | --- | --- | --- | --- | --- | --- | --- | --- | --- | --- | --- | --- | --- | --- | --- | --- | --- | --- | --- | --- | --- | --- | --- | --- | --- | --- | --- | --- | --- | --- | --- | --- | --- | --- | --- | --- | --- | --- | --- | --- | --- | --- | --- | --- | --- | --- | --- | --- | --- | --- | --- | --- | --- | --- | --- | --- | --- | --- | --- | --- | --- | --- | --- | --- | --- | --- | --- | --- | --- | --- | --- | --- | --- | --- | --- | --- | --- | --- | --- | --- | --- | --- | --- | --- | --- | --- | --- | --- | --- | --- | --- | --- | --- | --- | --- | --- | --- | --- | --- | --- | --- | --- | --- | --- | --- | --- | --- | --- | --- | --- | --- | --- | --- | --- | --- | --- | --- | --- | --- | --- | --- | --- | --- | --- | --- | --- | --- | --- | --- | --- | --- | --- | --- | --- | --- | --- | --- | --- | --- | --- | --- | --- | --- | --- | --- | --- | --- | --- | --- | --- | --- | --- | --- | --- | --- | --- | --- | --- | --- | --- | --- | --- | --- | --- | --- | --- | --- | --- | --- | --- | --- | --- | --- | --- | --- | --- | --- | --- | --- | --- | --- | --- | --- | --- | --- | --- | --- | --- | --- | --- | --- | --- | --- | --- | --- | --- | --- | --- | --- | --- | --- | --- | --- | --- | --- | --- | --- | --- | --- | --- | --- | --- | --- | --- | --- | --- | --- | --- | --- | --- | --- | --- | --- | --- | --- | --- | --- | --- | --- | --- | --- | --- | --- | --- | --- | --- | --- | --- | --- | --- | --- | --- | --- | --- | --- | --- | --- | --- | --- | --- | --- | --- | --- | --- | --- | --- | --- | --- | --- | --- | --- | --- | --- | --- | --- | --- | --- | --- | --- | --- | --- | --- | --- | --- | --- | --- | --- | --- | --- | --- | --- | --- | --- | --- | --- | --- | --- | --- | --- | --- | --- | --- | --- | --- | --- | --- | --- | --- | --- | --- | --- | --- | --- | --- | --- | --- | --- | --- | --- | --- | --- | --- | --- | --- | --- | --- | --- | --- | --- | --- | --- | --- | --- | --- | --- | --- | --- | --- | --- | --- | --- | --- | --- | --- | --- | --- | --- | --- | --- | --- | --- | --- | --- | --- | --- | --- | --- | --- | --- | --- | --- | --- | --- | --- | --- | --- | --- | --- | --- | --- | --- | --- | --- | --- | --- | --- | --- | --- | --- | --- | --- | --- | --- | --- | --- | --- | --- | --- | --- | --- | --- | --- | --- | --- | --- | --- | --- | --- | --- | --- | --- | --- | --- | --- | --- | --- | --- | --- | --- | --- | --- | --- | --- | --- | --- | --- | --- | --- | --- | --- | --- | --- | --- | --- | --- | --- | --- | --- | --- | --- | --- | --- | --- | --- | --- | --- | --- | --- | --- | --- | --- | --- | --- | --- | --- | --- | --- | --- | --- | --- | --- | --- | --- | --- | --- | --- | --- | --- | --- | --- | --- | --- | --- | --- | --- | --- | --- | --- | --- | --- | --- | --- | --- | --- | --- | --- | --- | --- | --- | --- | --- | --- | --- | --- | --- | --- | --- | --- | --- | --- | --- | --- | --- | --- | --- | --- | --- | --- | --- | --- | --- | --- | --- | --- | --- | --- | --- | --- | --- | --- | --- | --- | --- | --- | --- | --- | --- | --- | --- | --- | --- | --- | --- | --- | --- | --- | --- | --- | --- | --- | --- | --- | --- | --- | --- | --- | --- | --- | --- | --- | --- | --- | --- | --- | --- | --- | --- | --- | --- | --- | --- | --- | --- | --- | --- | --- | --- | --- | --- | --- | --- | --- | --- | --- | --- | --- | --- | --- | --- | --- | --- | --- | --- | --- | --- | --- | --- | --- | --- | --- | --- | --- | --- | --- | --- | --- | --- | --- | --- | --- | --- | --- | --- | --- | --- | --- | --- | --- | --- | --- | --- | --- | --- | --- | --- | --- | --- | --- | --- | --- | --- | --- | --- | --- | --- | --- | --- | --- | --- | --- | --- | --- | --- | --- | --- | --- | --- | --- | --- | --- | --- | --- | --- | --- | --- | --- | --- | --- | --- | --- | --- | --- | --- | --- | --- | --- | --- | --- | --- | --- | --- | --- | --- | --- | --- | --- | --- | --- | --- | --- | --- | --- | --- | --- | --- | --- | --- | --- | --- | --- | --- | --- | --- | --- | --- | --- | --- | --- | --- | --- | --- | --- | --- | --- | --- | --- | --- | --- | --- | --- | --- | --- | --- | --- | --- | --- | --- | --- | --- | --- | --- | --- | --- | --- | --- | --- | --- | --- | --- | --- | --- | --- | --- | --- | --- | --- | --- | --- | --- | --- | --- | --- | --- | --- | --- | --- | --- | --- | --- | --- | --- | --- | --- | --- | --- | --- | --- | --- | --- | --- | --- | --- | --- | --- | --- | --- | --- | --- | --- | --- | --- | --- | --- | --- | --- | --- | --- | --- | --- | --- | --- | --- | --- | --- | --- | --- | --- | --- | --- | --- | --- | --- | --- | --- | --- | --- | --- | --- | --- | --- | --- | --- | --- | --- | --- | --- | --- | --- | --- | --- | --- | --- | --- | --- | --- | --- | --- | --- | --- | --- | --- | --- | --- | --- | --- | --- | --- | --- | --- | --- | --- | --- | --- | --- | --- | --- | --- | --- | --- | --- | --- | --- | --- | --- | --- | --- | --- | --- | --- | --- | --- | --- | --- | --- | --- | --- | --- | --- | --- | --- | --- | --- | --- | --- | --- | --- | --- | --- | --- | --- | --- | --- | --- | --- | --- | --- | --- | --- | --- | --- | --- | --- | --- | --- | --- | --- | --- | --- | --- | --- | --- | --- | --- | --- | --- | --- | --- | --- | --- | --- | --- | --- | --- | --- | --- | --- | --- | --- | --- | --- | --- | --- | --- | --- | --- | --- | --- | --- | --- | --- | --- | --- | --- | --- | --- | --- | --- | --- | --- | --- | --- | --- | --- | --- | --- | --- | --- | --- | --- | --- | --- | --- | --- | --- | --- | --- | --- | --- | --- | --- | --- | --- | --- | --- | --- | --- | --- | --- | --- | --- | --- | --- | --- | --- | --- | --- | --- | --- | --- | --- | --- | --- | --- | --- | --- | --- | --- | --- | --- | --- | --- | --- | --- | --- | --- | --- | --- | --- | --- | --- | --- | --- | --- | --- | --- | --- | --- | --- | --- | --- | --- | --- | --- | --- | --- | --- | --- | --- | --- | --- | --- | --- | --- | --- | --- | --- | --- | --- | --- | --- | --- | --- | --- | --- | --- | --- | --- | --- | --- | --- | --- | --- | --- | --- | --- | --- | --- | --- | --- | --- | --- | --- | --- | --- | --- | --- | --- | --- | --- | --- | --- | --- | --- | --- | --- | --- | --- | --- | --- | --- | --- | --- | --- | --- | --- | --- | --- | --- | --- | --- | --- | --- | --- | --- | --- | --- | --- | --- | --- | --- | --- | --- | --- | --- | --- | --- | --- | --- | --- | --- | --- | --- | --- | --- | --- | --- | --- | --- | --- | --- | --- | --- | --- | --- | --- | --- | --- | --- | --- | --- | --- | --- | --- | --- | --- | --- | --- | --- | --- | --- | --- | --- | --- | --- | --- | --- | --- | --- | --- | --- | --- | --- | --- | --- | --- | --- | --- | --- | --- | --- | --- | --- | --- | --- | --- | --- | --- | --- | --- | --- | --- | --- | --- | --- | --- | --- | --- | --- | --- | --- | --- | --- | --- | --- | --- | --- | --- | --- | --- | --- | --- | --- | --- | --- | --- | --- | --- | --- | --- | --- | --- | --- | --- | --- | --- | --- | --- | --- | --- | --- | --- | --- | --- | --- | --- | --- | --- | --- | --- | --- | --- | --- | --- | --- | --- | --- | --- | --- | --- | --- | --- | --- | --- | --- | --- | --- | --- | --- | --- | --- | --- | --- | --- | --- | --- | --- | --- | --- | --- | --- | --- | --- | --- | --- | --- | --- | --- | --- | --- | --- | --- | --- | --- | --- | --- | --- | --- | --- | --- | --- | --- | --- | --- | --- | --- | --- | --- | --- | --- | --- | --- | --- | --- | --- | --- | --- | --- | --- | --- | --- | --- | --- | --- | --- | --- | --- | --- | --- | --- | --- | --- | --- | --- | --- | --- | --- | --- | --- | --- | --- | --- | --- | --- | --- | --- | --- | --- | --- | --- | --- | --- | --- | --- | --- | --- | --- | --- | --- | --- | --- | --- | --- | --- | --- | --- | --- | --- | --- | --- | --- | --- | --- | --- | --- | --- | --- | --- | --- | --- | --- | --- | --- | --- | --- | --- | --- | --- | --- | --- | --- | --- | --- | --- | --- | --- | --- | --- | --- | --- | --- | --- | --- | --- | --- | --- | --- | --- | --- | --- | --- | --- | --- | --- | --- | --- | --- | --- | --- | --- | --- | --- | --- | --- | --- | --- | --- | --- | --- | --- | --- | --- | --- | --- | --- | --- | --- | --- | --- | --- | --- | --- | --- | --- | --- | --- | --- | --- | --- | --- | --- | --- | --- | --- | --- | --- | --- | --- | --- | --- | --- | --- | --- | --- | --- | --- | --- | --- | --- | --- | --- | --- | --- | --- | --- | --- | --- | --- | --- | --- | --- | --- | --- | --- | --- | --- | --- | --- | --- | --- | --- | --- | --- | --- | --- | --- | --- | --- | --- | --- | --- | --- | --- | --- | --- | --- | --- | --- | --- | --- | --- | --- | --- | --- | --- | --- | --- | --- | --- | --- | --- | --- | --- | --- | --- | --- | --- | --- | --- | --- | --- | --- | --- | --- | --- | --- | --- | --- | --- | --- | --- | --- | --- | --- | --- | --- | --- | --- | --- | --- | --- | --- | --- | --- | --- | --- | --- | --- | --- | --- | --- | --- | --- | --- | --- | --- | --- | --- | --- | --- | --- | --- | --- | --- | --- | --- | --- | --- | --- | --- | --- | --- | --- | --- | --- | --- | --- | --- | --- | --- | --- | --- | --- | --- | --- | --- | --- | --- | --- | --- | --- | --- | --- | --- | --- | --- | --- | --- | --- | --- | --- | --- | --- | --- | --- | --- | --- | --- | --- | --- | --- | --- | --- | --- | --- | --- | --- | --- | --- | --- | --- | --- | --- | --- | --- | --- | --- | --- | --- | --- | --- | --- | --- | --- | --- | --- | --- | --- | --- | --- | --- | --- | --- | --- | --- | --- | --- | --- | --- | --- | --- | --- | --- | --- | --- | --- | --- | --- | --- | --- | --- | --- | --- | --- | --- | --- | --- | --- | --- | --- | --- | --- | --- | --- | --- | --- | --- | --- | --- | --- | --- | --- | --- | --- | --- | --- | --- | --- | --- | --- | --- | --- | --- | --- | --- | --- | --- | --- | --- | --- | --- | --- | --- | --- | --- | --- | --- | --- | --- | --- | --- | --- | --- | --- | --- | --- | --- | --- | --- | --- | --- | --- | --- | --- | --- | --- | --- | --- | --- | --- | --- | --- | --- | --- | --- | --- | --- | --- | --- | --- | --- | --- | --- | --- | --- | --- | --- | --- | --- | --- | --- | --- | --- | --- | --- | --- | --- | --- | --- | --- | --- | --- | --- | --- | --- | --- | --- | --- | --- | --- | --- | --- | --- | --- | --- | --- | --- | --- | --- | --- | --- | --- | --- | --- | --- | --- | --- | --- | --- | --- | --- | --- | --- | --- | --- | --- | --- | --- | --- | --- | --- | --- | --- | --- | --- | --- | --- | --- | --- | --- | --- | --- | --- | --- | --- | --- | --- | --- | --- | --- | --- | --- | --- | --- | --- | --- | --- | --- | --- | --- | --- | --- | --- | --- | --- | --- | --- | --- | --- | --- | --- | --- | --- | --- | --- | --- | --- | --- | --- | --- | --- | --- | --- | --- | --- | --- | --- | --- | --- | --- | --- | --- | --- | --- | --- | --- | --- | --- | --- | --- | --- | --- | --- | --- | --- | --- | --- | --- | --- | --- | --- | --- | --- | --- | --- | --- | --- | --- | --- | --- | --- | --- | --- | --- | --- | --- | --- | --- | --- | --- | --- | --- | --- | --- | --- | --- | --- | --- | --- | --- | --- | --- | --- | --- | --- | --- | --- | --- | --- | --- | --- | --- | --- | --- | --- | --- | --- | --- | --- | --- | --- | --- | --- | --- | --- | --- | --- | --- | --- | --- | --- | --- | --- | --- | --- | --- | --- | --- | --- | --- | --- | --- | --- | --- | --- | --- | --- | --- | --- | --- | --- | --- | --- | --- | --- | --- | --- | --- | --- | --- | --- | --- | --- | --- | --- | --- | --- | --- | --- | --- | --- | --- | --- | --- | --- | --- | --- | --- | --- | --- | --- | --- | --- | --- | --- | --- | --- | --- | --- | --- | --- | --- | --- | --- | --- | --- | --- | --- | --- | --- | --- | --- | --- | --- | --- | --- | --- | --- | --- | --- | --- | --- | --- | --- | --- | --- | --- | --- | --- | --- | --- | --- | --- | --- | --- | --- | --- | --- | --- | --- | --- | --- | --- | --- | --- | --- | --- | --- | --- | --- | --- | --- | --- | --- | --- | --- | --- | --- | --- | --- | --- | --- | --- | --- | --- | --- | --- | --- | --- | --- | --- | --- | --- | --- | --- | --- | --- | --- | --- | --- | --- | --- | --- | --- | --- | --- | --- | --- | --- | --- | --- | --- | --- | --- | --- | --- | --- | --- | --- | --- | --- | --- | --- | --- | --- | --- | --- | --- | --- | --- | --- | --- | --- | --- | --- | --- | --- | --- | --- | --- | --- | --- | --- | --- | --- | --- | --- | --- | --- | --- | --- | --- | --- | --- | --- | --- | --- | --- | --- | --- | --- | --- | --- | --- | --- | --- | --- | --- | --- | --- | --- | --- | --- | --- | --- | --- | --- | --- | --- | --- | --- | --- | --- | --- | --- | --- | --- | --- | --- | --- | --- | --- | --- | --- | --- | --- | --- | --- | --- | --- | --- | --- | --- | --- | --- | --- | --- | --- | --- | --- | --- | --- | --- | --- | --- | --- | --- | --- | --- | --- | --- | --- | --- | --- | --- | --- | --- | --- | --- | --- | --- | --- | --- | --- | --- | --- | --- | --- | --- | --- | --- | --- | --- | --- | --- | --- | --- | --- | --- | --- | --- | --- | --- | --- | --- | --- | --- | --- | --- | --- | --- | --- | --- | --- | --- | --- | --- | --- | --- | --- | --- | --- | --- | --- | --- | --- | --- | --- | --- | --- | --- | --- | --- | --- | --- | --- | --- | --- | --- | --- | --- | --- | --- | --- | --- | --- | --- | --- | --- | --- | --- | --- | --- | --- | --- | --- | --- | --- | --- | --- | --- | --- | --- | --- | --- | --- | --- | --- | --- | --- | --- | --- | --- | --- | --- | --- | --- | --- | --- | --- | --- | --- | --- | --- | --- | --- | --- | --- | --- | --- | --- | --- | --- | --- | --- | --- | --- | --- | --- | --- | --- | --- | --- | --- | --- | --- | --- | --- | --- | --- | --- | --- | --- | --- | --- | --- | --- | --- | --- | --- | --- | --- | --- | --- | --- | --- | --- | --- | --- | --- | --- | --- | --- | --- | --- | --- | --- | --- | --- | --- | --- | --- | --- | --- | --- | --- | --- | --- | --- | --- | --- | --- | --- | --- | --- | --- | --- | --- | --- | --- | --- | --- | --- | --- | --- | --- | --- | --- | --- | --- | --- | --- | --- | --- | --- | --- | --- | --- | --- | --- | --- | --- | --- | --- | --- | --- | --- | --- | --- | --- | --- | --- | --- | --- | --- | --- | --- | --- | --- | --- | --- | --- | --- | --- | --- | --- | --- | --- | --- | --- | --- | --- | --- | --- | --- | --- | --- | --- | --- | --- | --- | --- | --- | --- | --- | --- | --- | --- | --- | --- | --- | --- | --- | --- | --- | --- | --- | --- | --- | --- | --- | --- | --- | --- | --- | --- | --- | --- | --- | --- | --- | --- | --- | --- | --- | --- | --- | --- | --- | --- | --- | --- | --- | --- | --- | --- | --- | --- | --- | --- | --- | --- | --- | --- | --- | --- | --- | --- | --- | --- | --- | --- | --- | --- | --- | --- | --- | --- | --- | --- | --- | --- | --- | --- | --- | --- | --- | --- | --- | --- | --- | --- | --- | --- | --- | --- | --- | --- | --- | --- | --- | --- | --- | --- | --- | --- | --- | --- | --- | --- | --- | --- | --- | --- | --- | --- | --- | --- | --- | --- | --- | --- | --- | --- | --- | --- | --- | --- | --- | --- | --- | --- | --- | --- | --- | --- | --- | --- | --- | --- | --- | --- | --- | --- | --- | --- | --- | --- | --- | --- | --- | --- | --- | --- | --- | --- | --- | --- | --- | --- | --- | --- | --- | --- | --- | --- | --- | --- | --- | --- | --- | --- | --- | --- | --- | --- | --- | --- | --- | --- | --- | --- | --- | --- | --- | --- | --- | --- | --- | --- | --- | --- | --- | --- | --- | --- | --- | --- | --- | --- | --- | --- | --- | --- | --- | --- | --- | --- | --- | --- | --- | --- | --- | --- | --- | --- | --- | --- | --- | --- | --- | --- | --- | --- | --- | --- | --- | --- | --- | --- | --- | --- | --- | --- | --- | --- | --- | --- | --- | --- | --- | --- | --- | --- | --- | --- | --- | --- | --- | --- | --- | --- | --- | --- | --- | --- | --- | --- | --- | --- | --- | --- | --- | --- | --- | --- | --- | --- | --- | --- | --- | --- | --- | --- | --- | --- | --- | --- | --- | --- | --- | --- | --- | --- | --- | --- | --- | --- | --- | --- | --- | --- | --- | --- | --- | --- | --- | --- | --- | --- | --- | --- | --- | --- | --- | --- | --- | --- | --- | --- | --- | --- | --- | --- | --- | --- | --- | --- | --- | --- | --- | --- | --- | --- | --- | --- | --- | --- | --- | --- | --- | --- | --- | --- | --- | --- | --- | --- | --- | --- | --- | --- | --- | --- | --- | --- | --- | --- | --- | --- | --- | --- | --- | --- | --- | --- | --- | --- | --- | --- | --- | --- | --- | --- | --- | --- | --- | --- | --- | --- | --- | --- | --- | --- | --- | --- | --- | --- | --- | --- | --- | --- | --- | --- | --- | --- | --- | --- | --- | --- | --- | --- | --- | --- | --- | --- | --- | --- | --- | --- | --- | --- | --- | --- | --- | --- | --- | --- | --- | --- | --- | --- | --- | --- | --- | --- | --- | --- | --- | --- | --- | --- | --- | --- | --- | --- | --- | --- | --- | --- | --- | --- | --- | --- | --- | --- | --- | --- | --- | --- | --- | --- | --- | --- | --- | --- | --- | --- | --- | --- | --- | --- | --- | --- | --- | --- | --- | --- | --- | --- | --- | --- | --- | --- | --- | --- | --- | --- | --- | --- | --- | --- | --- | --- | --- | --- | --- | --- | --- | --- | --- | --- | --- | --- | --- | --- | --- | --- | --- | --- | --- | --- | --- | --- | --- | --- | --- | --- | --- | --- | --- | --- | --- | --- | --- | --- | --- | --- | --- | --- | --- | --- | --- | --- | --- | --- | --- | --- | --- | --- | --- | --- | --- | --- | --- | --- | --- | --- | --- | --- | --- | --- | --- | --- | --- | --- | --- | --- | --- | --- | --- | --- | --- | --- | --- | --- | --- | --- | --- | --- | --- | --- | --- | --- | --- | --- | --- | --- | --- | --- | --- | --- | --- | --- | --- | --- | --- | --- | --- | --- | --- | --- | --- | --- | --- | --- | --- | --- | --- | --- | --- | --- | --- | --- | --- | --- | --- | --- | --- | --- | --- | --- | --- | --- | --- | --- | --- | --- | --- | --- | --- | --- | --- | --- | --- | --- | --- | --- | --- | --- | --- | --- | --- | --- | --- | --- | --- | --- | --- | --- | --- | --- | --- | --- | --- | --- | --- | --- | --- | --- | --- | --- | --- | --- | --- | --- | --- | --- | --- | --- | --- | --- | --- | --- | --- | --- | --- | --- | --- | --- | --- | --- | --- | --- | --- | --- | --- | --- | --- | --- | --- | --- | --- | --- | --- | --- | --- | --- | --- | --- | --- | --- | --- | --- | --- | --- | --- | --- | --- | --- | --- | --- | --- | --- | --- | --- | --- | --- | --- | --- | --- | --- | --- | --- | --- | --- | --- | --- | --- | --- | --- | --- | --- | --- | --- | --- | --- | --- | --- | --- | --- | --- | --- | --- | --- | --- | --- | --- | --- | --- | --- | --- | --- | --- | --- | --- | --- | --- | --- | --- | --- | --- | --- | --- | --- | --- | --- | --- | --- | --- | --- | --- | --- | --- | --- | --- | --- | --- | --- | --- | --- | --- | --- | --- | --- | --- | --- | --- | --- | --- | --- | --- | --- | --- | --- | --- | --- | --- | --- | --- | --- | --- | --- | --- | --- | --- | --- | --- | --- | --- | --- | --- | --- | --- | --- | --- | --- | --- | --- | --- | --- | --- | --- | --- | --- | --- | --- | --- | --- | --- | --- | --- | --- | --- | --- | --- | --- | --- | --- | --- | --- | --- | --- | --- | --- | --- | --- | --- | --- | --- | --- | --- | --- | --- | --- | --- | --- | --- | --- | --- | --- | --- | --- | --- | --- | --- | --- | --- | --- | --- | --- | --- | --- | --- | --- | --- | --- | --- | --- | --- | --- | --- | --- | --- | --- | --- | --- | --- | --- | --- | --- | --- | --- | --- | --- | --- | --- | --- | --- | --- | --- | --- | --- | --- | --- | --- | --- | --- | --- | --- | --- | --- | --- | --- | --- | --- | --- | --- | --- | --- | --- | --- | --- | --- | --- | --- | --- | --- | --- | --- | --- | --- | --- | --- | --- | --- | --- | --- | --- | --- | --- | --- | --- | --- | --- | --- | --- | --- | --- | --- | --- | --- | --- | --- | --- | --- | --- | --- | --- | --- | --- | --- | --- | --- | --- | --- | --- | --- | --- | --- | --- | --- | --- | --- | --- | --- | --- | --- | --- | --- | --- | --- | --- | --- | --- | --- | --- | --- | --- | --- | --- | --- | --- | --- | --- | --- | --- | --- | --- | --- | --- | --- | --- | --- | --- | --- | --- | --- | --- | --- | --- | --- | --- | --- | --- | --- | --- | --- | --- | --- | --- | --- | --- | --- | --- | --- | --- | --- | --- | --- | --- | --- | --- | --- | --- | --- | --- | --- | --- | --- | --- | --- | --- | --- | --- | --- | --- | --- | --- | --- | --- | --- | --- | --- | --- | --- | --- | --- | --- | --- | --- | --- | --- | --- | --- | --- | --- | --- | --- | --- | --- | --- | --- | --- | --- | --- | --- | --- | --- | --- | --- | --- | --- | --- | --- | --- | --- | --- | --- | --- | --- | --- | --- | --- | --- | --- | --- | --- | --- | --- | --- | --- | --- | --- | --- | --- | --- | --- | --- | --- | --- | --- | --- | --- | --- | --- | --- | --- | --- | --- | --- | --- | --- | --- | --- | --- | --- | --- | --- | --- | --- | --- | --- | --- | --- | --- | --- | --- | --- | --- | --- | --- | --- | --- | --- | --- | --- | --- | --- | --- | --- | --- | --- | --- | --- | --- | --- | --- | --- | --- | --- | --- | --- | --- | --- | --- | --- | --- | --- | --- | --- | --- | --- | --- | --- | --- | --- | --- | --- | --- | --- | --- | --- | --- | --- | --- | --- | --- | --- | --- | --- | --- | --- | --- | --- | --- | --- | --- | --- | --- | --- | --- | --- | --- | --- | --- | --- | --- | --- | --- | --- | --- | --- | --- | --- | --- | --- | --- | --- | --- | --- | --- | --- | --- | --- | --- | --- | --- | --- | --- | --- | --- | --- | --- | --- | --- | --- | --- | --- | --- | --- | --- | --- | --- | --- | --- | --- | --- | --- | --- | --- | --- | --- | --- | --- | --- | --- | --- | --- | --- | --- | --- | --- | --- | --- | --- | --- | --- | --- | --- | --- | --- | --- | --- | --- | --- | --- | --- | --- | --- | --- | --- | --- | --- | --- | --- | --- | --- | --- | --- | --- | --- | --- | --- | --- | --- | --- | --- | --- | --- | --- | --- | --- | --- | --- | --- | --- | --- | --- | --- | --- | --- | --- | --- | --- | --- | --- | --- | --- | --- | --- | --- | --- | --- | --- | --- | --- | --- | --- | --- | --- | --- | --- | --- | --- | --- | --- | --- | --- | --- | --- | --- | --- | --- | --- | --- | --- | --- | --- | --- | --- | --- | --- | --- | --- | --- | --- | --- | --- | --- | --- | --- | --- | --- | --- | --- | --- | --- | --- | --- | --- | --- | --- | --- | --- | --- | --- | --- | --- | --- | --- | --- | --- | --- | --- | --- | --- | --- | --- | --- | --- | --- | --- | --- | --- | --- | --- | --- | --- | --- | --- | --- | --- | --- | --- | --- | --- | --- | --- | --- | --- | --- | --- | --- | --- | --- | --- | --- | --- | --- | --- | --- | --- | --- | --- | --- | --- | --- | --- | --- | --- | --- | --- | --- | --- | --- | --- | --- | --- | --- | --- | --- | --- | --- | --- | --- | --- | --- | --- | --- | --- | --- | --- | --- | --- | --- | --- | --- | --- | --- | --- | --- | --- | --- | --- | --- | --- | --- | --- | --- | --- | --- | --- | --- | --- | --- | --- | --- | --- | --- | --- | --- | --- | --- | --- | --- | --- | --- | --- | --- | --- | --- | --- | --- | --- | --- | --- | --- | --- | --- | --- | --- | --- | --- | --- | --- | --- | --- | --- | --- | --- | --- | --- | --- | --- | --- | --- | --- | --- | --- | --- | --- | --- | --- | --- | --- | --- | --- | --- | --- | --- | --- | --- | --- | --- | --- | --- | --- | --- | --- | --- | --- | --- | --- | --- | --- | --- | --- | --- | --- | --- | --- | --- | --- | --- | --- | --- | --- | --- | --- | --- | --- | --- | --- | --- | --- | --- | --- | --- | --- | --- | --- | --- | --- | --- | --- | --- | --- | --- | --- | --- | --- | --- | --- | --- | --- | --- | --- | --- | --- | --- | --- | --- | --- | --- | --- | --- | --- | --- | --- | --- | --- | --- | --- | --- | --- | --- | --- | --- | --- | --- | --- | --- | --- | --- | --- | --- | --- | --- | --- | --- | --- | --- | --- | --- | --- | --- | --- | --- | --- | --- | --- | --- | --- | --- | --- | --- | --- | --- | --- | --- | --- | --- | --- | --- | --- | --- | --- | --- | --- | --- | --- | --- | --- | --- | --- | --- | --- | --- | --- | --- | --- | --- | --- | --- | --- | --- | --- | --- | --- | --- | --- | --- | --- | --- | --- | --- | --- | --- | --- | --- | --- | --- | --- | --- | --- | --- | --- | --- | --- | --- | --- | --- | --- | --- | --- | --- | --- | --- | --- | --- | --- | --- | --- | --- | --- | --- | --- | --- | --- | --- | --- | --- | --- | --- | --- | --- | --- | --- | --- | --- | --- | --- | --- | --- | --- | --- | --- | --- | --- | --- | --- | --- | --- | --- | --- | --- | --- | --- | --- | --- | --- | --- | --- | --- | --- | --- | --- | --- | --- | --- | --- | --- | --- | --- | --- | --- | --- | --- | --- | --- | --- | --- | --- | --- | --- | --- | --- | --- | --- | --- | --- | --- | --- | --- | --- | --- | --- | --- | --- | --- | --- | --- | --- | --- | --- | --- | --- | --- | --- | --- | --- | --- | --- | --- | --- | --- | --- | --- | --- | --- | --- | --- | --- | --- | --- | --- | --- | --- | --- | --- | --- | --- | --- | --- | --- | --- | --- | --- | --- | --- | --- | --- | --- | --- | --- | --- | --- | --- | --- | --- | --- | --- | --- | --- | --- | --- | --- | --- | --- | --- | --- | --- | --- | --- | --- | --- | --- | --- | --- | --- | --- | --- | --- | --- | --- | --- | --- | --- | --- | --- | --- | --- | --- | --- | --- | --- | --- | --- | --- | --- | --- | --- | --- | --- | --- | --- | --- | --- | --- | --- | --- | --- | --- | --- | --- | --- | --- | --- | --- | --- | --- | --- | --- | --- | --- | --- | --- | --- | --- | --- | --- | --- | --- | --- | --- | --- | --- | --- | --- | --- | --- | --- | --- | --- | --- | --- | --- | --- | --- | --- | --- | --- | --- | --- | --- | --- | --- | --- | --- | --- | --- | --- | --- | --- | --- | --- | --- | --- | --- | --- | --- | --- | --- | --- | --- | --- | --- | --- | --- | --- | --- | --- | --- | --- | --- | --- | --- | --- | --- | --- | --- | --- | --- | --- | --- | --- | --- | --- | --- | --- | --- | --- | --- | --- | --- | --- | --- | --- | --- | --- | --- | --- | --- | --- | --- | --- | --- | --- | --- | --- | --- | --- | --- | --- | --- | --- | --- | --- | --- | --- | --- | --- | --- | --- | --- | --- | --- | --- | --- | --- | --- | --- | --- | --- | --- | --- | --- | --- | --- | --- | --- | --- | --- | --- | --- | --- | --- | --- | --- | --- | --- | --- | --- | --- | --- | --- | --- | --- | --- | --- | --- | --- | --- | --- | --- | --- | --- | --- | --- | --- | --- | --- | --- | --- | --- | --- | --- | --- | --- | --- | --- | --- | --- | --- | --- | --- | --- | --- | --- | --- | --- | --- | --- | --- | --- | --- | --- | --- | --- | --- | --- | --- | --- | --- | --- | --- | --- | --- | --- | --- | --- | --- | --- | --- | --- | --- | --- | --- | --- | --- | --- | --- | --- | --- | --- | --- | --- | --- | --- | --- | --- | --- | --- | --- | --- | --- | --- | --- | --- | --- | --- | --- | --- | --- | --- | --- | --- | --- | --- | --- | --- | --- | --- | --- | --- | --- | --- | --- | --- | --- | --- | --- | --- | --- | --- | --- | --- | --- | --- | --- | --- | --- | --- | --- | --- | --- | --- | --- | --- | --- | --- | --- | --- | --- | --- | --- | --- | --- | --- | --- | --- | --- | --- | --- | --- | --- | --- | --- | --- | --- | --- | --- | --- | --- | --- | --- | --- | --- | --- | --- | --- | --- | --- | --- | --- | --- | --- | --- | --- | --- | --- | --- | --- | --- | --- | --- | --- | --- | --- | --- | --- | --- | --- | --- | --- | --- | --- | --- | --- | --- | --- | --- | --- | --- | --- | --- | --- | --- | --- | --- | --- | --- | --- | --- | --- | --- | --- | --- | --- | --- | --- | --- | --- | --- | --- | --- | --- | --- | --- | --- | --- | --- | --- | --- | --- | --- | --- | --- | --- | --- | --- | --- | --- | --- | --- | --- | --- | --- | --- | --- | --- | --- | --- | --- | --- | --- | --- | --- | --- | --- | --- | --- | --- | --- | --- | --- | --- | --- | --- | --- | --- | --- | --- | --- | --- | --- | --- | --- | --- | --- | --- | --- | --- | --- | --- | --- | --- | --- | --- | --- | --- | --- | --- | --- | --- | --- | --- | --- | --- | --- | --- | --- | --- | --- | --- | --- | --- | --- | --- | --- | --- | --- | --- | --- | --- | --- | --- | --- | --- | --- | --- | --- | --- | --- | --- | --- | --- | --- | --- | --- | --- | --- | --- | --- | --- | --- | --- | --- | --- | --- | --- | --- | --- | --- | --- | --- | --- | --- | --- | --- | --- | --- | --- | --- | --- | --- | --- | --- | --- | --- | --- | --- | --- | --- | --- | --- | --- | --- | --- | --- | --- | --- | --- | --- | --- | --- | --- | --- | --- | --- | --- | --- | --- | --- | --- | --- | --- | --- | --- | --- | --- | --- | --- | --- | --- | --- | --- | --- | --- | --- | --- | --- | --- | --- | --- | --- | --- | --- | --- | --- | --- | --- | --- | --- | --- | --- | --- | --- | --- | --- | --- | --- | --- | --- | --- | --- | --- | --- | --- | --- | --- | --- | --- | --- | --- | --- | --- | --- | --- | --- | --- | --- | --- | --- | --- | --- | --- | --- | --- | --- | --- | --- | --- | --- | --- | --- | --- | --- | --- | --- | --- | --- | --- | --- | --- | --- | --- | --- | --- | --- | --- | --- | --- | --- | --- | --- | --- | --- | --- | --- | --- | --- | --- | --- | --- | --- | --- | --- | --- | --- | --- | --- | --- | --- | --- | --- | --- | --- | --- | --- | --- | --- | --- | --- | --- | --- | --- | --- | --- | --- | --- | --- | --- | --- | --- | --- | --- | --- | --- | --- | --- | --- | --- | --- | --- | --- | --- | --- | --- | --- | --- | --- | --- | --- | --- | --- | --- | --- | --- | --- | --- | --- | --- | --- | --- | --- | --- | --- | --- | --- | --- | --- | --- | --- | --- | --- | --- | --- | --- | --- | --- | --- | --- | --- | --- | --- | --- | --- | --- | --- | --- | --- | --- | --- | --- | --- | --- | --- | --- | --- | --- | --- | --- | --- | --- | --- | --- | --- | --- | --- | --- | --- | --- | --- | --- | --- | --- | --- | --- | --- | --- | --- | --- | --- | --- | --- | --- | --- | --- | --- | --- | --- | --- | --- | --- | --- | --- | --- | --- | --- | --- | --- | --- | --- | --- | --- | --- | --- | --- | --- | --- | --- | --- | --- | --- | --- | --- | --- | --- | --- | --- | --- | --- | --- | --- | --- | --- | --- | --- | --- | --- | --- | --- | --- | --- | --- | --- | --- | --- | --- | --- | --- | --- | --- | --- | --- | --- | --- | --- | --- | --- | --- | --- | --- | --- | --- | --- | --- | --- | --- | --- | --- | --- | --- | --- | --- | --- | --- | --- | --- | --- | --- | --- | --- | --- | --- | --- | --- | --- | --- | --- | --- | --- | --- | --- | --- | --- | --- | --- | --- | --- | --- | --- | --- | --- | --- | --- | --- | --- | --- | --- | --- | --- | --- | --- | --- | --- | --- | --- | --- | --- | --- | --- | --- | --- | --- | --- | --- | --- | --- | --- | --- | --- | --- | --- | --- | --- | --- | --- | --- | --- | --- | --- | --- | --- | --- | --- | --- | --- | --- | --- | --- | --- | --- | --- | --- | --- | --- | --- | --- | --- | --- | --- | --- | --- | --- | --- | --- | --- | --- | --- | --- | --- | --- | --- | --- | --- | --- | --- | --- | --- | --- | --- | --- | --- | --- | --- | --- | --- | --- | --- | --- | --- | --- | --- | --- | --- | --- | --- | --- | --- | --- | --- | --- | --- | --- | --- | --- | --- | --- | --- | --- | --- | --- | --- | --- | --- | --- | --- | --- | --- | --- | --- | --- | --- | --- | --- | --- | --- | --- | --- | --- | --- | --- | --- | --- | --- | --- | --- | --- | --- | --- | --- | --- | --- | --- | --- | --- | --- | --- | --- | --- | --- | --- | --- | --- | --- | --- | --- | --- | --- | --- | --- | --- | --- | --- | --- | --- | --- | --- | --- | --- | --- | --- | --- | --- | --- | --- | --- | --- | --- | --- | --- | --- | --- | --- | --- | --- | --- | --- | --- | --- | --- | --- | --- | --- | --- | --- | --- | --- | --- | --- | --- | --- | --- | --- | --- | --- | --- | --- | --- | --- | --- | --- | --- | --- | --- | --- | --- | --- | --- | --- | --- | --- | --- | --- | --- | --- | --- | --- | --- | --- | --- | --- | --- | --- | --- | --- | --- | --- | --- | --- | --- | --- | --- | --- | --- | --- | --- | --- | --- | --- | --- | --- | --- | --- | --- | --- | --- | --- | --- | --- | --- | --- | --- | --- | --- | --- | --- | --- | --- | --- | --- | --- | --- | --- | --- | --- | --- | --- | --- | --- | --- | --- | --- | --- | --- | --- | --- | --- | --- | --- | --- | --- | --- | --- | --- | --- | --- | --- | --- | --- | --- | --- | --- | --- | --- | --- | --- | --- | --- | --- | --- | --- | --- | --- | --- | --- | --- | --- | --- | --- | --- | --- | --- | --- | --- | --- | --- | --- | --- | --- | --- | --- | --- | --- | --- | --- | --- | --- | --- | --- | --- | --- | --- | --- | --- | --- | --- | --- | --- | --- | --- | --- | --- | --- | --- | --- | --- | --- | --- | --- | --- | --- | --- | --- | --- | --- | --- | --- | --- | --- | --- | --- | --- | --- | --- | --- | --- | --- | --- | --- | --- | --- | --- | --- | --- | --- | --- | --- | --- | --- | --- | --- | --- | --- | --- | --- | --- | --- | --- | --- | --- | --- | --- | --- | --- | --- | --- | --- | --- | --- | --- | --- | --- | --- | --- | --- | --- | --- | --- | --- | --- | --- | --- | --- | --- | --- | --- | --- | --- | --- | --- | --- | --- | --- | --- | --- | --- | --- | --- | --- | --- | --- | --- | --- | --- | --- | --- | --- | --- | --- | --- | --- | --- | --- | --- | --- | --- | --- | --- | --- | --- | --- | --- | --- | --- | --- | --- | --- | --- | --- | --- | --- | --- | --- | --- | --- | --- | --- | --- | --- | --- | --- | --- | --- | --- | --- | --- | --- | --- | --- | --- | --- | --- | --- | --- | --- | --- | --- | --- | --- | --- | --- | --- | --- | --- | --- | --- | --- | --- | --- | --- | --- | --- | --- | --- | --- | --- | --- | --- | --- | --- | --- | --- | --- | --- | --- | --- | --- | --- | --- | --- | --- | --- | --- | --- | --- | --- | --- | --- | --- | --- | --- | --- | --- | --- | --- | --- | --- | --- | --- | --- | --- | --- | --- | --- | --- | --- | --- | --- | --- | --- | --- | --- | --- | --- | --- | --- | --- | --- | --- | --- | --- | --- | --- | --- | --- | --- | --- | --- | --- | --- | --- | --- | --- | --- | --- | --- | --- | --- | --- | --- | --- | --- | --- | --- | --- | --- | --- | --- | --- | --- | --- | --- | --- | --- | --- | --- | --- | --- | --- | --- | --- | --- | --- | --- | --- | --- | --- | --- | --- | --- | --- | --- | --- | --- | --- | --- | --- | --- | --- | --- | --- | --- | --- | --- | --- | --- | --- | --- | --- | --- | --- | --- | --- | --- | --- | --- | --- | --- | --- | --- | --- | --- | --- | --- | --- | --- | --- | --- | --- | --- | --- | --- | --- | --- | --- | --- | --- | --- | --- | --- | --- | --- | --- | --- | --- | --- | --- | --- | --- | --- | --- | --- | --- | --- | --- | --- | --- | --- | --- | --- | --- | --- | --- | --- | --- | --- | --- | --- | --- | --- | --- | --- | --- | --- | --- | --- | --- | --- | --- | --- | --- | --- | --- | --- | --- | --- | --- | --- | --- | --- | --- | --- | --- | --- | --- | --- | --- | --- | --- | --- | --- | --- | --- | --- | --- | --- | --- | --- | --- | --- | --- | --- | --- | --- | --- | --- | --- | --- | --- | --- | --- | --- | --- | --- | --- | --- | --- | --- | --- | --- | --- | --- | --- | --- | --- | --- | --- | --- | --- | --- | --- | --- | --- | --- | --- | --- | --- | --- | --- | --- | --- | --- | --- | --- | --- | --- | --- | --- | --- | --- | --- | --- | --- | --- | --- | --- | --- | --- | --- | --- | --- | --- | --- | --- | --- | --- | --- | --- | --- | --- | --- | --- | --- | --- | --- | --- | --- | --- | --- | --- | --- | --- | --- | --- | --- | --- | --- | --- | --- | --- | --- | --- | --- | --- | --- | --- | --- | --- | --- | --- | --- | --- | --- | --- | --- | --- | --- | --- | --- | --- | --- | --- | --- | --- | --- | --- | --- | --- | --- | --- | --- | --- | --- | --- | --- | --- | --- | --- | --- | --- | --- | --- | --- | --- | --- | --- | --- | --- | --- | --- | --- | --- | --- | --- | --- | --- | --- | --- | --- | --- | --- | --- | --- | --- | --- | --- | --- | --- | --- | --- | --- | --- | --- | --- | --- | --- | --- | --- | --- | --- | --- | --- | --- | --- | --- | --- | --- | --- | --- | --- | --- | --- | --- | --- | --- | --- | --- | --- | --- | --- | --- | --- | --- | --- | --- | --- | --- | --- | --- | --- | --- | --- | --- | --- | --- | --- | --- | --- | --- | --- | --- | --- | --- | --- | --- | --- | --- | --- | --- | --- | --- | --- | --- | --- | --- | --- | --- | --- | --- | --- | --- | --- | --- | --- | --- | --- | --- | --- | --- | --- | --- | --- | --- | --- | --- | --- | --- | --- | --- | --- | --- | --- | --- | --- | --- | --- | --- | --- | --- | --- | --- | --- | --- | --- | --- | --- | --- | --- | --- | --- | --- | --- | --- | --- | --- | --- | --- | --- | --- | --- | --- | --- | --- | --- | --- | --- | --- | --- | --- | --- | --- | --- | --- | --- | --- | --- | --- | --- | --- | --- | --- | --- | --- | --- | --- | --- | --- | --- | --- | --- | --- | --- | --- | --- | --- | --- | --- | --- | --- | --- | --- | --- | --- | --- | --- | --- | --- | --- | --- | --- | --- | --- | --- | --- | --- | --- | --- | --- | --- | --- | --- | --- | --- | --- | --- | --- | --- | --- | --- | --- | --- | --- | --- | --- | --- | --- | --- | --- | --- | --- | --- | --- | --- | --- | --- | --- | --- | --- | --- | --- | --- | --- | --- | --- | --- | --- | --- | --- | --- | --- | --- | --- | --- | --- | --- | --- | --- | --- | --- | --- | --- | --- | --- | --- | --- | --- | --- | --- | --- | --- | --- | --- | --- | --- | --- | --- | --- | --- | --- | --- | --- | --- | --- | --- | --- | --- | --- | --- | --- | --- | --- | --- | --- | --- | --- | --- | --- | --- | --- | --- | --- | --- | --- | --- | --- | --- | --- | --- | --- | --- | --- | --- | --- | --- | --- | --- | --- | --- | --- | --- | --- | --- | --- | --- | --- | --- | --- | --- | --- | --- | --- | --- | --- | --- | --- | --- | --- | --- | --- | --- | --- | --- | --- | --- | --- | --- | --- | --- | --- | --- | --- | --- | --- | --- | --- | --- | --- | --- | --- | --- | --- | --- | --- | --- | --- | --- | --- | --- | --- | --- | --- | --- | --- | --- | --- | --- | --- | --- | --- | --- | --- | --- | --- | --- | --- | --- | --- | --- | --- | --- | --- | --- | --- | --- | --- | --- | --- | --- | --- | --- | --- | --- | --- | --- | --- | --- | --- | --- | --- | --- | --- | --- | --- | --- | --- | --- | --- |
